# Conformationally Restricted Glycoconjugates Derived from Arylsulfonamides and Coumarins: New Families of Tumour-Associated Carbonic Anhydrase Inhibitors

**DOI:** 10.3390/ijms24119401

**Published:** 2023-05-28

**Authors:** Mónica Martínez-Montiel, Laura L. Romero-Hernández, Simone Giovannuzzi, Paloma Begines, Adrián Puerta, Ana I. Ahuja-Casarín, Miguel X. Fernandes, Penélope Merino-Montiel, Sara Montiel-Smith, Alessio Nocentini, José M. Padrón, Claudiu T. Supuran, José G. Fernández-Bolaños, Óscar López

**Affiliations:** 1 Facultad de Ciencias Químicas, Ciudad Universitaria, Benemérita Universidad Autónoma de Puebla, Puebla 72570, PUE, Mexico; monica.martinezmon@alumno.buap.mx (M.M.-M.); laura.romerohernandez@correo.buap.mx (L.L.R.-H.); isabel.ahuja@alumno.buap.mx (A.I.A.-C.); penelope.merino@correo.buap.mx (P.M.-M.); maria.montiel@correo.buap.mx (S.M.-S.); 2 Departamento de Química Orgánica, Facultad de Química, Universidad de Sevilla, Apartado 1203, E-41071 Seville, Spain; bolanos@us.es; 3 NEUROFARBA Department, Sezione di Scienze Farmaceutiche e Nutraceutiche, University of Florence, 50019 Florence, Italy; simone.giovannuzzi@unifi.it (S.G.); pbegines@us.es (P.B.); alessio.nocentini@unifi.it (A.N.); claudiu.supuran@unifi.it (C.T.S.); 4 BioLab, Instituto Universitario de Bio-Orgánica “Antonio González” (IUBO-AG), Universidad de La Laguna, c/Astrofísico Francisco Sánchez 2, E-38206 La Laguna, Spain; apuertaa@ull.es (A.P.); mfernand@ull.es (M.X.F.); jmpadron@ull.es (J.M.P.)

**Keywords:** carbonic anhydrases, sulfonamides, coumarins, glycoconjugates, imidazolidine-2-thiones, docking

## Abstract

The involvement of carbonic anhydrases (CAs) in a myriad of biological events makes the development of new inhibitors of these metalloenzymes a hot topic in current Medicinal Chemistry. In particular, CA IX and XII are membrane-bound enzymes, responsible for tumour survival and chemoresistance. Herein, a bicyclic carbohydrate-based hydrophilic tail (imidazolidine-2-thione) has been appended to a CA-targeting pharmacophore (arylsulfonamide, coumarin) with the aim of studying the influence of the conformational restriction of the tail on the CA inhibition. For this purpose, the coupling of sulfonamido- or coumarin-based isothiocyanates with reducing 2-aminosugars, followed by the sequential acid-promoted intramolecular cyclization of the corresponding thiourea and dehydration reactions, afforded the corresponding bicyclic imidazoline-2-thiones in good overall yield. The effects of the carbohydrate configuration, the position of the sulfonamido motif on the aryl fragment, and the tether length and substitution pattern on the coumarin were analysed in the in vitro inhibition of human CAs. Regarding sulfonamido-based inhibitors, the best template turned out to be a d-*galacto*-configured carbohydrate residue, *meta*-substitution on the aryl moiety (**9b**), with *K*_i_ against CA XII within the low nM range (5.1 nM), and remarkable selectivity indexes (1531 for CA I and 181.9 for CA II); this provided an enhanced profile in terms of potency and selectivity compared to more flexible linear thioureas **1**–**4** and the drug acetazolamide (AAZ), used herein as a reference compound. For coumarins, the strongest activities were found for substituents devoid of steric hindrance (Me, Cl), and short linkages; derivatives **24h** and **24a** were found to be the most potent inhibitors against CA IX and XII, respectively (*K*_i_ = 6.8, 10.1 nM), and also endowed with outstanding selectivity (*K*_i_ > 100 µM against CA I, II, as off-target enzymes). Docking simulations were conducted on **9b** and **24h** to gain more insight into the key inhibitor–enzyme interactions.

## 1. Introduction

Carbonic anhydrases (CAs, EC 4.2.1.1) are ubiquitous metalloenzymes (most of them Zn(II)-dependent) [1] that catalyse a simple but yet essential reaction, the reversible hydration of CO_2_, to furnish HCO_3_^−^ and a proton [2]. The rate of the spontaneous, non-catalysed process was found to be pivotal in respiration [3] for the homeostasis of physiological pH [4], ureagenesis, or gluconeogenesis [5] among other biochemical events. However, it is not fast enough to meet the metabolic demand [6]. With a turnover number as high as 10^6^ s^−1^, CAs account for one of the fastest biocatalysts found in nature [7].

Carbonic anhydrases can be categorized into eight genetic families, and are named with Greek letters, that is, α, β, γ, δ, ζ, η, θ, and ι [8]; the latter family was discovered just very recently [9]. Among them, the α-CA family can be divided into 16 isoforms [10]. It is the only one found in mammals and, therefore, suitable to be used as a therapeutic target in numerous diseases, either with inhibitors or activators [11]. In this context, many α-CA inhibitors have been designed and tested against a variety of diseases, such as glaucoma [12], epilepsy [13], obesity [14], diabesity (the simultaneous presence of diabetes and obesity) [15], articular inflammatory diseases (e.g., arthritis [16]), or neuropathic pain [17]. However, undoubtedly the most pursued activity of CA inhibitors is as anticancer agents. A series of CA inhibitors are currently used as drugs, with various clinical uses as diuretics (e.g., acetazolamide), antiglaucoma agents (e.g., acetazolamide, dichlorphenamide, dorzolamide, brinzolamide), antiobesity agents (e.g., the combination of topiramate and a sympathomimetic agent), or against epilepsy (e.g., sulthiame) [18].

The human isoforms CA IX (almost absent in healthy cells) and XII are overexpressed in hypoxic tumours due to the action of the hypoxia-inducible factor-1 (HIF-1). They are responsible, together with anaerobic glycolysis, for the acidification of the tumour microenvironment, as well as for tumour survival and proliferation [19]. Moreover, CA XII inhibition has been associated [20] with the deactivation of the machinery associated to P-glycoprotein (P-gp).

The most widely studied family of CA inhibitors is comprised of Zn(II) chelators, mainly sulfonamides and their isosters sulfamates and sulfamides, which complex the metal through their deprotonated forms [21]. The cavity of the enzyme active site has an amphiphilic nature; accordingly, hydrophilic or hydrophobic interactions between the inhibitor and the active site could be established (tail approach) [22].

Coumarins (2*H*-chromen-2-ones) are secondary metabolites widely distributed in nature and considered as privileged structures in Medicinal Chemistry [23]. This is due to their numerous biological properties exhibited, including CA inhibition. The slow mode of inhibition observed for coumarins suggested that these compounds behaved as suicide inhibitors and were actually pro-drugs [24]. The intrinsic esterase activity of CAs provokes the hydrolysis of the lactone functionality of coumarins, furnishing a 2-hydroxycinnamic derivative; such a hydrolysed structure occludes the entry to the active site [24].

Moreover, the appendage of *O*-unprotected carbohydrates to pharmacophores responsible for the CA inhibition has been reported to be a valid approach for targeting selective CA IX and XII inhibition [25]. This is due to the fact that such isoforms are membrane-anchored enzymes, and the highly hydrophilic carbohydrate tail precludes the entrance of the inhibitor into the cell. This fact avoids the inhibition of cytosolic CA enzymes and can therefore improve selectivity.

**Figure 1:**
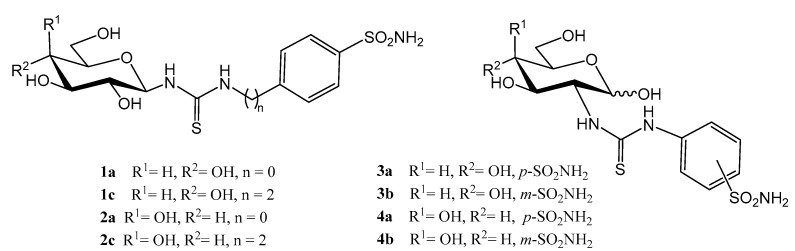
Reported flexible thiourea-containing glyco-sulfonamides.

In this context, some of us reported [26,27] the preparation of glyco-sulfonamides connected through a flexible thiourea linker (**1**–**4**, Figure 1) on C-1 or C-2 positions of the carbohydrate residue.

Our main target herein has been the conformational restriction of the carbohydrate tail of **1**–**4** and to analyse the influence of this issue on the inhibitory properties of the new compounds. For this purpose, we have used not only arylsulfonamides as Zn-chelators, but also coumarin derivatives. In this context, we have included different substitution patterns and tether lengths for the connection with the carbohydrate residue.

## 2. Results and Discussion

### 2.1. Drug Design and Chemistry

**Figure 2:**
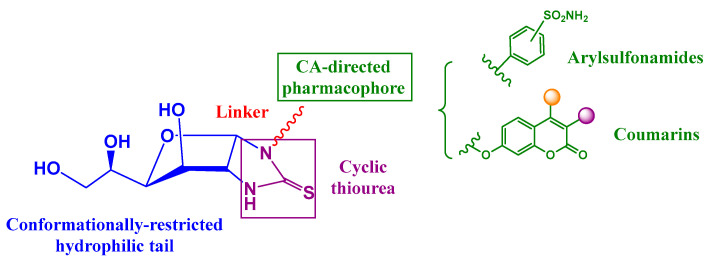
General design of the novel conformationally restricted CA glycoconjugates.

Herein, we have accomplished the preparation of a series of conformationally restricted glycoconjugates for targeting CAs. For that purpose, we envisioned the general structure depicted in Figure 2. The CA-directed pharmacophore (arylsulfonamide, coumarin) is linked to a carbohydrate residue, which acts as the hydrophilic tail, through a bicyclic thiourea (imidazolidine-2-thione).

The restriction of the conformational flexibility of a drug is a well-validated approach that might provide several advantages. It minimizes the entropy penalty in ligand–protein interactions, furnishes improved selectivity towards certain isoforms, or reduces the drug metabolization [28].

**Scheme 1:**
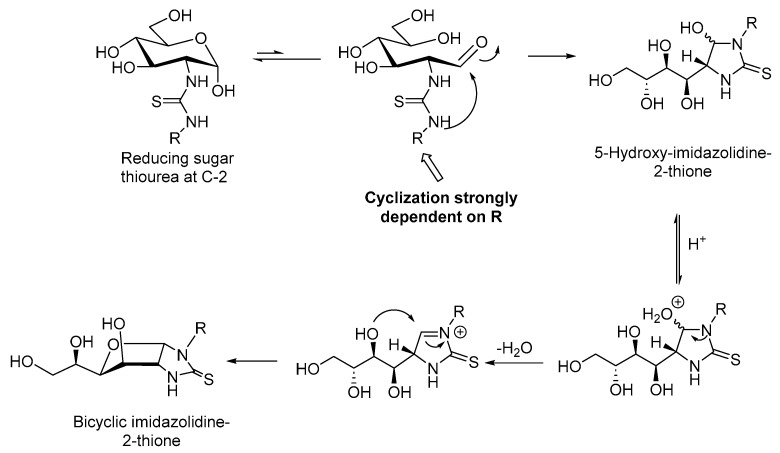
Transformation of reducing sugar thioureas on C-2 into bicyclic-imidazolidine-2-thiones.

Access to targeted bicyclic imidazolidine-2-thiones was carried out by using the methodology developed by some of us for preparing pseudo-nucleosides [29,30,31] and their selenium isosters [32]. Such a synthetic pathway involves the preparation of a transient thiourea on the C-2 of a reducing carbohydrate (Scheme 1), derived from 2-deoxy-2-amino-d-sugars. When R = arilsulfonamido, thioureas could be isolated upon coupling arylsulfonamide isothiocyanates with the corresponding *O*-unprotected 2-aminosugar [27], as no spontaneous cyclization was observed.

Nevertheless, for R = alkyl or aryl groups lacking the sulfonamido moiety, a spontaneous intramolecular cyclization was previously observed [29] to give a 5-hydroxy-imidazolidine-2-thione (Scheme 1). Such cyclization takes place through the nucleophilic attack of N-3 on the latent aldehyde moiety of the reducing sugar via a 5-*exo*-*trig* pathway according to Baldwin rules [33]. Such a compound was obtained in high stereoselectivity towards epimer 5*R* (98:2 5*R*:5*S* in DMSO-*d*_6_) starting from d-glucosamine [29] and towards 5*S* (4:96 5*R*:5*S* in CD_3_OD) starting from d-mannosamine [32]. Subsequent acid-catalysed cyclodehydration furnished (Scheme 1) the corresponding glucofurano-imidazolidine-2-thione [29] through a stabilized carbocation. As expected, the cyclization of the C-5 hydroxyl group on the carbocation to furnish a 6,5-bicyclic system was not observed, according to previous analogous compounds [29,32].

**Scheme 2:**
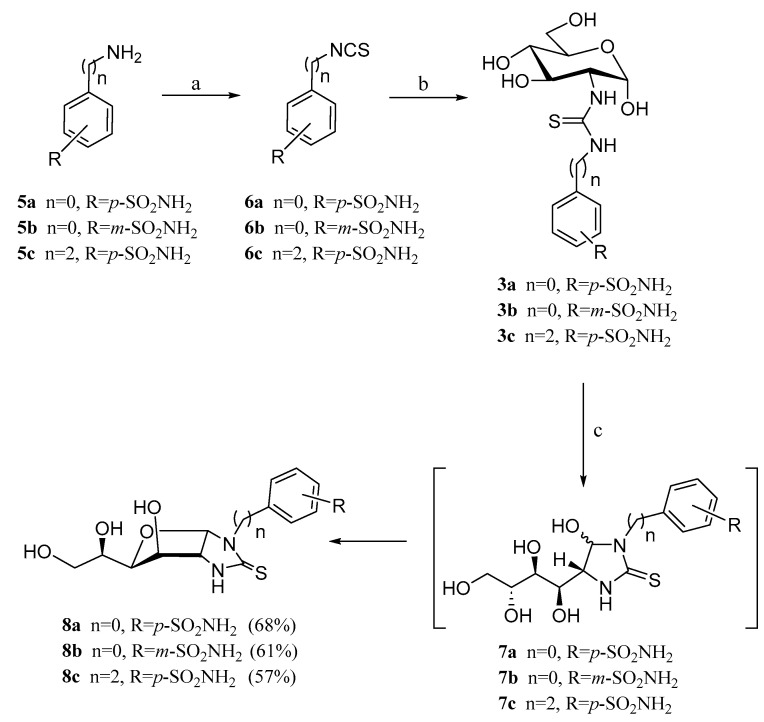
Preparation of gluco-imidazolidine-2-thiones **8a–c** derived from arylsulfonamides. Reactions and conditions: (**a**) aq. HCl, thiophosgene, rt (for **5a**, **5b**); DCC, CS_2_, Py, rt (for **5c**); (**b**) d-Glucosamine·HCl, NaHCO_3_, 2:1 EtOH–H_2_O, 75 °C; (**c**) AcOH, 2:1 EtOH–H_2_O, 90 °C.

We envisioned the possibility of transforming the reducing thioureas **3** into the corresponding bicyclic counterparts **8** (Scheme 2). For that purpose, benzenesulfonamide isothiocyanates **6** were prepared using the reported experimental conditions (thiophosgene, HCl for sulfonamides **5a**,**b** [34,35]; CS_2_, DCC for sulfonamide **5c** [36]). The structural arrangement on **8** would allow us to analyse the influence of the position of the sulfonamido motif on the aromatic ring, and of the distance between the aromatic and the carbohydrate residues on the inhibitory properties.

Coupling **6a**–**c** with d-glucosamine hydrochloride in the presence of NaHCO_3_ afforded thioureas **3a**–**c**. The preparation of targeted bicyclic derivatives **8a**–**c** was accomplished by in situ heating the thioureas at 90 °C in the presence of AcOH in aqueous EtOH. Transient 5-hydroxy-imidazolidine-2-thiones **7** were not isolated (Scheme 2).

Compounds **8a**–**c** showed an *E*_4_ conformation in the carbohydrate residue, as evidenced by vicinal coupling constants (e.g., *J*_1,2_ = 6.0 Hz, *J*_2,3_~0 Hz, *J*_3,4_ = 2.2 Hz for **8a**). Accordingly, H-1 and H-2 adopt a relative *cis* arrangement, giving a *J*_1,2_ significantly higher than expected for an α-anomer. Moreover, H-2 and H-3 are arranged with a dihedral angle close to 90°, and the exocyclic dihydroxyethyl chain exhibits conformational flexibility. ^13^C-NMR resonances at roughly 180 (CS) and 95 (C-1) ppm further confirmed the proposed structures. These data are in agreement with analogous pseudonucleosides [29].

**Scheme 3:**
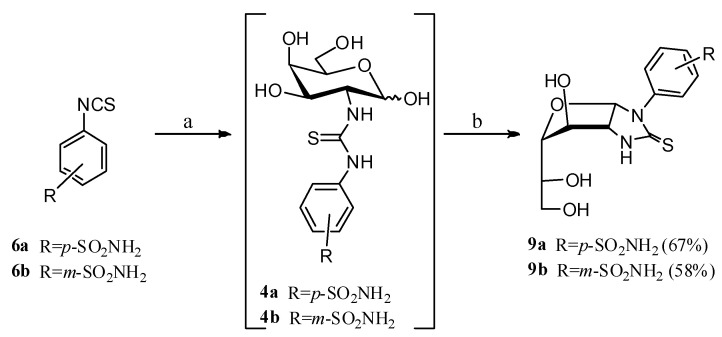
Preparation of galacto-imidazolidine-2-thiones **9a,b** derived from arylsulfonamides. Reactions and conditions: (**a**) d-Galactosamine·HCl, NaHCO_3_, 2:1 EtOH–H_2_O, 75 °C; (**b**) AcOH, 2:1 EtOH–H_2_O, 90 °C.

The same methodology was used for the preparation of d-*galacto*-configured imidazolidine-2-thiones **9a** and **9b**, using d-galactosamine hydrochloride as the reducing 2-aminosugar (Scheme 3). Attempts to extend this series to the d-*manno* configuration failed, as complex and non-resolved mixtures were obtained.

New hybrid carbohydrate-coumarins were also accessed using the above synthetic methodology. In order to establish structure–activity relationships, some key structural motifs were modulated. Thus, the carbohydrate configuration, distance between the sugar and the coumarin residues, and C-3/C-4 substitution pattern on the coumarin scaffold were accordingly modified. Moreover, some thioureas on the C-2 position were also prepared with non-reducing carbohydrates in order to analyse the influence of the bicyclic structure on the biological properties. The appendage of coumarins was accomplished on the C-7 position.

**Scheme 4:**
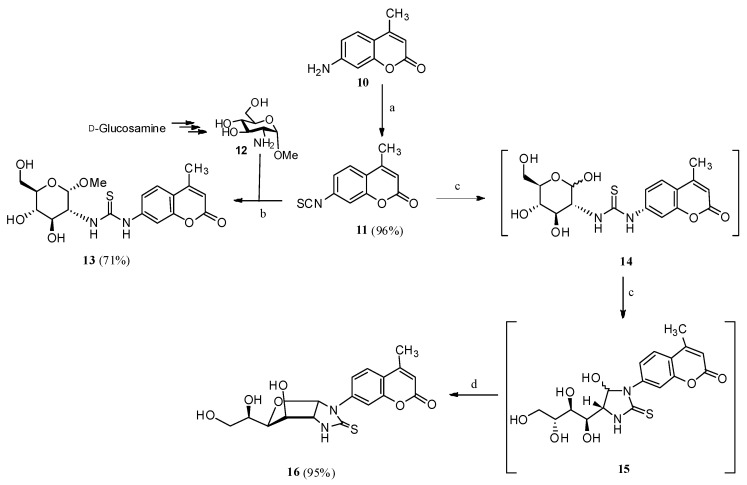
Preparation of thiourea **13** and glucofurano-imidazolidine-2-thione **16** derived from coumarins. Reactions and conditions: (**a**) CSCl_2_, CH_2_Cl_2_, Et_3_N, 35 °C; (**b**) **12**, 2:1 EtOH–H_2_O; (**c**) d-Glucosamine, NaHCO_3_, 3:1 EtOH–H_2_O, 60 °C; (**d**) AcOH, 3:1 EtOH–H_2_O, reflux.

Firstly, imidazolidine-2-thione **16** and its linear counterpart **13** were obtained in good to excellent yields (95% and 71%, respectively), using the synthetic pathway depicted in Scheme 4. These compounds lack a linker, so the coumarin residue was directly attached to the glucofurano-imidazolidine. In both cases, the key intermediate was coumarin-derived isothiocyanate **11** [37], obtained in almost quantitative yield by the treatment of commercially available 7-amino-4-methylcoumarin **10** with thiophosgene. Coupling **11** with methyl 2-amino-2-deoxy-α-d-glucopyranoside **12** furnished **13** (Scheme 4). Aminoglycoside **12** was obtained in a three-step procedure starting from d-glucosamine hydrochloride: *N*-benzoylation [38], Amberlite IR-120(H^+^)-catalysed Fischer glycosylation [39], and *N*-deprotection (NaOH). Alternatively, the coupling of **11** and d-glucosamine hydrochloride in the presence of NaHCO_3,_ followed by refluxing in aq. EtOH containing AcOH afforded imidazolidine-2-thione **16**. Its formation took place through transient thiourea **14** and 5-hydroxy-imidazolidine-2-thione **15**.

With the aim of increasing the structural diversity of the carbohydrate–coumarin template, a flexible hydrocarbon linker with different lengths was introduced on C-7. The substituents on C-3 and C-4 positions were also modified, including alkyl, aryl, and halogen fragments (H, CH_3_, Ph, Cl).

**Scheme 5:**
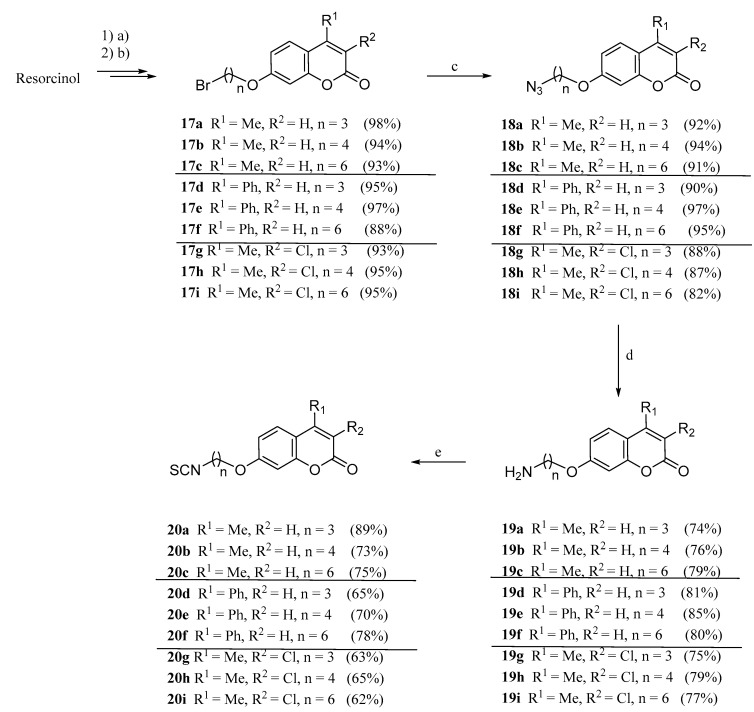
Preparation of coumarin isothiocyanates **20a**–**i**. Reactions and conditions: (**a**) β-Ketoesters, 60% H_2_SO_4_, 0 °C; (**b**) α,ω-Dibromoalkanes, K_2_CO_3_, anh. CH_3_CN, 65 °C; (**c**) NaN_3_, DMF, rt; (**d**) H_2_, Pd/C, MeOH-THF, rt; (**e**) Et_3_N, CSCl_2_, CH_2_Cl_2_, 35 °C.

For achieving such structural diversity, Pechmann condensation [40] provided three different coumarin sets; in turn, they were subjected to a Williamson synthesis with α,ω-dibromoalkanes under basic conditions (K_2_CO_3_) to furnish ω-bromoalkyl derivatives **17a**–**i**. Subsequent nucleophilic displacement with NaN_3_, Pd/C-catalysed hydrogenolysis, and isothiocyanation reaction with thiophosgene afforded isothiocyanate derivatives **20a**–**i** in good overall yields (Scheme 5).

**Scheme 6:**
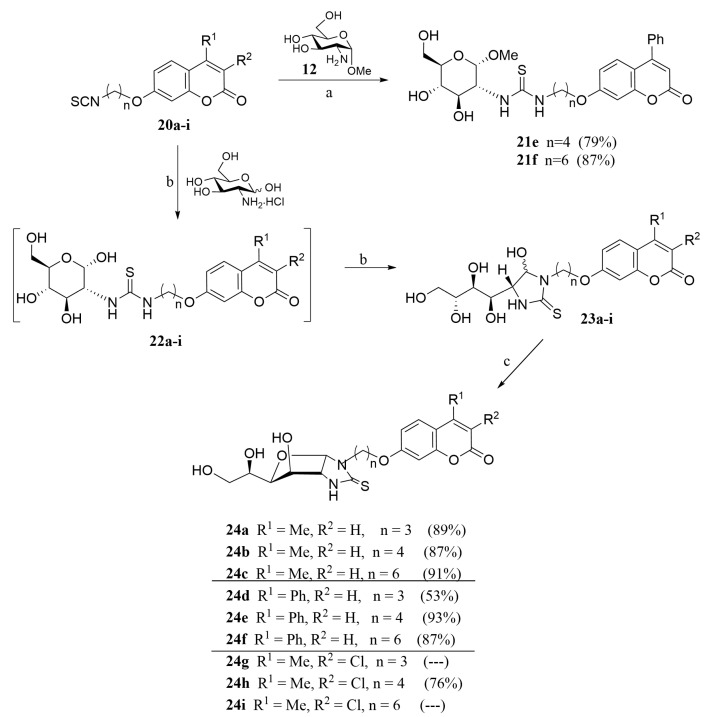
Preparation of thioureas **21e**,**f** and gluco-imidazolidine-2-thiones **24a**–**i** derived from coumarins. Reactions and conditions: (**a**) 2:1 EtOH–H_2_O, 60 °C; (**b**) NaHCO_3_, 2:1 EtOH–H_2_O, 60 °C; (**c**) AcOH, 2:1 EtOH–H_2_O, 60 °C.

Finally, isothiocyanates **20a**–**i** were transformed with excellent yields into both linear thioureas **21e**,**f**, and into bicyclic counterparts **24a**–**i** (Scheme 6). For that purpose, the same synthetic procedures as aforementioned for analogous **13** and **16** (Scheme 4) were followed. Compounds **24g**,**i** could not be isolated pure and were not included in the study.

The bicyclic scaffold of imidazolidines **24** was again supported by ^1^H-NMR data; as an example, compound **24b** exhibited *J*_1,2_ = 6.1 Hz, *J*_2,3_ ~ 0 Hz, *J*_3,4_ = 2.5 Hz.

**Scheme 7:**
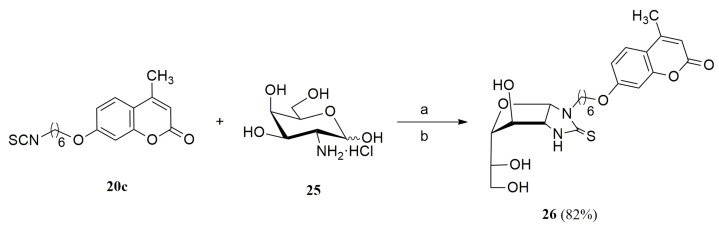
Preparation of galacto-imidazolidine-2-thione **26** derived from coumarin. Reactions and conditions: (**a**) NaHCO_3_, EtOH, 60 °C; (**b**) AcOH, EtOH, 60 °C.

We also attempted to extend this reaction to other carbohydrate configurations in order to increase the structural diversity of the potential CA inhibitors. Thus, using coumarin-derived isothiocyanate **20c** and d-galactosamine hydrochloride **25** (Scheme 7), bicyclic derivative **26** was obtained in excellent yield (82%). A strong deshielding was observed for C-4 in **26** in comparison with **24c** (87.4 vs. 79.3 ppm, respectively). This observation was reported for glycopyranosides of such configurations [41]. Unfortunately, attempts to obtain the corresponding d-*manno* isomer were again unsuccessful, and a non-resolved complex mixture was obtained.

### 2.2. Biological Assessments

#### In Vitro Carbonic Anhydrase Inhibition

The panel of CA-directed compounds prepared herein were assessed in vitro against a series of isoforms of human CA with relevant therapeutic interest. Such compounds were: arylsulfonamido-derived imidazolidine-2-thiones **8a**–**c**, **9a**,**b**, coumarin-derived imidazolidine-2-thiones **16**, **24**, **26**, and C-2 glyco-thioureas **13**, **21e**,**f**. Activities were measured using the stopped-flow CO_2_ hydration assay, and were compared with previously reported [26,27] β-d-glycopyranosyl thioureas **1**, **2**, glyco-thioureas **3**, **4**, and the drug acetazolamide (AAZ).

The CA selected for the assays can be categorized into two families: cytosolic (CA I, off-target; CA II, relevant against glaucoma [42]) and membrane-bound (CA IV, involved in rheumatoid arthritis [43]; CA IX and XII, overexpressed in hypoxic tumours [44]). The choice of such isoforms will provide information about selectivity against tumour-associated CA IX and XII compared to other relevant CAs. Promiscuous inhibitors can provoke severe side-effects. The obtained data are depicted in Table 1 (sulfonamides) and Table 2 (coumarins).

Important differences in inhibitory properties were found when comparing the carbohydrate configuration (*gluco* vs. *galacto*, compounds **8** vs. **9**), the regioisomeric position of the sulfonamido motif on the aromatic ring, and the presence or absence of the small tether connecting the bicyclic heterocycle and the arylsulfonamide scaffolds. A preference for the tumour-associated CA XII was observed in most of the bicyclic derivatives shown in Table 1, this effect being more strongly pronounced for *galacto*-derivatives **9a**,**b**. In both families of compounds, an impairment of inhibitory properties against CA I, II, and IX was observed when shifting from *para* to *meta* substitution. This was observed in a more significant fashion for the *galacto* counterparts (**9a**, **9b**), reaching submicromolar-micromolar activities for such enzymes (Table 1). Interestingly, for the latter compound, the inhibition of CA XII was kept in the low nM range (*K*_i_ = 5.1 nM), thus affording remarkable selectivities for this enzyme (I/XII = 1531; II/XII = 181.9). The selectivities found for **9b** far exceeded those found for the reference drug AAZ (I/XII = 43.9; II/XII = 2.1). Such an observation, that is, improved selectivities for the *meta* regioisomer, was also fulfilled, although to a lower extent, for *gluco* derivatives. The strong inhibition activity against CA XII exerted by **9a**,**b** was not overpassed by any of the glyco-thioureas depicted as reference compounds (**1**–**4**). Regarding activity against CA XII of the reference compounds, an opposed situation was observed for some of them. As a result, *gluco*-configured derivative was more potent than epimeric *galacto* counterparts (e.g., **1a** vs. **2a***;* **3a** vs. **4a**).

The elongation of the structure by introducing a small ethylene-type tether between the carbohydrate and the aryl sulfonamide moieties (**8a** vs. **8c**) led to an increase in activities for all the tested enzymes, except for CA IV (Table 1). Consequently, similar selectivities were found when comparing **8a** and **8c**. The latter one was proved to be a strong inhibitor of CA II (8.9 nM), an enzyme involved in glaucoma development [42].

With all the data in hand, compound **9b** can, therefore, be considered as the lead compound within the first set of imidazolidine-2-thiones derived from arylsulfonamides.

An important difference found for coumarin derivatives (Table 2) was their negligible activity towards CAs I and II, and their strong inhibition of tumour-associated membrane-anchored CA IX and XII, lying on the low- to mid-nanomolar range (6.8–177.3 nM) for CA IX; 10.1–260.3 nM for CA XII). As a result, an outstanding isoform selectivity compared to the reference drug AAZ was achieved, a fact found in some previous coumarin derivatives [45,46,47,48]. The following conclusions can be reached from the analysis of the remaining data depicted in Table 2:

- For compounds lacking linkers, almost no difference in activities can be found for non-cyclic (**13**) and bicyclic (**16**) thioureas.
- The insertion of a linker between the carbohydrate and the coumarin residues of byclicic structures proved to be benefitial for the inhibition of both membrane-bound enzymes (**16** vs. **24a**–**c**).
- The presence of a Ph residue on C-3 both in linear thioureas (**21e**,**f**) and imidazolidine-2-thiones (**24d**,**e**) provoked an impariment of the inhibitory profile against CA IX and XII, reaching the submicromolar range. This is probably due to steric clash within the active site.
- Considering the effect of the substituents (n = 4), the observed order of activity is:
  - CA IX: R^1^ = Me, R^2^ = Cl (**24h**) > R^1^ = Me, R^2^ = H (**24b**) > R^1^ = Ph, R^2^ = H (**24e**). Indeed, compound **24h** provided the strongest CA IX inhibitor of the series (*K*_i_ = 6.8 nM), roughly 3.8-fold stronger than AAZ.
  - CA XII: R^1^ = Me, R^2^ = H (**24b**) > R^1^ = Me, R^2^ = Cl (**24h**) > R^1^ = Ph, R^2^ = H (**24e**).
- The best template for the inhibition of CA XII was proved to be a short linkage (n = 3), and the monosubtitution of coumarin on C-3 with small substituents (Me, **24a**), with *K*_i_ = 10.1 nM
- Little differences in activity were found by changing the carbohydrate configuration (**24c** vs. **26**).

### 2.3. Antiproliferative Activities

The compounds prepared herein were subjected to in vitro testing as potential antiproliferative agents against a panel of six human solid tumour cell lines, following minor modifications of the protocol from the US National Cancer Institute (NCI) [49]. They can be categorized into two groups: drug-sensitive cell lines (A549, HBL-100, HeLa, SW1573) and multidrug resistant cell lines (T-47D, WiDr). Compounds that exhibited more noticeable antiproliferative activity (from moderate to good) are depicted in Table 3 (GI_50_ expressed in µM). Ph-derived thiourea **21e** and imidazolidine-2-thione **24f** were not included in the study. The remaining derivatives proved to have negligible activity at the maximum concentration tested (GI_50_ > 100 µM).

The incorporation of a phenyl moiety, in both some of the thioureas and the bicyclic counterparts (**21f** and **24e**), provided an increase in the antiproliferative potency, probably by improving the hydrophilic/hydrophobic balance for cell penetration. Interestingly, such a property was strongly dependent on the linker size, as the longer imidazolidine-2-thione derivative **24f** (n = 6) exhibited GI_50_ values > 100 µM for all the tested cell lines. Two of the tested compounds (**21f**, **24e**) exhibited strong activity on the SW1573 cell line (GI_50_ = 9.7, 5.7 µM, respectively). Thiourea **21f** also showed the best profile for the other five lines, with good GI_50_ values ranging from 23 to 36 µM.

### 2.4. Docking Simulations

**Figure 3:**
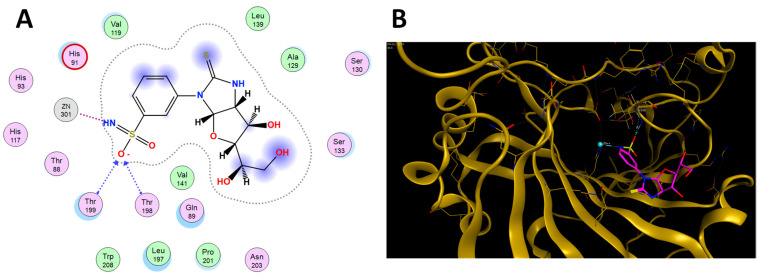
Predicted binding mode of **9b** and CA XII. (**A**) Two-dimensional view of main residues involved in the ligand–protein interactions. (**B**) Three-dimensional structure of CA XII showing the binding site.

Docking studies shed light on the molecular interactions that could take place between compounds and the different hCA isoforms. Arylsulfonamide **9b** and coumarin derivative **24h** were selected for such studies.

d-*Galacto*-configured sulfonamide **9b** was predicted to act as a zinc-chelating agent through its sulfonamido moiety (Figure 3). The deprotonated form of the sulfonamide interacts, through the NH moiety, with the Zn^2+^ cation of CA XII. A hydrogen bond interaction is also established between Thr 198, Thr 199, and the sulfonamido scaffold. In the active form of the CA, Thr 198 is hydrogen bonded with the H_2_O/OH^-^ coordinated with the zinc ion [50]. Although docking techniques do not allow the simulation of the displacement of water molecules, the interaction of **9b** directly with Zn^2+^ and the Thr 198 residue could explain the inhibitory effect towards the catalytic activity of the enzyme. The 2D-and 3D-predicted interactions of **9b** and the active site of CA XII are depicted in Figure 3A and Figure 3B, respectively.

It has been widely reported that coumarins undergo hydrolysis at the entrance of the CA active site. For that reason, both open structures (*E*- and *Z*-configured) of the coumarin derivative **24h** were considered in docking simulations [51,52].

**Figure 4:**
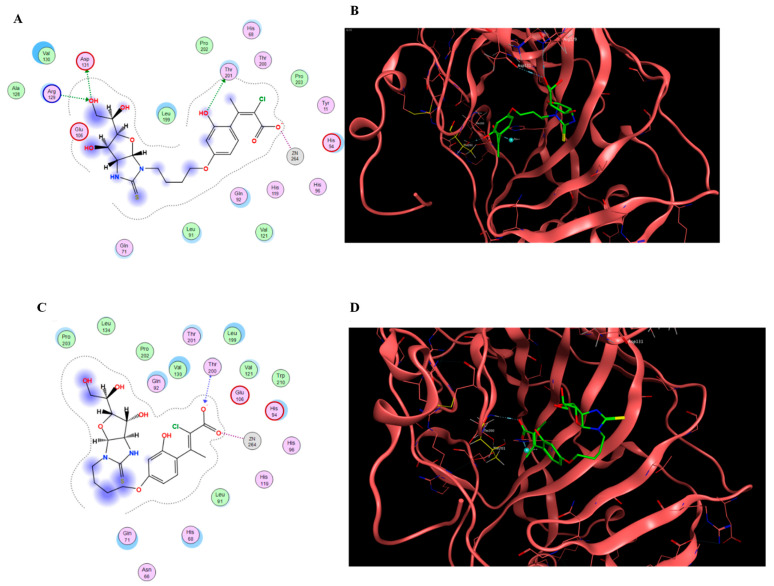
Predicted binding mode of the hydrolysed form of **24h** and CA IX. (**A**,**C**) Two-dimensional view of main residues involved in the ligand–protein interactions corresponding to closed, open *E,* and open *Z* forms, respectively. (**B**,**D**) Three-dimensional structure of CA IX showing the binding site corresponding to closed, open *E*, and open *Z* forms, respectively.

**Figure 5:**
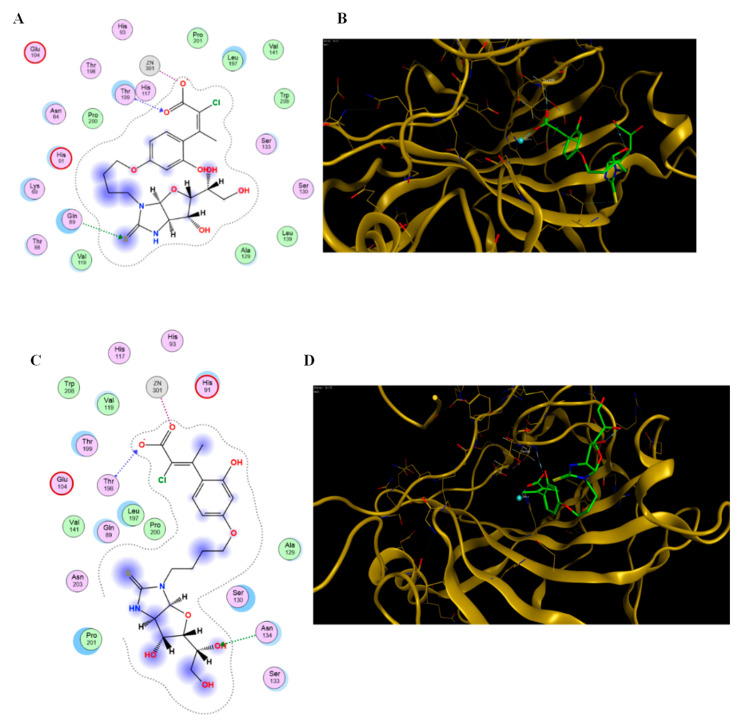
Predicted binding mode of the hydrolysed form of **24h** and CA XII. (**A**,**C**) Two-dimensional view of main residues involved in the ligand–protein interactions corresponding to closed, open *E*, and open *Z* forms, respectively. (**B**,**D**) Three-dimensional structure of CA XII showing the binding site corresponding to closed, open *E*, and open *Z* forms, respectively.

As depicted in Table 4, the binding energy scores showed the enhanced interaction of the hydroxycinnamic forms compared to the coumarin one (“closed form”). Docking simulations (Figure 4) predict that the hydrolysed product of **24h** is located inside of the binding pocket of CA IX, with the hydroxyl group interacting with Thr 201 and the carboxylate moiety interacting with Zn^2+^ (*E* form). Moreover, the *Z* form only interacts with the Thr 200 and the prosthetic Zn^2+^ cation through the carboxylate group. Similar interactions were seen in the CA XII isoform (Figure 5).

It is worth noting the specific interaction of the *Z* stereoisomer with Thr 200 and Thr 198 residues in the CA IX and CAXII, respectively, compared to the *E* counterpart, which interacts with Thr 201 and Thr 199. Moreover, the position of the tail protrudes from the active site, suggesting a possible occlusion of the entrance of the enzyme and, therefore, reducing its catalytic activity.

## 3. Materials and Methods

### 3.1. Chemistry

#### 3.1.1. General Methods

The same general procedures concerning chromatography, NMR spectroscopy, and MS spectrometry as reported previously [53] were used.

#### 3.1.2. General Procedure for the Preparation of Imidazolidine-2-Thiones **8a**–**c**, **9a**,**b**

A mixture of commercially available d-glucosamine/galactosamine hydrochloride (1.0 equiv.), NaHCO_3_ (1.0 equiv.), and the corresponding isothiocyanate **6a**–**c** (1.2 equiv.) in 2:1 H_2_O–EtOH (6 mL) was heated at 75 °C. When the starting material was consumed as evidenced by TLC, AcOH was added (1.5 mL) and the corresponding mixture was refluxed for 2 h. Then, the crude reaction mixture was concentrated to dryness, and the residue was purified by column chromatography (CH_2_Cl_2_ → 10:1 CH_2_Cl_2_–MeOH).

1-(4′-Sulfonamidophenyl)-(1″,2″-dideoxy-α-d-glucofurano)[2,1-*d*]imidazolidine-2-thione (**8a**). Isothiocyanate **6a** (117.8 mg, 0.55 mmol, 1.2 equiv.), d-glucosamine hydrochloride (100.0 mg, 0.46 mmol, 1.0 equiv.), and NaHCO_3_ (38.6 mg, 0.46 mmol, 1.0 equiv.) were used. Compound **8a** was obtained as a yellow oil. Yield: 118 mg (68%); ${\left[\alpha \right]}_{D}^{23}$+54 (*c* 0.11, MeOH); ^1^H-NMR (300 MHz, CD_3_OD) δ 7.92 (m, 2H, Ar-H), 7.79 (m, 2H, Ar-H), 6.10 (d, 1H, *J*_1″,2″_ = 6.2 Hz, H-1″), 4.35 (d, 1H, *J*_2″,3″_ = 0, H-2″), 4.34 (d, 1H, *J*_3″,4″_ = 2.2 Hz, H-3″), 3.98 (ddd, 1H, *J*_4″,5″_ = 8.3 Hz, *J*_5″,6a″_ = 2.8 Hz, *J*_5″,6b″_ = 5.6 Hz, H-5″), 3.92 (dd, 1H, H-4″), 3.80 (dd, 1H, *J*_6a″,6b″_ = 11.4 Hz, H-6a″), 3.64 (dd, 1H, H-6b″) ppm (Figure S1); ^13^C-NMR (75.5 MHz, CD_3_OD) δ 183.1 (CS), 143.6, 142.6 (C-1′, C-4′), 127.8, 127.5 (C-2′/C-6′, C-3′/C-5′), 96.4 (C-1″), 81.0 (C-4″), 75.9 (C-3″), 70.1 (C-5″), 66.9 (C-2″), 65.1 (C-6″) ppm (Figure S2); HRESI-MS *m*/*z* calcd. for C_13_H_17_N_3_NaO_6_S_2_ ([M+Na]^+^): 398.0451, found: 398.0450.

1-(3′-Sulfonamidophenyl)-(1″,2″-dideoxy-α-d-glucofurano)[2,1-*d*]imidazolidine-2-thione (**8b**). Isothiocyanate **6b** (119 mg, 0.56 mmol, 1.2 equiv.), d-glucosamine hydrochloride (100.0 mg, 0.46 mmol, 1.0 equiv.), and NaHCO_3_ (38.6 mg, 0.46 mmol, 1.0 equiv.) were used. Compound **8b** was obtained as a yellow oil. Yield: 106 mg (61%); ${\left[\alpha \right]}_{D}^{23}\;$+35 (*c* 0.15, MeOH); ^1^H-NMR (300 MHz, CD_3_OD) δ 8.11 (t, 1H, *J*_2′,4′_=*J*_2′,6′_= 1.7 Hz, H-2′), 7.84 (ddd, 1H, *J*_H,H_ = 1.1 Hz, *J*_H,H_ = 1.4 Hz, *J*_4′,5′_ = 7.8 Hz, H-4′), 7.76 (ddd, 1H, *J*_H,H_ = 1.1 Hz, *J*_H,H_ = 1.9 Hz, *J*_5′,6′_ = 8.1 Hz, H-6′), 7.57 (t, 1H, H-5′), 6.06 (d, 1H, *J*_1″,2″_ = 6.3 Hz, H-1″), 4.38 (d, 1H, *J*_2″,3″_ = 0, H-2″), 4.36 (d, 1H, *J*_3″,4″_ = 2.3 Hz, H-3″), 4.02–3.93 (m, 2H, H-4″, H-5″), 3.83 (dd, 1H, *J*_5″,6″a_ = 2.5 Hz, *J*_6a″,6b″_ = 11.4 Hz, H-6a″), 3.67 (dd, 1H, *J*_5″,6b″_ = 4.9 Hz, H-6″b) ppm (Figure S3); ^13^C-NMR (75.5 MHz, CD_3_OD) δ 183.1 (CS), 144.8, 140.3 (C-1′, C-3′), 132.2 (C-5′), 130.4 (C-6′), 126.0 (C-4′) 125.5 (C-2′), 96.2 (C-1″), 80.5 (C-4″), 75.8 (C-3″), 69.8 (C-5″), 66.9 (C-2″), 64.9 (C-6″) ppm (Figure S4); HRESI-MS *m*/*z* calcd. for C_13_H_17_N_3_NaO_6_S_2_ ([M+Na]^+^): 398.0451, found: 398.0447.

1-[2′-(4″-Sulfonamidophenyl)]ethyl-(1‴,2‴-dideoxy-α-d-glucofurano)[2,1-*d*]imidazolidine-2-thione (**8c**). Isothiocyanate **6c** (200 mg, 0.83 mmol, 1.2 equiv.), d-glucosamine hydrochloride (148.0 mg, 0.69 mmol, 1.0 equiv.), and NaHCO_3_ (58.0 mg, 0.68 mmol, 1.0 equiv.) were used. Compound **8c** was obtained as a white solid. Yield: 160 mg (57%); ${\left[\alpha \right]}_{D}^{23}\;$+12 (*c* 0.62, MeOH); ^1^H-NMR (300 MHz, CD_3_OD) δ 7.84 (m, 2H, Ar-H), 7.47 (m, 2H, Ar-H), 5.69 (d, 1H, *J*_1‴,2″″_ = 6.6 Hz, H-1‴), 4.18 (d, 1H, *J*_2‴,3‴_ = 0, *J*_3‴,4‴_ = 2.5 Hz, H-3‴), 4.07 (d, 1H, H-2‴), 3.91 (ddd, 1H, *J*_5‴,6a‴_ = 3.1 Hz, *J*_5‴,6‴b_ = 5.9 Hz, *J*_4‴,5‴_ = 8.7 Hz, H-5‴), 3.85–3.45 (m, 5H, H-4‴, H-6‴a, H-6‴b, CH_2_), 3.15 (m, 1H, CH^A^), 3.01 (m, 1H, CH^B^) ppm (Figure S5); ^13^C-NMR (75.5 MHz, CD_3_OD) δ 183.9 (CS), 145.2, 143.0 (C-1″, C-4″), 130.6, 127.3 (C-2″/C-6″, C-3″/C-5″), 94.7 (C-1‴), 80.8 (C-4‴), 76.2 (C-3‴), 70.2 (C-5‴), 66.4 (C-2‴), 65.1 (C-6″), 46.6 (N-CH_2_), 35.2 (*C*H_2_-Ar) ppm (Figure S6); ESI-MS *m*/*z* calcd. for C_15_H_21_N_3_NaO_6_S_2_ ([M+Na]^+^): 426.0764, found: 426.0760.

1-(4′-Sulfonamidophenyl)-(1″,2″-dideoxy-α-d-galactofurano)[2,1-*d*]imidazolidine-2-thione (**9a**). Isothiocyanate **6a** (117.8 mg, 0.55 mmol, 1.2 equiv.), d-galactosamine hydrochloride (100.0 mg, 0.46 mmol, 1.0 equiv.), and NaHCO_3_ (38.6 mg, 0.46 mmol, 1.0 equiv.) were used. Compound **9a** was obtained as a yellow oil. Yield: 115 mg (67%); ${\left[\alpha \right]}_{D}^{23}$+87 (*c* 0.88, DMSO); ^1^H-NMR (300 MHz, CD_3_OD) δ 7.92 (m, 4H, Ar-H), 6.10 (d, 1H, *J*_1″,2″_ = 6.7 Hz, H-1″), 4.40 (brdd, 1H, *J*_2″,3‴_ = 1.3 Hz, *J*_3″,4‴_ = 2.8 Hz, H-3″), 4.32 (dd, 1H, H-2″), 4.03 (m, 1H, H-5″)*,* 4.06–4.00 (m, 3H, H-4″, H-6a′′, H-6b″) ppm (Figure S7); ^13^C-NMR (125.7 MHz, CD_3_OD) δ 182.0 (CS), 143.7, 142.3 (C-1′, C-4′), 127.5, 127.4 (C-2′/C-6′, C-3′/C-5′), 96.8 (C-1″) 89.0 (C-4″), 77.7 (C-3″), 72.5 (C-5″), 68.1 (C-2″), 64.8 (C-6″) ppm (Figure S8).

1-(3′-Sulfonamidophenyl)-(1″,2″-dideoxy-α-d-galactofurano)[2,1-*d*]imidazolidine-2-thione (**9b**). Isothiocyanate **6b** (117.8 mg, 0.55 mmol, 1.2 equiv.), d-galactosamine hydrochloride (100.0 mg, 0.46 mmol, 1.0 equiv.), and NaHCO_3_ (38.6 mg, 0.46 mmol, 1.0 equiv.) were used. Compound **9b** was obtained as a yellow oil. Yield: 101 mg (58%); ${\left[\alpha \right]}_{D}^{23}$+85 (*c* 0.65, MeOH); ^1^H-NMR (300 MHz, CD_3_OD) δ 8.25 (t, 1H, *J*_2′,4′_= *J*_2′,6′_= 1.9 Hz, H-2′), 7.92 (ddd, 1H, *J*_H,H_ = 1.0 Hz, *J*_H,H_ = 2.1 Hz, *J*_4′,5′_= 7.9 Hz, H-4′), 7.81 (ddd, 1H, *J*_H,H_ = 1.2 Hz, *J*_H,H_ = 1.9 Hz, *J*_5′,6′_= 8.0 Hz, H-6′), 7.56 (t, 1H, H-5′), 6.08 (d, 1H, *J*_1″,2″_ = 6.5 Hz, H-1″), 4.41 (m, 1, H-3″), 4.26 (dd, 1H, H-2″), 4.41 (dd, 1H, *J*_2″,3‴_ = 1.3 Hz, *J*_3″,4‴_= 2.8 Hz, H-3″), 4.36 (dd, 1H, *J*_2″,3″_ = 1.0 Hz, H-2″), 4.05 (m, 1H, H-5″)*,* 3.70–3.58 (m, 3H, H-4″, H-6a″, H-6b″) ppm (Figure S9); ^13^C-NMR (125.7 MHz, CD_3_OD) δ 182.3 (CS), 145.1, 140.8 (C-1′, C-3′), 131.7 (C-5′), 130.2 (C-6′), 125.5, 125.2 (C-4′, C-2′), 96.9 (C-1″) 89.0 (C-4″), 77.8 (C-3″), 72.5 (C-5″), 68.1 (C-2″), 64.8 (C-6″) ppm (Figure S10); HRESI-MS *m*/*z* calcd. for C_13_H_17_N_3_NaO_6_S_2_ ([M+Na]^+^): 398.0451, found: 398.0448.

#### 3.1.3. *N*-(Methyl 2-deoxy-α-d-Glucopyranosid-2-yl)-*N*′-(4-methyl-2′-oxo-2′*H*-chromen-7′-yl)thiourea (**13**)

A mixture of coumarin-derived isothiocyanate **11** (159.0 mg, 0.73 mmol, 1.0 equiv.) and methyl 2-amino-2-deoxy-α-d-glucopyranoside **12** (141.0 mg, 0.73 mmol, 1.0 equiv.) in 2:1 EtOH–H_2_O (5 mL) was heated at 60 °C for 24 h. Then, the crude reaction mixture was concentrated to dryness and the residue was purified by column chromatography (CH_2_Cl_2_ → 10:1 CH_2_Cl_2_–MeOH) to give 13. Yield: 214 mg (71%). ^1^H-NMR (500 MHz, DMSO-d_6_) δ 10.08 (s, 1H, NH′), 8.01 (d, 1H, *J*_6′,8′_ = 1.8 Hz, H-8′), 7.92 (d, 1H, *J*_NH,2-Glc_ = 7.9 Hz, NH) 7.69 (d, 1H, *J*_5′,6′_ = 8.7 Hz, H-5′), 7.44 (dd, 1H, H-6′), 6.26 (s, 1H, H-3′), 5.12 (d, 1H, *J*_OH,4_ = 5.7 Hz, OH 4-Glc), 5.06 (d, 1H, *J*_OH,3_ = 5.8 Hz, OH 3-Glc), 4.84 (d, 1H, *J*_1,2_ = 3.5 Hz, H-1), 4.60 (t, 1H, *J*_OH,6_ = 5.9 Hz, OH 6-Glc), 4.33 (m, 1H, H-2), 3.66 (ddd, 1H, *J*_6a,6b_ = 11.8 Hz, *J*_6a,OH_ = 5.7 Hz, *J*_5,6a_ = 1.9 Hz, H-6a), 3.54 (m, 1H, H-3), 3.51 (dt, 1H, *J*_6b,OH_ = *J*_5,6b_ = 5.8 Hz, H-6b), 3.36 (m, 1H, H-5), 3.29 (s, 3H, OCH_3_), 3.22 (m, 1H, H-4), 2.40 (s, 3H, coumarin-CH_3_) ppm (Figure S11); ^13^C-NMR (125.7 MHz, DMSO-d_6_) δ 180.1 (CS), 160.1 (C-2′), 153.3 (C-9′), 153.2 (C-4′), 143.3 (C-7′), 125.5 (C-5′), 117.2 (C-6′), 115.0 (C-10′), 112.2 (C-3′), 107.3 (C-8′), 97.1 (C-1), 73.0 (C-5), 71.0 (C-3), 70.6 (C-4), 60.7 (C-6), 58.1 (C-2), 54.4 (OCH_3_), 18.1 (coumarin-CH_3_) ppm (Figure S12); HRESI-MS m/z calcd. for C_18_H_22_N_2_NaO_7_S ([M+Na]^+^): 433.1040, found: 433.1034.

#### 3.1.4. 1-(4′-Methyl-2′-oxo-2′H-chromen-7′-yl)-(1″,2″-dideoxy-α-d-glucofurano)[2,1-d]imidazolidine-2-thione (**16**)

A solution of coumarin-derived isothiocyanate **11** (215.1 mg, 0.99 mmol, 1.1 equiv.) in EtOH (5 mL) was added to a solution of d-glucosamine hydrochloride (195.0 mg, 0.90 mmol, 1.0 equiv.) and NaHCO_3_ (75.6 mg, 0.90 mmol, 1.0 equiv.) in a 3:1 EtOH-H_2_O mixture (5 mL). The resulting mixture was heated at 60 °C for 4 h; then, AcOH (154 µL, 2.7 mmol, 3.0 equiv.) was added, and refluxed for 2 h. After that, the crude reaction was concentrated to dryness and the residue was purified by column chromatography (CH_2_Cl_2_ → 10:1 CH_2_Cl_2_–MeOH) to give **16**. Yield: 323 mg (95%). ^1^H-NMR (500 MHz, DMSO-d_6_) δ 9.47 (s, 1H, NH), 7.78 (d, 1H, *J*_5′,6′_ = 8.6 Hz, H-5′), 7.69 (d, 1H, *J*_6′,8′_ = 2.0 Hz, H-8′), 7.64 (dd, 1H, H-6′), 6.87 (brq, 1H, *J*_CH3,H_ = 1.1 Hz, H-3′), 6.14 (d, 1H, *J*_1,2_ = 6.2 Hz, H-1″), 5.47 (d, *J*_OH,3_ = 4.9 Hz, 1H, OH 3-Glc), 4.80 (d, *J*_OH,5_ = 6.1 Hz, 1H, OH 5-Glc), 4.56 (t, *J*_OH,6″_ = 5.6 Hz, 1H, OH 6-Glc), 4.21 (d, 1H, *J*_2″,3″_ = 0 Hz, H-2″), 4.14 (dd, *J*_3,OH_ = 4.8 Hz, *J*_3,4_ = 2.2 Hz, 1H, H-3″), 3.74 (m, 1H, H-5″), 3.68 (dd, 1H, *J*_4″,5″_ = 8.6 Hz, *J*_3″,4″_ = 2.2 Hz, H-4″), 3.58 (ddd, 1H, *J*_6a″,6b″_ = 11.3 Hz, *J*_5″,6a″_ = 2.6 Hz, H-6a″), 3.41 (ddd, 1H, *J*_6b″,OH_ = *J*_5″,6″b_ = 5.6 Hz, H-6″b), 2.43 (d, 3H, CH_3_) ppm (Figure S13); ^13^C-NMR (125.7 MHz, DMSO-d_6_) δ 180.3 (CS), 159.9 (C-2′), 153.1 (C-4′), 152.7 (C-9′), 142.2 (C-7′), 125.2 (C-5′), 121.5 (C-6′), 117.3 (C-10′), 113.7 (C-3′), 112.7 (C-8′), 94.3 (C-1″), 79.7 (C-4″), 73.6 (C-3″), 68.1 (C-5″), 65.3 (C-2″), 63.7 (C-6″), 18.1 (CH_3_) ppm (Figure S14); HRESI-MS m/z calcd. for C_17_H_18_N_2_NaO_6_S ([M+Na]^+^): 401.0778, found: 401.0774.

#### 3.1.5. General Procedure for the Preparation of 7-Hidroxycoumarins via Pechmann Condensation

A mixture of 60% H_2_SO_4_ (23 mL) and resorcinol (1.0 g, 9.08 mmol, 1.0 equiv.) was stirred at 0 °C for 5 min; then, the corresponding β-ketoester (1.1 equiv.) was slowly added at that temperature. After the addition was completed, the mixture was stirred at rt for 4 h; then, it was poured over a water/ice mixture and the resulting precipitate was filtrated and washed with cold H_2_O. Coumarins were purified by column chromatography (7:3 hexane–EtOAc).

#### 3.1.6. General Procedure for the *O*-Alkylation of 7-Hydroxycoumarins with α,ω-Dibromoalkanes (**17a**–**i**)

To a solution of 7-hydroxycoumarins (1.0 equiv.) in dry MeCN (7 mL) was added anhydrous K_2_CO_3_ (1.5 equiv.) and the corresponding α,ω-dibromoalkanes (10.0 equiv.); the reaction mixture was heated under Ar at 65 °C for 2 h. Then, it was concentrated to dryness and the residue was purified by column chromatography (hexane→9:1 hexane–EtOAc).

#### 3.1.7. General Procedure for the Preparation of Azides **18a**–**i**

To a solution of bromoderivatives **17a**–**i** (500 mg, 1.0 equiv.) in DMF (15 mL) was added NaN_3_ (5.0 equiv.) and the corresponding mixture was stirred at rt for 2 h. Then, it was diluted with EtOAc (20 mL) and washed with brine (4 × 15 mL). The organic layer was dried over Na_2_SO_4_, filtered, and concentrated to dryness. The residue was purified by column chromatography (2:1 hexane-EtOAc) to give azidoderivatives **18a**–**i**.

7-(3′Azidopropoxy)-4-phenylcoumarin (**18d**). Bromoderivative **17d** (500 mg, 1.39 mmol) and NaN_3_ (453 mg, 6.97 mmol) were used. Yield: 400 mg (90%, solid). Mp: 67 °C; ^1^H-NMR (500 MHz, CDCl_3_) δ 7.43 (m, 3H, Ar-H), 7.36 (m, 2H, Ar-H), 7.31 (d, 1H, *J*_5,6_ = 8.9 Hz, H-5), 6.80 (s, 1H, H-8), 6.71 (d, 1H, H-6), 6.14 (s, 1H, H-3), 4.04 (m, 2H, CH_2_), 3.72 (m, 2H, CH_2_), 1.98 (m, 2H, CH_2_) ppm (Figure S15); ^13^C-NMR (125.7 MHz, CDCl_3_) δ 160.7 (C-7), 160.2 (C-2), 154.8 (C-9), 154.7 (C-4), 134.41 (Ar-C*ipso*), 128.6 (Ar-C*p*), 127.8 (x2) (Ar-C*m*), 127.3 (x2) (Ar-C*o*), 127.0 (C-5), 111.6 (C-6), 111.4 (C-10), 110.6 (C-3), 100.6 (C-8), 64.0 (C-1′), 46.9 (C-3′), 27.4 (C-2′) (Figure S16); HRESI-MS *m*/*z* calcd. for C_18_H_15_N_3_NaO_3_ ([M+Na]^+^): 344.1006, found: 344.1003.

7-(4′Azidobutoxy)-4-phenylcoumarin (**18e**). Bromoderivative **17e** (500 mg, 1.34 mmol) and NaN_3_ (435.6 mg, 6.70 mmol) were used. Yield: 435 mg (97%, solid). Mp: 35–36 °C; ^1^H-NMR (500 MHz, CDCl_3_) δ 7.52 (m, 3H, Ar-H), 7.44 (m, 2H, Ar-H), 7.38 (d, 1H, *J*_5,6_ = 8.9 Hz, H-5), 6.87 (d, 1H, *J*_6,8_ = 2.5 Hz, H-8), 6.79 (dd, 1H, H-6), 6.21 (s, 1H, H-3), 4.07 (t, 2H, *J*_H,H_ = 6.1 Hz, H-1′), 3.39 (t, 2H, *J*_H,H_ = 6.7 Hz, H-4′), 1.93 (m, 2H, H-2′), 1.81 (m, 2H, H-3′) ppm (Figure S17); ^13^C-NMR (125.7 MHz, CDCl_3_) δ 162.1 (C-7), 161.3 (C-2), 156.1 (C-9), 155.9 (C-4), 135.6 (Ar-Ci*pso*), 129.7 (Ar-C*p*), 128.9 (x2) (Ar-C*m*), 128.5 (x2) (Ar-C*o*), 128.1 (C-5), 112.7 (C-6), 112.6 (C-10), 111.9 (C-3), 101.6 (C-8), 67.9 (C-1′), 51.2 (C-4′), 26.4 (C-2′), 25.7 (C-3′) ppm (Figure S18).

7-(3′-Azidopropoxy)-3-chloro-4-methylcoumarin (**18g**). Bromoderivative **17g** (500 mg, 1.51 mmol) and NaN_3_ (490.8 mg, 7.55 mmol) were used. Yield: 390 mg (88%, solid). Mp: 62 °C; ^1^H NMR (300 MHz, CDCl_3_) δ 7.55 (d, 1H, *J*_5,6_ = 8.9 Hz, H-5), 6.92 (dd, 1H, *J*_6,8_ = 2.5 Hz, H-6), 6.85 (d, 1H, H-8), 4.14 (t, 2H, *J*_H,H_ = 6.1 Hz, H-1′), 3.55 (t, 2H, H-1′), 2.12 (s, 3H, CH_3_), 1.93 (s, 1H, H-3′), 1.82 (s, 1H, H-2′) ppm (Figure S19); ^13^C-NMR (125.7 MHz, CDCl_3_) δ 161.5 (C-7), 157.3 (C-2), 153.1 (C-9), 147.8 (C-4), 125.9 (C-5), 117.9 (C-3), 113.5 (C-10), 113.1 (C-6), 101.4 (C-8), 65.2 (C-1′), 48.0 (C-3′) 30.1 (C-2′), 16.7 (CH_3_) ppm (Figure S20); HRESI-MS *m*/*z* calcd. for C_13_H_12_ClN_3_NaO_3_ ([M+Na]^+^): 316.0459, found: 316.0456.

7-(4′-Azidobutoxy)-3-chloro-4-methylcoumarin (**18h**). Bromoderivative **17h** (500 mg, 1.45 mmol) and NaN_3_ (471.3 mg, 7.25 mmol) were used. Yield: 387 mg (87%, solid). Mp: 50–51 °C; ^1^H-NMR (500 MHz, CDCl_3_) δ 7.52 (d, 1H, *J*_5,6_ = 8.9 Hz, H-5), 6.90 (dd, 1H, *J*_6,8_ = 2.4 Hz, H-6), 6.80 (d, 1H, H-8), 4.06 (t, 2H, *J*_H,H_ = 6.1 Hz, H-1′), 3.39 (t, 2H, *J*_4′,3′_ = 6.7 Hz, H-4′), 2.55 (s, 3H, CH_3_), 2.12 (s, 3H, CH_3_), 1.93 (m, 2H, H-2′), 1.82 (m, 2H, H-3′) ppm (Figure S21); ^13^C-NMR (125.7 MHz, CDCl_3_) δ 161.9 (C-7), 157.6 (C-2), 153.2 (C-9), 148.1 (C-4), 126.0 (C-5), 117.9 (C-3), 113.4 (C-10), 113.4 (C-6), 101.3 (C-8), 68.0 (C-1′), 51.2 (C-4′), 26.4 (C-2′), 25.7 (C-3′), 16.3 (CH_3_) ppm (Figure S22).

#### 3.1.8. General Procedure for the Preparation of Amines **19a**–**i**

A mixture of the corresponding azide **18a**–**i** (500 mg) and Pd/C (50 mg) in 1:1 THF–MeOH (10 mL) was hydrogenated at 1 atm and rt for 2 h. Then, it was filtrated over a Celite^®^ pad, the filtrate was concentrated to dryness, and the residue was used directly for the next step without any further purification.

#### 3.1.9. General Procedure for the Preparation of Isothiocyanates **20a**–**i**

To a solution of aminocoumarins **19a**–**i** (500 mg, 1.0 equiv.) in CH_2_Cl_2_ (6 mL) was added Et_3_N (2.0 equiv.) and heated at 35 °C for 10 min. Then, it was cooled down to rt, and thiophosgene (3.5 equiv.) was dropwise added and heated at 35 °C for further 20 min. The crude reaction medium was concentrated to roughly half volume, furnishing an orange precipitate, which was filtrated through a Celite^®^ pad and washed with CH_2_Cl_2_. The filtrate was concentrated to dryness and the residue was purified by column chromatography (4:1 hexane–EtOAc).

4-Phenyl-7-(3′-isothiocyanatopropoxy)coumarin (**20d**). Aminocoumarin **19d** (500 mg, 1.69 mmol), Et_3_N (0.47 mL, 3.38 mmol, 2.0 equiv.), thiophosgene (0.45 mL, 5.92 mmol, 3.5 equiv.) were used. Compound **20d** was obtained as a white solid. Yield: 372 mg (65%). Mp: 127 °C; ^1^H-NMR (300 MHz, CDCl_3_) δ 7.45 (m, 3H, Ar-H), 7.38 (m, 2H, Ar-H), 7.34 (d, 1H, *J*_5,6_ = 9.0 Hz, H-5), 6.81 (d, 1H, *J*_6,8_ = 2.5 Hz, H-8), 6.75 (dd, 1H, H-6), 6.12 (s, 1H, H-3), 4.09 (m, 2H, H-1′), 3.73 (m 2H, H-3′), 2.13 (m, 2H, H-2′) ppm (Figure S23); ^13^C-NMR (125.7 MHz, CDCl_3_) δ 161.9 (C-7), 161.1 (C-2), 160.1 (C-9), 154.6 (C-4), 152.3 (Ar-C*ipso*), 130.6 (NCS), 129.8 (Ar-C*p*), 128.3 (x2) (Ar-C*m*), 128.1 (x2) (Ar-C*o*), 127.6 (C-5), 113.2 (C-10), 111.8 (C-6), 111.2 (C-3), 100.9 (C-8), 64.5 (C-1′), 41.6 (C-3′), 35.8 (C-2′) ppm (Figure S24); HRESI-MS *m*/*z* calcd. for C_19_H_15_NNaO_3_S ([M+Na]^+^): 360.0665, found: 360.0661.

4-Phenyl-7-(4′-isothiocyanatobutoxy)coumarin (**20e**). Aminocoumarin **19e** (500 mg, 1.62 mmol), Et_3_N (0.45 mL, 3.24 mmol, 2.0 equiv.), thiophosgene (0.43 mL, 5.67 mmol, 3.5 equiv.) were used. Compound **20e** was obtained as a white solid. Yield: 400 mg (70%). Mp: 141 °C; ^1^H-NMR (500 MHz, CDCl_3_) δ 7.52 (m, 3H, Ar-H), 7.44 (m, 2H, Ar-H), 7.39 (d, 1H, *J*_5,6_ = 8.9 Hz, H-5), 6.87 (d, 1H, *J*_6,8_ = 2.5 Hz, H-8), 6.79 (dd, 1H, H-6), 6.21 (s, 1H, H-3), 4.09 (t, 2H, *J*_H,H_ = 5.5 Hz, H-1′), 3.64 (t, 2H, *J*_H,H_ = 6.1 Hz, H-4′), 1.98 (m, 2H, H-2′), 1.94 (m, 2H, H-3′) ppm (Figure S25); ^13^C-NMR (125.7 MHz, CDCl_3_) δ 162.0 (C-7), 161.3 (C-2), 156.1 (C-9), 155.9 (C-4), 135.6 (Ar-C*ipso*), 130.6 (NCS), 129.7 (Ar-C*p*), 128.9 (x2) (Ar-C*m*), 128.5 (x2) (Ar-C*o*), 128.2 (C-5), 112.7 (C-10), 112.6 (C-6), 112.0 (C-3), 101.7 (C-8), 67.6 (C-1′), 44.9 (C-4′), 27.0 (C-3′), 26.3 (C-2′) ppm (Figure S26).

3-Chloro-7-(4′-isothiocyanatobutoxy)-4-methylcoumarin (**20h**). Aminocoumarin **19h** (500 mg, 1.77 mmol), Et_3_N (0.49 mL, 3.54 mmol, 2.0 equiv.), thiophosgene (0.48 mL, 6.20 mmol, 3.5 equiv.) were used. Compound **20h** was obtained as a white solid. Yield: 373 mg (65%). Mp: 62 °C; ^1^H-NMR (300 MHz, CDCl_3_) δ 7.45 (d, 1H, *J*_5,6_ = 8.9 Hz, H-5), 6.83 (dd, 1H, *J*_6,8_ = 2.4 Hz, H-6), 6.73 (d, 1H, H-8), 4.01 (t, 2H, *J*_H,H_ = 6.1 Hz, H-1′), 3.59 (t, 2H, *J*_H,H_ = 6.7 Hz, H-4′), 2.10 (s, 3H, CH_3_), 1.89 (m, 2H, H-2′), 1.22 (m, 2H, H-3′) ppm (Figure S27); ^13^C-NMR (125.7 MHz, CDCl_3_) δ 161.6 (C-7), 157.3 (C-2), 153.0 (C-9), 147.9 (C-4), 125.9 (C-5), 117.9 (C-3), 113.4 (C-10), 113.1 (C-6), 101.3 (C-8), 67.6 (C-1′), 44.8 (C-4′), 26.8 (C-2′), 26.1 (C-3′), 16.1 (CH_3_) ppm (Figure S28); HRESI-MS *m*/*z* calcd. for C_15_H_14_ClNNaO_3_S ([M+Na]^+^): 346.0275, found: 346.0272.

#### 3.1.10. General Procedure for the Preparation of Coumarin-Derived Glycol-Thioureas **21e**,**f**

To a solution of coumarin isothiocyanate **20e**,**f** (1.0 equiv.) in EtOH at 60 °C was added a solution of methyl glycoside 12 (1.0 equiv.) and Et_3_N (0.50 mL, 3.60 mmol) in H_2_O. The resulting mixture was heated at 60 °C during the time indicated in each case. Then, the crude reaction was concentrated to dryness and the residue was purified by column chromatography (40:1 CH_2_Cl_2_–MeOH) to give thioureas **21e,f** as white solids.

*N*-(Methyl 2-deoxy-α-d-glucopyranosid-2-yl)-*N′*-{4′-[7″-(4″-phenyl-2″-oxo-2″*H*-chromen-7″-yl)oxy]butyl}thiourea (**21e**). Isothiocyanate **20e** (100 mg, 0.28 mmol) in EtOH (15 mL), methyl glycoside **12** (54.1 mg, 0.28 mmol, 1.0 equiv.), and Et_3_N (0.50 mL, 3.6 mmol, 12.9 equiv.) in H_2_O (5 mL) were used. Reaction proceeded for 8.5 h. Yield: 120 mg (79%). ${\left[\alpha \right]}_{D}^{23}$+66 (*c* 0.15, DMSO); mp: 165 °C; ^1^H-NMR (300 MHz, DMSO-*d*_6_–(CD_3_)_2_CO) δ 7.94 (m, 1H, NH), 7.63–7.46 (m, 5H, Ar-H Ph), 7.35 (d, 1H, *J*_5″,6″_ = 8.9 Hz, H-5″), 7.16 (brs, 1H, NH), 7.09 (d, 1H, *J*_6″,8″_ = 2.4 Hz, H-8″), 6.94 (dd, 1H, H-6″), 6.23 (s, 1H, H-3″), 5.01 (brs, 1H, OH), 4.87 (brs, 1H, OH), 4.75 (brd, 1H, *J*_1,2_ = 3.4 Hz, H-1), 4.52 (brt, 1H, *J*_OH,6_ = 5.6 Hz, OH 6-Glc), 4.21 (brs, 1H, H-2), 4.13 (t, 1H, *J*_H,H_ = 6.4 Hz, CH_2_), 3.65 (dd, 1H, *J*_5,6a_ = 4.6 Hz, *J*_6a,6b_ = 11.3 Hz, H-6a), 3.51–3.46 (m, 2H, H-6b, H-3), 3.28 (m, 1H, H-5), 3.24 (s, 3H, OMe), 3.17 (m, 1H, H-4), 1.77 (quint, 2H, *J*_H,H_ = 6.8 Hz, CH_2_), 1.64 (quint, 2H, *J*_H,H_ = 6.5 Hz, CH_2_) ppm (Figure S29); ^13^C-NMR (75.5 MHz, DMSO-*d*_6_) δ 161.9, 160.0 (C-2″, C-7″), 155.5, 155.2 (C-4″, C-9″), 135.0 (Ar-C*ipso*, Ph) 129.6, 129.2, 128.8, 128.5, 128.4, 127.8 (Ar-C), 112.7, 111.7, 111.2, (C-3″, C-6″, C-10″), 101.6 (C-8″), 97.6 (C-1), 72.8 (C-5), 70.7 (C-3, C-4), 68.1 (CH_2_O), 60.8 (C-6), 54.3 (OMe), 25.9, 25.4 (CH_2_) ppm (Figure S30); HRESI-MS *m*/*z* calcd. for C_27_H_32_N_2_NaO_8_S ([M+Na]^+^): 567.1772, found: 567.1769.

*N′*-(Methyl 2-deoxy-α-d-glucopyranosid-2-yl)-*N′*-{6′-[7″-(4″-phenyl-2″-oxo-2″*H*-chromen-7″-yl)oxy]hexyl}thiourea (**21f**). Isothiocyanate **20f** (120 mg, 0.32 mmol) in EtOH (20 mL), methyl glycoside **12** (61.8 mg, 0.32 mmol, 1.0 equiv.), and Et_3_N (0.50 mL, 3.6 mmol, 11.3 equiv.) in H_2_O (7 mL) were used. Reaction proceeded for 17.5 h. Yield: 160 mg (87%). ${\left[\alpha \right]}_{D}^{23}$+58 (*c* 0.11, DMSO); mp: 148 °C; ^1^H-NMR (300 MHz, DMSO-*d*_6_) δ 7.55 (m, 6H, Ar-H, NH), 7.34 (d, 1H, *J*_5″,6″_ = 8.9 Hz, H-5″), 7.12 (brs, 1H, NH), 7.08 (d, 1H, *J*_6″,8″_= 2.4 Hz, H-8″), 6.93 (dd, 1H, H-6″), 6.23 (s, 1H, H-3″), 5.01 (d, 1H, *J*_OH,4_ = 5.5 Hz, OH 4-Glc), 4.85 (brs, OH 3-Glc), 4.74 (d, 1H, *J*_1,2_ = 3.4 Hz, H-1), 4.54 (t, 1H, *J*_OH,6_ = 5.8 Hz, OH 6-Glc), 4.19 (brs, 1H, H-2), 4.09 (t, 2H, *J*_H,H_= 6.6 Hz, OCH_2_), 3.64 (dd, 1H, *J*_5,6a_ = 5.2 Hz, *J*_6a,6b_= 11.3 Hz, H-6a), 3.49 (dd, 1H, *J*_5,6b_ = 5.5 Hz, H-6b), 3.44 (m, 1H, H-3), 3.29 (m, 1H, H-5), 3.17 (m, 1H, H-4), 3.23 (s, 3H, OCH_3_), 1.75 (quint, 2H, *J*_H,H_= 6.8 Hz, CH_2_), 1.54–1.34 (m, 6H, 3CH_2_) ppm (Figure S31); ^13^C-NMR (75.5 MHz, DMSO-*d*_6_) δ 182.7 (CS), 162.0 (C-2″), 160.0 (C-7″), 155.5 (C-9″), 155.2 (C-4″), 135.0 (Ar-C*ipso*, Ph), 129.7 (Ar-C*p*, Ph), 128.9 (Ar-C, Ph), 128.4 (Ar-C, Ph), 127.8 (C-5″), 112.8 (C-6″) 111.7 (C-10″), 111.2 (C-3″), 101.6 (C-8″), 97.7 (C-1), 72.8 (C-5), 71.3 (C-3), 70.8 (C-4), 68.3 (OCH_2_), 60.8 (C-6), 58.0 (C-2), 54.3 (OCH_3_), 43.7 (N-CH_2_), 28.7, 28.4, 26.2, 25.2 (CH_2_) ppm (Figure S32); HRESI-MS *m*/*z* calcd. for C_29_H_36_N_2_NaO_8_S ([M+Na]^+^): 595.2085, found: 595.2079.

#### 3.1.11. General Procedure for the Preparation of Coumarin-Derived Imidazolidine-2-Thiones **24a**–**i**, **26**

The same procedure indicated for compound 16 was used (Section 3.1.4.) with isothiocyanates **20a**–**i** and d-glucosamine/galactosamine hydrochlorides.

1-{3′-[7″-(4″-Methyl-2″-oxo-2″*H*-chromen-7″-yl)oxy]propyl}-(1‴,2‴-dideoxy-α-d-glucofurano)[2,1-*d*]imidazolidine-2-thione (**24a**). d-Glucosamine hydrochloride (142 mg, 0.66 mmol, 1.0 equiv.), isothiocyanate **20a** (200 mg, 0.73 mmol, 1.1 equiv.), NaHCO_3_ (55 mg, 0.66 mmol, 1.0 equiv.), and AcOH (114 µL, 1.99 mmol, 3.0 equiv.) were used. Compound **24a** was obtained as a white solid. Yield: 256 mg (89%). ${\left[\alpha \right]}_{D}^{23}$+25 (*c* 0.52, DMSO); mp: 151 °C; ^1^H-NMR (300 MHz, DMSO-*d*_6_) δ 8.69 (s, 1H, NH), 7.68 (d, *J*_5″,6″_ = 8.6 Hz, 1H, H-5″), 6.97 (m, 2H, H-6″, H-8″), 6.20 (brq, 1H, *J*_3″,CH3_ = 1.1 Hz, H-3″), 5.80 (d, 1H, *J*_1‴,2‴_ = 6.5 Hz, H-1‴), 5.34 (d, 1H, *J*_H,H_= 4.5 Hz, OH), 4.75 (m, 1H, OH), 4.45 (brt, 1H, *J*_H,H_= 5.6 Hz, OH 6-Glc), 4.39 (m, 1H), 4.11 (t, 2H, *J*_H,H_= 6.7 Hz, OCH_2_), 4.02–4.00 (m, 1H, H-3‴), 3.99 (d, 1H, H-2‴), 3.72–3.62 (m, 3H, H-6a‴, CH_2_), 3.60 (brdd, 1H, *J*_5‴,6‴b_= 4.2 Hz, *J*_6a‴,6b‴_= 11.7 Hz, H-6b‴), 3.38 (m, 1H), 2.39 (d, 3H, CH_3_), 2.08 (m, 2H, CH_2_) ppm (Figure S33); ^13^C-NMR (75.5 MHz, DMSO-*d*_6_) δ 181.7 (CS), 161.7, 160.2 (C-2″, C-7″), 154.7, 153.4 (C-4″, C-9″), 126.4 (C-5″), 113.1, 112.5, 111.1 (C-3″, C-6″, C-10″), 101.2 (C-8″), 92.6 (C-1‴), 79.3 (C-4‴), 73.9 (C-3‴), 68.1 (C-5‴), 66.2, 64.6 (C-2‴, OCH_2_), 63.8 (C-6‴), 41.1 (N-CH_2_), 29.0 (CH_2_), 18.1 (CH_3_) ppm (Figure S34); HRESI-MS *m*/*z* calcd. for C_20_H_24_N_2_NaO_7_S ([M+Na]^+^): 459.1196, found: 459.1194.

1-{4′-[7″-(4″-Methyl-2″-oxo-2″*H*-chromen-7″-yl)oxy]butyl}-(1‴,2‴-dideoxy-α-d-glucofurano)[2,1-*d*]imidazolidine-2-thione (**24b**). d-Glucosamine hydrochloride (112 mg, 0.52 mmol, 1.0 equiv.), isothiocyanate **20b** (200 mg, 0.69 mmol, 1.3 equiv.), NaHCO_3_ (44 mg, 0.52 mmol, 1.0 equiv.), and AcOH (111 µL, 1.94 mmol, 3.7 equiv.) were used. Compound **24b** was obtained as a white solid. Yield: 203 mg (87%). ${\left[\alpha \right]}_{D}^{23}\;$+47 (*c* 0.35, DMSO); mp: 128 °C; ^1^H-NMR (300 MHz, DMSO-*d*_6_) δ 8.69 (s, 1H, NH), 7.66 (d, *J*_5″,6″_ = 9.2 Hz, 1H, H-5″), 7.68 (m, 1H, NH), 6.95 (m, 2H, H-6″, H-8″), 6.20 (brq, 1H, *J*_3″,CH3_ = 1.1 Hz, H-3″), 5.78 (d, *J*_1‴,2‴_ = 6.5 Hz, 1H, H-1‴), 5.26 (d, 1H, *J*_H,H_ = 5.1 Hz, OH), 4.69 (d, 1H, *J*_H,H_= 6.1 Hz, OH), 4.43 (t, 1H, *J*_H,H_ = 5.6 Hz, OH 6-Glc), 4.10 (m, 2H, OCH_2_), 4.01 (d, 1H, *J*_3‴,4‴_= 2.5 Hz, H-3‴), 3.98 (d, 1H, *J*_2‴,3‴_= 0 Hz, H-2‴), 3.72 (m, 1H, H-5‴), 3.58 (m, 3H, H-6‴a, N-CH_2_), 3.39–3.35 (m, 2H, H-4‴, H-6b‴), 2.39 (d, 3H, CH_3_), 1.74 (m, 4H, 2CH_2_) ppm (Figure S35); ^13^C-NMR (75.5 MHz, DMSO-*d*_6_) δ 181.6 (CS), 161.7 (C-2″), 160.2 (C-7″), 154.7, 153.4 (C-4″, C-9″), 126.4 (C-5″), 113.0 (C-6″), 112.4 (C-10″), 111.0 (C-3″), 101.1 (C-8″), 92.4 (C-1‴), 79.3 (C-4‴), 74.0 (C-3‴), 68.2, 68.0 (C-5‴, OCH_2_), 64.6 (C-2‴), 63.8 (C-6‴), 43.5 (N-CH_2_), 25.8, 24.3 (CH_2_), 18.1 (CH_3_) ppm (Figure S36); HRESI-MS *m*/*z* calcd. for C_21_H_26_N_2_NaO_7_S ([M+Na]^+^): 473.1353, found: 473.1354.

1-{6′-[7″-(4″-Methyl-2″-oxo-2″*H*-chromen-7″-yl)oxy]hexyl}-(1‴,2‴-dideoxy-α-d-glucofurano)[2,1-*d*]imidazolidine-2-thione (**24c**). d-Glucosamine hydrochloride (123 mg, 0.57 mmol, 1.0 equiv.), isothiocyanate **20c** (200 mg, 0.63 mmol, 1.1 equiv.), NaHCO_3_ (48 mg, 0.57 mmol, 1.0 equiv.), and AcOH (102 µL, 1.78 mmol, 3.1 equiv.) were used. Compound **24c** was obtained as a white solid. Yield: 250 mg (91%). ${\left[\alpha \right]}_{D}^{23\;}$+77 (*c* 0.15, DMSO); mp: 87 °C; ^1^H-NMR (300 MHz, DMSO-*d*_6_) δ 8.67 (s, 1H, NH), 7.58 (m, 1H, H-5″), 6.96 (m, 2H, H-6″, H-8″), 6.21 (brq, 1H, *J*_3″,CH3_ = 1.1 Hz, H-3″), 5.79 (d, 1H, *J*_1‴,2‴_ = 6.5 Hz, H-1‴), 5.24 (d, 1H, *J*_H,H_ = 4.7 Hz, OH), 4.68 (d, 1H, *J*_H,H_= 6.5 Hz, OH), 4.43 (t, 1H, *J*_H,H_ = 5.8 Hz, OH 6-Glc), 4.01 (d, 1H, *J*_3‴,4‴_ = 2.4 Hz, H-3‴), 3.98 (d, 1H, *J*_2‴,3‴_= 0 Hz, H-2‴), 3.72 (m, 1H, H-5‴), 3.54 (m, 3H, H-6‴a, N-CH_2_), 3.37 (m, 2H, H-4‴, H-6‴b), 2.40 (d, 3H, CH_3_), 1.66 (m, 6H, 3CH_2_) ppm (Figure S37); ^13^C-NMR (75.5 MHz, DMSO-*d*_6_) δ 181.6 (CS), 161.8 (C-2″), 160.1 (C-7″), 154.7, 153.4 (C-4″, C-9″), 126.4 (C-5″), 113.0 (C-6″), 112.4 (C-10″), 111.0 (C-3″), 101.2 (C-8″), 92.3 (C-1‴), 79.3 (C-4‴), 74.0 (C-3‴), 68.2, 68.0 (C-5‴, OCH_2_), 64.6 (C-2‴), 63.8 (C-6‴), 30.7, 25.8, 24.2 (3CH_2_), 18.1 (CH_3_) ppm (Figure S38); HRESI-MS *m*/*z* calcd. for C_23_H_30_N_2_NaO_7_S ([M+Na]^+^): 501.1666, found: 501.1658.

1-{3′-[7″-(4″-Phenyl-2″-oxo-2″*H*-chromen-7″-yl)oxy]propyl}-(1‴,2‴-dideoxy-α-d-glucofurano)[2,1-*d*]imidazolidine-2-thione (**24d**). d-Glucosamine hydrochloride (116 mg, 0.54 mmol, 1.0 equiv.), isothiocyanate **20d** (200 mg, 0.59 mmol, 1.1 equiv.), NaHCO_3_ (45 mg, 0.54 mmol, 1.0 equiv.), and AcOH (97 µL, 1.70 mmol, 3.1 equiv.) were used. Compound **24d** was obtained as a white solid. Yield: 142 mg (53%). ${\left[\alpha \right]}_{D}^{23}\;$+53 (*c* 0.13, DMSO); mp: 136 °C; ^1^H-NMR (300 MHz, DMSO-*d*_6_) δ 8.72 (s, 1H, NH), 7.57 (m, 5H, Ar-H) 7.35 (d, 1H, *J*_5″,6″_ = 8.7 Hz, H-5″), 7.08 (d, 1H, *J*_6″,8″_ = 2.2 Hz, H-8″), 6.94 (dd, 1H, H-6″), 6.23 (s, 1H, H-3″), 5.79 (d, 1H, *J*_1‴,2‴_ = 6.7 Hz, H-1‴), 5.27 (d, 1H, *J*_H,H_= 4.8 Hz, OH), 4.68 (d, 1H, *J*_H,H_ = 6.2 Hz, OH), 4.43 (m, 1H, OH 6-Glc), 4.13 (m, 2H, OCH_2_), 4.00 (m, 2H, H-2‴, H-3‴), 3.67 (m, 4H, H-5‴, H-6‴a, CH_2_), 3.38 (m, 2H, H-6b‴, H-4‴) ppm (Figure S39); HRESI-MS *m*/*z* calcd. for C_25_H_26_N_2_NaO_7_S ([M+Na]^+^): 521.1353, found: 521.1345.

1-{4′-[7″-(4″-Phenyl-2″-oxo-2″*H*-chromen-7″-yl)oxy]butyl}-(1‴,2‴-dideoxy-α-d-glucofurano)[2,1-*d*]imidazolidine-2-thione (**24e**). d-Glucosamine hydrochloride (111 mg, 0.51 mmol, 1.0 equiv.), isothiocyanate **20e** (200 mg, 0.57 mmol, 1.1 equiv.), NaHCO_3_ (42.8 mg, 0.51 mmol, 1.0 equiv.), and AcOH (93 µL, 1.63 mmol, 3.2 equiv.) were used. Compound **24e** was obtained as a white solid. Yield: 244 mg (93%). ${\left[\alpha \right]}_{D}^{23}\;$+70 (*c* 0.21, DMSO); ^1^H-NMR (300 MHz, DMSO-*d*_6_) δ 8.69 (s, 1H, NH), 7.55 (m, 5H, Ar-H, Ph) 7.34 (d, 1H, *J*_5″,6″_ = 8.6 Hz, H-5″), 7.08 (d, 1H, *J*_6″,8″_ = 2.4 Hz, H-8″), 6.94 (dd, 1H, H-6″), 6.23 (s, 1H, H-3″), 5.78 (d, 1H, *J*_1‴,2‴_ = 6.5 Hz, H-1‴), 5.24 (d, 1H, *J*_H,H_ = 5.0 Hz, OH), 4.68 (d, 1H, *J*_H,H_ = 6.1 Hz, OH), 4.43 (t, 1H, *J*_H,H_= 5.7 Hz, OH 6-Glc), 4.13 (brt, 2H, *J*_H,H_ = 5.4 Hz, OCH_2_) 4.01 (d, 1H, *J*_3‴,4‴_= 2.3 Hz, H-3‴), 3.98 (d, *J*_2‴,3‴_ = 0 Hz, H-2‴), 3.72 (m, 1H, H-5‴), 3.59 (m, 3H, H-6‴a, N-CH_2_), 3.38 (m, 2H, H-6‴b, H-4‴), 1.75 (m, 4H, 2CH_2_) ppm (Figure S40); ^13^C-NMR (75.5 MHz, DMSO-*d*_6_) δ 181.7 (CS), 161.9, 160.0 (C-2″, C-7″), 155.5, 155.2 (C-4″, C-9″), 135.0 (Ar-C*ipso*, Ph), 129.6 (Ar-C*p*, Ph), 128.6, 128.4 (Ar-C*o*, Ar-C*m*, Ph), 127.8 (C-5″), 112.8, 111.7, 111.2 (C-3″, C-6″, C-10″), 101.6 (C-8″), 92.3 (C-1‴), 79.3 (C-4‴), 74.0 (C-3‴), 68.2, 68.1 (OCH_2_, C-5‴), 64.6 (C-2‴), 63.8 (C-6‴), 43.5 (C-4´) 25.8 (C-2´), 24.3 (C-3´) ppm (Figure S41); HRESI-MS *m*/*z* calcd. for C_26_H_28_N_2_NaO_7_S ([M+Na]^+^): 535.1509, found: 535.1509.

1-{6′-[7″-(4″-Phenyl-2″-oxo-2″*H*-chromen-7″-yl)oxy]hexyl}-(1‴,2‴-dideoxy-α-d-glucofurano)[2,1-*d*]imidazolidine-2-thione (**24f**). d-Glucosamine hydrochloride (100 mg, 0.46 mmol, 1.0 equiv.), isothiocyanate **20f** (200 mg, 0.53 mmol, 1.2 equiv.), NaHCO_3_ (38.6 mg, 0.46 mmol, 1.0 equiv.), and AcOH (83 µL, 1.45 mmol, 2.6 equiv.) were used. Compound **24f** was obtained as a white solid. Yield: 216 mg (87%). ${\left[\alpha \right]}_{D}^{23}\;$+70 (*c* 0.21, DMSO); ^1^H-NMR (300 MHz, DMSO-*d*_6_) δ 8.63 (s, 1H, NH), 7.56 (m, 5H, Ar-H, Ph), 7.34 (d, 1H, *J*_5″,6″_ = 8.9 Hz, H-5″), 7.08 (d, 1H, *J*_6″,8″_ = 2.4 Hz, H-8″), 6.94 (dd, 1H, H-6″), 6.22 (s, 1H, H-3″), 5.76 (d, 1H, *J*_1‴,2‴_ = 6.5 Hz, H-1‴), 5.25 (d, 1H, *J*_H,H_ = 4.9 Hz, OH), 4.69 (d, 1H, *J*_H,H_ = 6.2 Hz, OH), 4.43 (t, 1H, *J*_H,H_ = 5.7 Hz, OH 6-Glc), 4.08 (brt, 2H, *J*_H,H_= 6.4 Hz, OCH_2_), 4.00 (d, 1H, *J*_3‴,4‴_= 2.4 Hz, H-3‴), 3.97 (d, 1H, *J*_2‴,3‴_ = 0 Hz, H-2‴), 3.72 (m, 1H, H-5‴), 3.57 (m, 1H, H-6‴a), 3.45 (brt, *J*_H,H_ = 7.1 Hz, N-CH_2_), 3.35 (m, 2H, H4‴, H-6‴b), 1.75 (quint, 2H, *J*_H,H_ = 6.6 Hz, CH_2_), 1.66–1.29 (m, 6H, 3CH_2_) ppm (Figure S42); ^13^C-NMR (75.5 MHz, DMSO-*d*_6_) δ 181.5 (CS), 161.9, 160.0 (C-2″, C-7″), 155.4, 155.1 (C-4″, C-9″), 135.0 (Ar-C*ipso*, Ph), 129.5 (Ar-C*p*, Ph), 128.8, 128.3 (Ar-C*o*, Ar-C*m*, Ph), 127.7 (C-5″), 112.7, 111.6, 111.1 (C-3″, C-6″, C-10″), 101.6 (C-8″), 92.3 (C-1‴), 79.2 (C-4‴), 73.9 (C-3‴), 68.3, 68.2 (OCH_2_, C5‴), 64.4 (C-2‴), 63.8 (C-6‴), 43.7 (N-CH_2_), 28.3, 27.4, 25.9, 25.1 (4CH_2_) ppm (Figure S43); HRESI-MS *m*/*z* calcd. para C_28_H_32_N_2_NaO_7_S ([M+Na]^+^): 563.1822, found: 563.1811.

1-{4′-[7″-(3″-Chloro-4″-methyl-2″-oxo-2″*H*-chromen-7″-yl)oxy]butyl}-(1‴,2‴-dideoxy-α-d-glucofurano)[2,1-*d*]imidazolidine-2-thione (**24h**). d-Glucosamine hydrochloride (126 mg, 0.58 mmol, 1.0 equiv.), isothiocyanate **20h** (200 mg, 0.62 mmol, 1.1 equiv.), NaHCO_3_ (48.7 mg, 0.58 mmol, 1.0 equiv.), and AcOH (105 µL, 1.84 mmol, 3.2 equiv.) were used. Compound **24h** was obtained as a white solid. Yield: 214 mg (76%). ${\left[\alpha \right]}_{D}^{23}\;$+45 (*c* 0.30, DMSO); mp: 127 °C; ^1^H-NMR (300 MHz, DMSO-*d*_6_) δ 8.58 (s, 1H, NH), 7.73 (m, 1H, H-5″), 7.00 (m, 2H, H-6″, H-8″), 5.78 (d, 1H, *J*_1‴,2‴_ = 6.8 Hz, H-1‴), 5.26 (d, 1H, *J*_H,H_= 4.4 Hz, OH), 4.69 (d, 1H, *J*_H,H_= 6.8 Hz, OH), 4.43 (t, 1H, *J*_H,H_= 5.7 Hz, OH 6-Glc), 4.11 (m, 2H, OCH_2_), 4.00 (d, 1H, *J*_3‴,4‴_= 2.3 Hz, H-3‴), 3.98 (d, 1H, *J*_2‴,3‴_= 0 Hz, H-2‴), 3.72 (m, 1H, H-5‴), 3.61–3.52 (m, 2H, H-6‴a, CH_2_), 3.39 (m, 2H, H-4‴, H-6‴b), 2.52 (s, 3H, CH_3_), 1.75 (m, 4H, 2CH_2_) ppm (Figure S44); ^13^C-NMR (125.7 MHz, DMSO-*d*_6_) δ 181.6 (CS), 161.7 (C-7″), 156.4 (C-2″), 152.6 (C-9″), 148.8 (C-4″), 126.9 (C-5″), 116.0 (C-3″), 113.0 (C-6″), 112.6 (C-10″), 101.1 (C-8″), 92.4 (C-1‴), 79.3 (C-4‴), 74.0 (C-3‴), 68.2 (x2) (C-5‴, OCH_2_), 64.6 (C-2‴), 63.8 (C-6‴), 43.5 (N-CH_2_), 25.8, 24.2 (CH_2_), 16.0 (CH_3_) ppm (Figure S45); HRESI-MS *m*/*z* calcd. for C_21_H_25_ClN_2_NaO_7_S ([M+Na]^+^): 507.0963, found: 507.0958.

1-{4′-[7″-(4″-Methyl-2″-oxo-2″*H*-chromen-7″-yl)oxy]hexyl}-(1‴,2‴-dideoxy-α-d-galactofurano)[2,1-*d*]imidazolidine-2-thione (**26**). d-Galactosamine hydrochloride (123 mg, 0.57 mmol, 1.0 equiv.), isothiocyanate **20c** (200 mg, 0.63 mmol, 1.1 equiv.), NaHCO_3_ (47.9 mg, 0.57 mmol, 1.0 equiv.), and AcOH (102 µL, 1.78 mmol, 3.1 equiv.) were used. Compound **26** was obtained as a white solid. Yield: 223 mg (82%). ${\left[\alpha \right]}_{D}^{23}\;$+46 (*c* 0.22, DMSO); ^1^H-NMR (500 MHz, DMSO-*d*_6_) δ 8.72 (s, 1H, NH), 7.67 (d, 1H, *J*_5″,6″_= 8.4 Hz, H-5″), 6.97 (m, 2H, H-6″, H-8″) 6.18 (br2, 1H, H-3″), 5.72 (d, 1H, *J*_1‴,2‴_ = 6.9 Hz, H-1‴), 4.06 (m, 4H, OCH_2_, H-2‴, H-3‴), 2.3 (brs, 3H, CH_3_), 1.74 (quint, 2H, *J*_H,H_= 6.9 Hz, CH_2_), 1.59, (m, 2H, CH_2_), 1.44 (m, 2H, CH_2_), 1.31 (m, 2H, CH_2_) ppm (Figure S46); ^13^C-NMR (125.7 MHz, DMSO-*d*_6_) δ 180.8 (CS), 162.0, 160.5 (C-2″, C-7″), 154.9, 153.8 (C-4″, C-9″), 126.7 (C-5″), 113.2, 112.7, 111.2 (C-6″, C-10″, C-3″), 101.3 (C-8″), 92.2 (C-1‴), 87.4 (C-4‴), 76.4 (C-3‴), 70.6 (C-5‴), 68.6 (OCH_2_). 66.0, 63.5 (C-2‴, C-6‴), 43.6 (N-CH_2_) 28.6, 27.1, 26.2, 25.4 (CH_2_), 18.1 (CH_3_) ppm (Figure S47); HRESI-MS *m*/*z* calcd. for C_23_H_30_N_2_NaO_7_S ([M+Na]^+^): 501.1666, found: 501.1661.

### 3.2. CA Inhibition Assays

The inhibitory properties of title compounds against CAs were determined using the stopped-flow CO_2_ hydrase assay, as previously reported [46]. All enzymes employed were recombinant and obtained in-house as reported, with concentrations in the assay ranging from 5 to 12 nM.

### 3.3. Antiproliferative Assays

Minor modifications of the US National Cancer Institute (NCI) protocol were used [49].

### 3.4. Docking Simulations

Structures for all proteins (CA IX: PDBid 5FL4; CA XII: PDBid 4HT2) were retrieved from the Protein DataBank. Crystal structures were optimized using QuickPrep protocol from MOE (Chemical Computing Group). All ligands were drawn, hydrogens added, and geometry optimized with MOE. To simulate conditions in the enzymatic environment, sulfonamide and open form of coumarin were deprotonated. For the docking calculations, ligands were placed in the area of co-crystalized ligand from pdb file. In the placement stage, we used the Triangle Matcher algorithm with the London dG scoring scheme. In the refinement stage, we kept the receptor rigid and used the GBVI/WSA dG scoring scheme.

## Figures and Tables

**Table 1:** Inhibition constants and selectivity indexes of sulfonamido-containing imidazolidine-2-thiones **8**, **9** against hCAs I, II, IV, IX, and XII compared with thioureas **1**–**4** and AAZ ^a^.

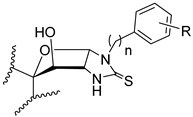										
**Compound**		*K*_i_ (nM)					S.I. ^b^			
		hCA I	hCA II	hCA IV	hCA IX	hCA XII	I/IX	I/XII	II/IX	II/XII
d-*Gluco*	**8a** (n = 0, R = *p*-SO_2_NH_2_)	765.5	60.0	347.6	175.0	502.5	4.4	1.5	0.3	0.1
	**8b** (n = 0, R = *m*-SO_2_NH_2_)	4578	5053	45.1	252.6	216.0	18.1	21.2	20.0	23.4
	**8c** (n = 2, R = *p*-SO_2_NH_2_)	84.7	**8.9** ^d^	1050	61.4	51.4	1.4	1.6	0.1	0.1
d-*Galacto*	**9a** (n = 0, R = *p*-SO_2_NH_2_)	90.3	116.0	4572	5657	**2.9**	0.02	31.1	0.02	40.0
	**9b** (n = 0, R = *m*-SO_2_NH_2_)	7807	927.6	27,250	9627	**5.1**	0.8	1531	0.1	181.9
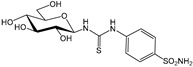 **1a** [26]		7680	7.0	--- ^c^	282	**8.2**	27.2	937	0.02	0.9
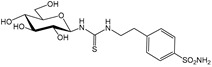 **1c** [26]		9.0	108	---	**8.7**	**9.7**	1.0	0.9	12.4	11.1
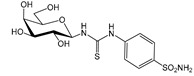 **2a** [26]		6840	222	---	**7.0**	20.1	977.1	340	31.7	11.0
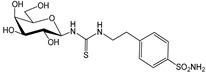 **2c** [26]		5790	9.3	---	**2.8**	**10.2**	2067.9	568	3.3	0.9
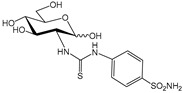 **3a** [27]		2700	9700	---	77	**7.9**	35.1	342	126	1228
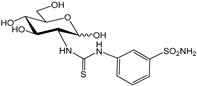 **3b** [27]		100	8600	---	**9.0**	207	11.1	0.5	956	41.5
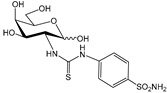 **4a** [27]		3600	7700	---	74	104	48.6	34.6	104	74.0
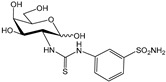 **4b** [27]		4300	940	---	42	14	102.4	307.1	22.4	67.1
**AAZ**		250	12.1	74.0	25.8	**5.7**	**9.7**	43.9	0.5	2.1

^a^ Mean from three different assays, by a stopped-flow technique (errors were in the range of ±5–10% of the reported values); ^b^ S.I. = *K*_i_ (CA I or II)/*K*_i_ (CA XI or XII); ^c^ Not tested; ^d^ Bold values indicate strong inhibition (*K*_i_ < 10.5 nM).

**Table 2:** Inhibition constants and selectivity indexes of glyco-derived imidazolidine-2-thiones **16**, **24**, **26** against hCAs I, II, IX, and XII ^a^.

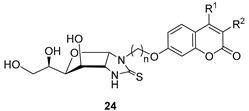								
Compound	*K*_i_ (nM)				S.I. ^b^			
	hCA I	hCA II	hCA IX	hCA XII	I/IX	I/XII	II/IX	II/XII
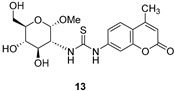	>100,000	>100,000	73.9	56.1	>1353	>1783	>1353	>1783
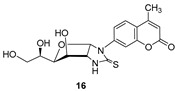	>100,000	>100,000	89.1	91.0	>1122	>1099	>1122	>1099
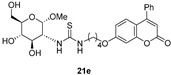	>100,000	>100,000	105.8	227.8	>945	>439	>945	>439
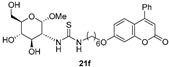	>100,000	17,300	116.5	163.9	>858	>610	148	106
**24a** R^1^ = Me, R^2^ = H, n = 3	>100,000	>100,000	45.3	**10.1** ^c^	>2208	>9901	>2208	>9901
**24b** R^1^ = Me, R^2^ = H, n = 4	>100,000	>100,000	70.7	25.7	>1414	>3891	>1414	>3891
**24c** R^1^ = Me, R^2^ = H, n = 6	>100,000	>100,000	22.4	72.1	>4464	>1387	>4464	>1387
**24d** R^1^ = Ph, R^2^ = H, n = 3	>100,000	>100,000	91.4	260.3	>1094	>384	>1094	>384
**24e** R^1^ = Ph, R^2^ = H, n = 4	>100,000	>100,000	177.3	140.4	>564	>712	>564	>712
**24h** R^1^ = Me, R^2^ = Cl, n = 4	>100,000	>100,000	**6.8**	37.5	>14,706	>2667	>14,706	>2667
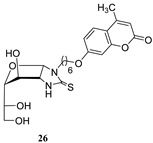	>100,000	>100,000	28.6	61.4	>3947	>1629	>3947	>1629

^a^ Mean from three different assays, by a stopped-flow technique (errors were in the range of ±5–10% of the reported values); ^b^ S.I. = *K*_i_ (CA I or II)/*K*_i_ (CA XI or XII); ^c^ Bold values indicate strong inhibition (*K*_i_ < 10.5 nM).

**Table 3:** Antiproliferative activity (GI_50_, µM) of selected compounds (mean ± SD).

Compound	Drug-Sensitive Cell Lines				Multidrug-Resistant Cell Lines	
	A549 (Lung, Non-Small)	HBL-100 (Breast)	HeLa (Cervix)	SW1573 (Lung, Non-Small)	T-47D (Breast)	WiDr (Colon)
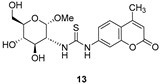	79 ± 36	86 ± 25	83 ± 30	79 ± 37	>100	>100
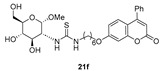	34 ± 4.0	23 ± 3.2	25 ± 8.0	**9.7** ^a^	30 ± 7.4	36 ± 16
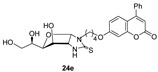	64 ± 31	23 ± 0.8	31 ± 0.2	**5.7 ± 1.8**	70 ± 23	47 ± 11

^a^ Bold values indicate strong inhibition (*K*_i_ < 10.5 nM).

**Table 4:** Docking predicted binding energies for coumarin **24h** (closed and open forms) with CAIX and CAXII (kcal/mol).

Enzyme/Compound	24h (Closed)	24h (Open *E*)	24h (Open *Z*)
hCA IX	−8.4781	−10.1987	−9.6243
hCA XII	−7.1633	−9.3104	−9.8885

## Data Availability

Not applicable.

## Supplementary Materials

The supporting information can be downloaded at: https://www.mdpi.com/article/10.3390/ijms24119401/s1.
