# Supplementary material for: Conformationally Restricted Glycoconjugates Derived from Arylsulfonamides and Coumarins: New Families of Tumour-Associated Carbonic Anhydrase Inhibitors
Source: Int J Mol Sci. 2023 May 28;24(11):9401. doi: 10.3390/ijms24119401 (PMC10253577; doi:10.3390/ijms24119401)
Supplement: Supplementary file 1 [file ijms-24-09401-s001.zip › ijms-2378858-supplementary.pdf]

SUPPORTING INFORMATION

Conformationally-restricted glycoconjugates derived from arylsulfonamides and coumarins: new families of tumour-associated carbonic anhydrase inhibitors

Mónica Martínez-Montiel [1,2], Laura L. Romero-Hernández[1], Simone Giovannuzzi[3], Paloma Begines[3], Adrián Puerta[4], Ana I. Ahuja-Casarín[1], Miguel X. Fernandes[4], Penélope Merino-Montiel[1], Sara Montiel-Smith[1], Alessio Nocentini[3], José M. Padrón[4], Claudiu T. Supuran[3], José G. Fernández-Bolaños[2] and Óscar López[2,*]

[1]Facultad de Ciencias Químicas, Ciudad Universitaria, Benemérita Universidad Autónoma de Puebla, 72570 Puebla, PUE, México

[2] Departamento de Química Orgánica, Facultad de Química, Universidad de Sevilla, Apartado 1203, Seville, E-41071, Spain

[3] NEUROFARBA Department, Sezione di Scienze Farmaceutiche e Nutraceutiche, University of Florence, Florence, 50019

[4] BioLab, Instituto Universitario de Bio-Orgánica "Antonio González" (IUBO-AG), Universidad de La Laguna, c/Astrofísico Francisco Sánchez 2, La Laguna, E-38206, Spain

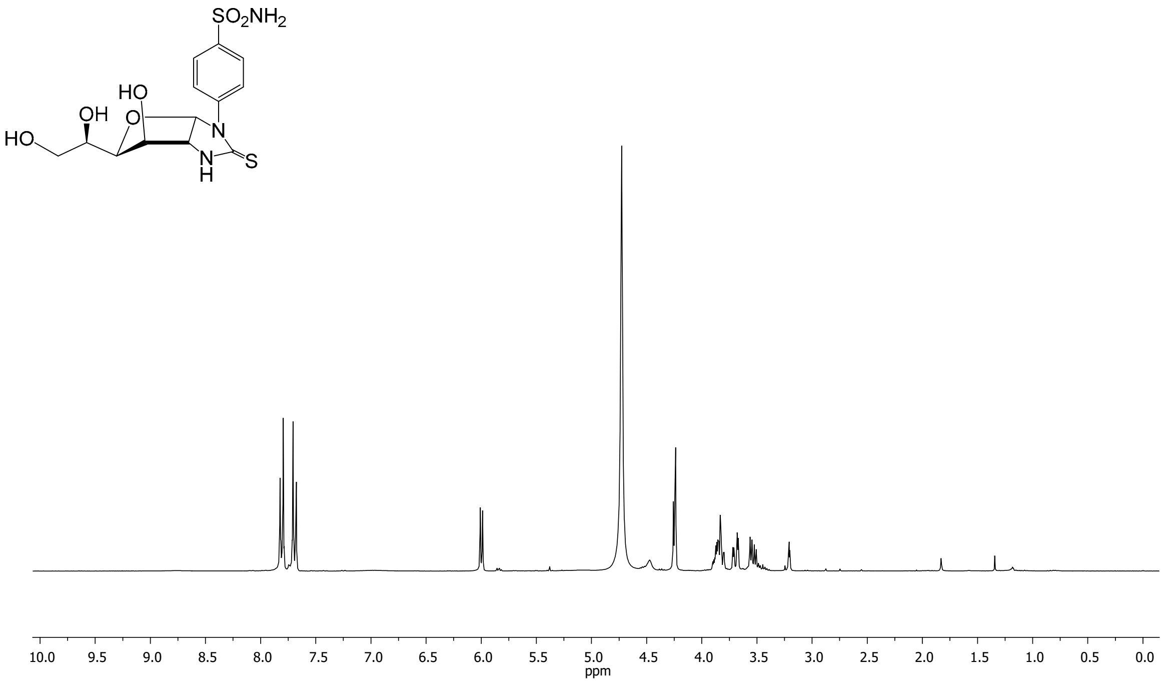

Figure S1. ^{1}H-NMR spectrum of **8a** (300 MHz, CD_3OD)

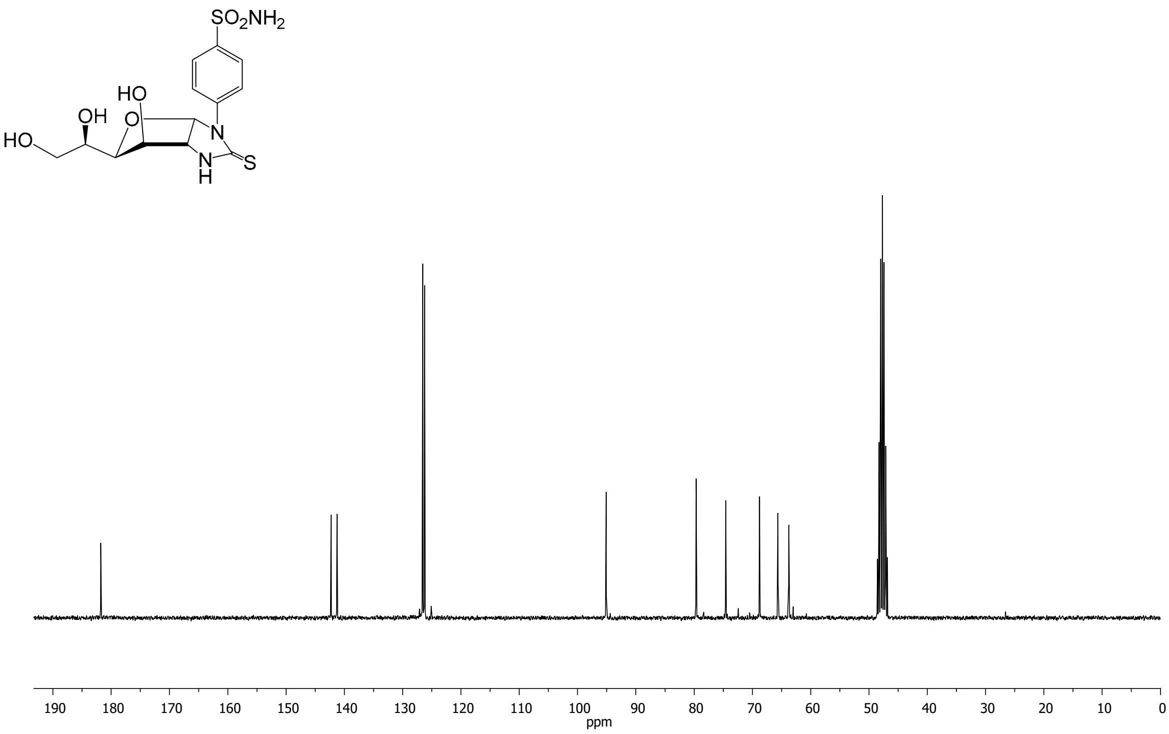

Figure S2. ^{13}C-NMR spectrum of **8a** (75.5 MHz, CD_3OD)

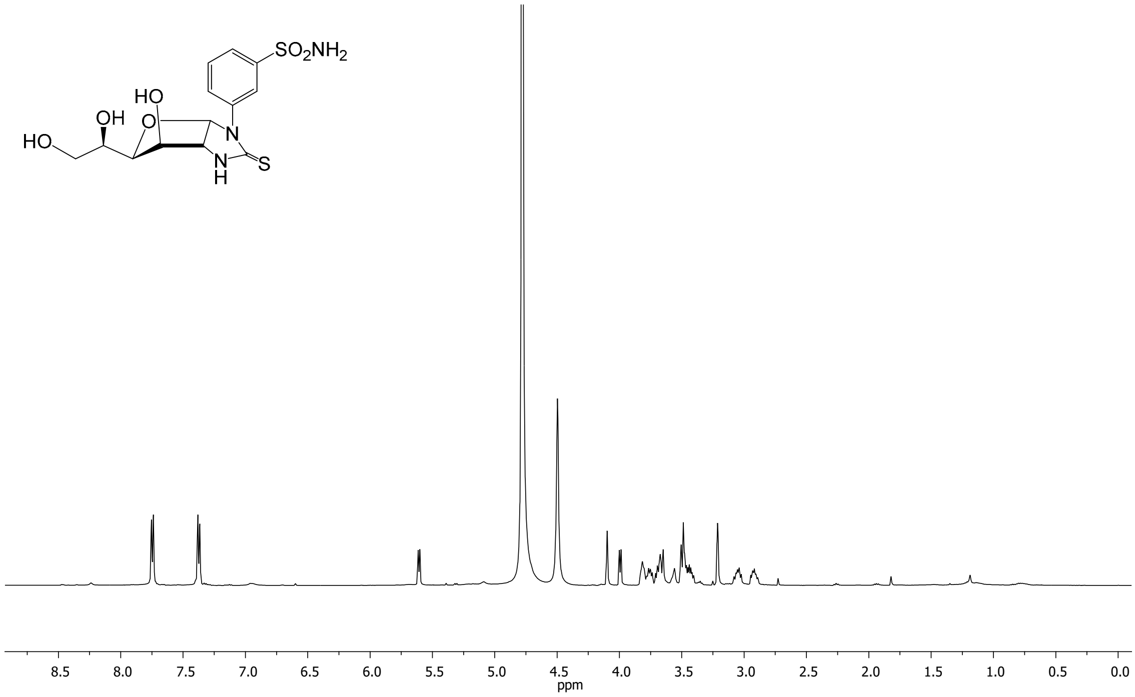

Figure S3. ^{1}H-NMR spectrum of **8b** (300 MHz, CD_3OD)

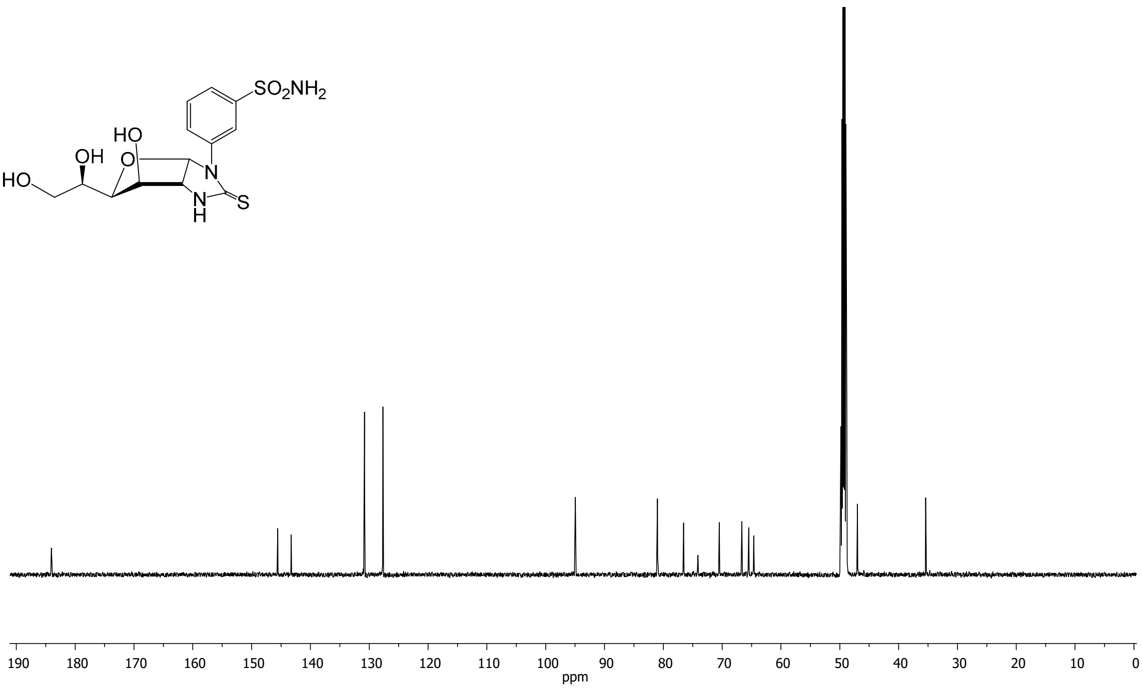

Figure S4. ^{13}C-NMR spectrum of **8b** (75.5 MHz, CD_3OD)

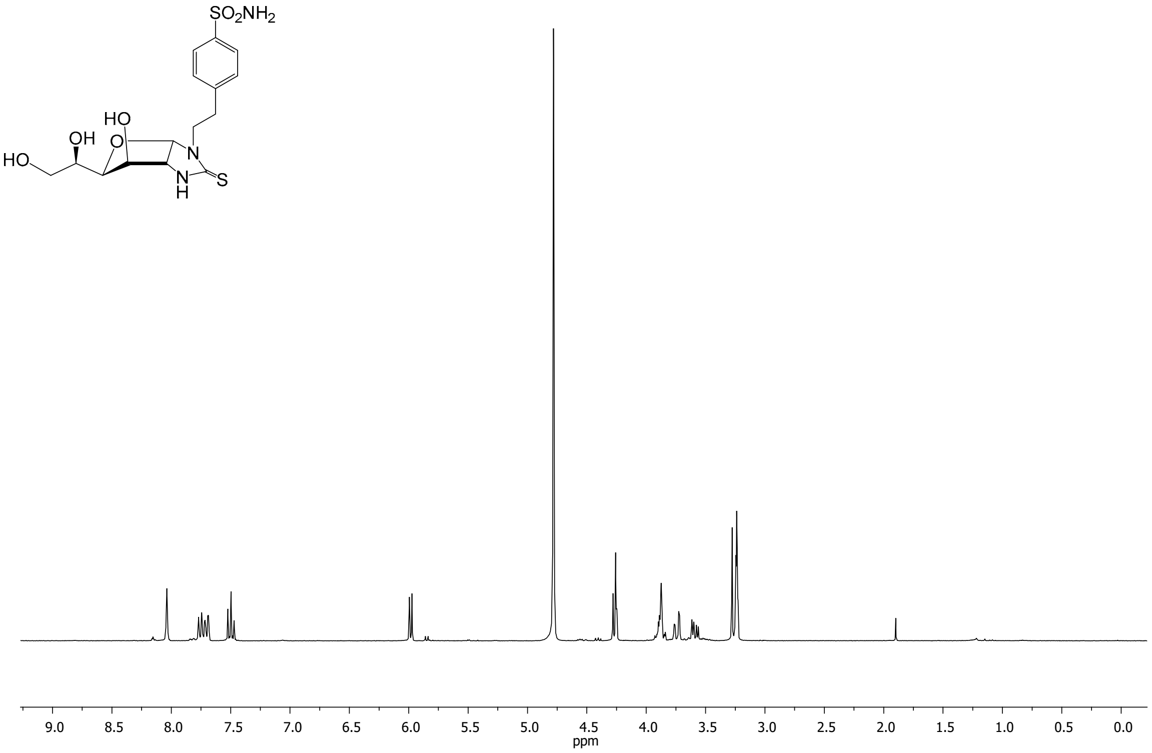

Figure S5. ^{1}H-NMR spectrum of **8c** (300 MHz, CD_3OD)

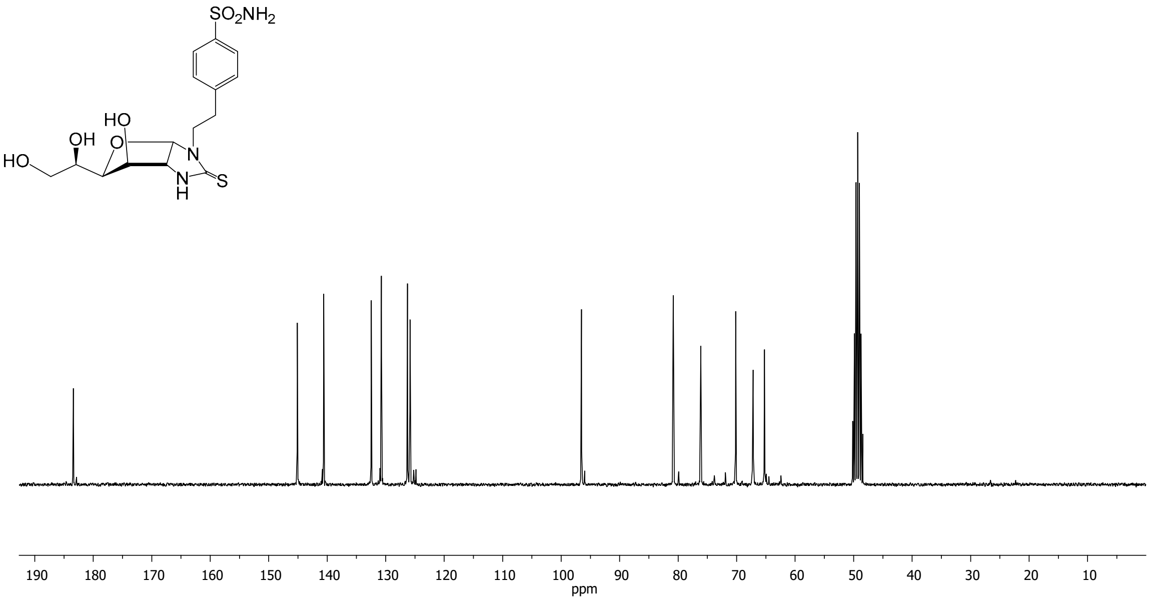

Figure S6. ^{13}C-NMR spectrum of **8c** (75.5 MHz, CD_3OD)

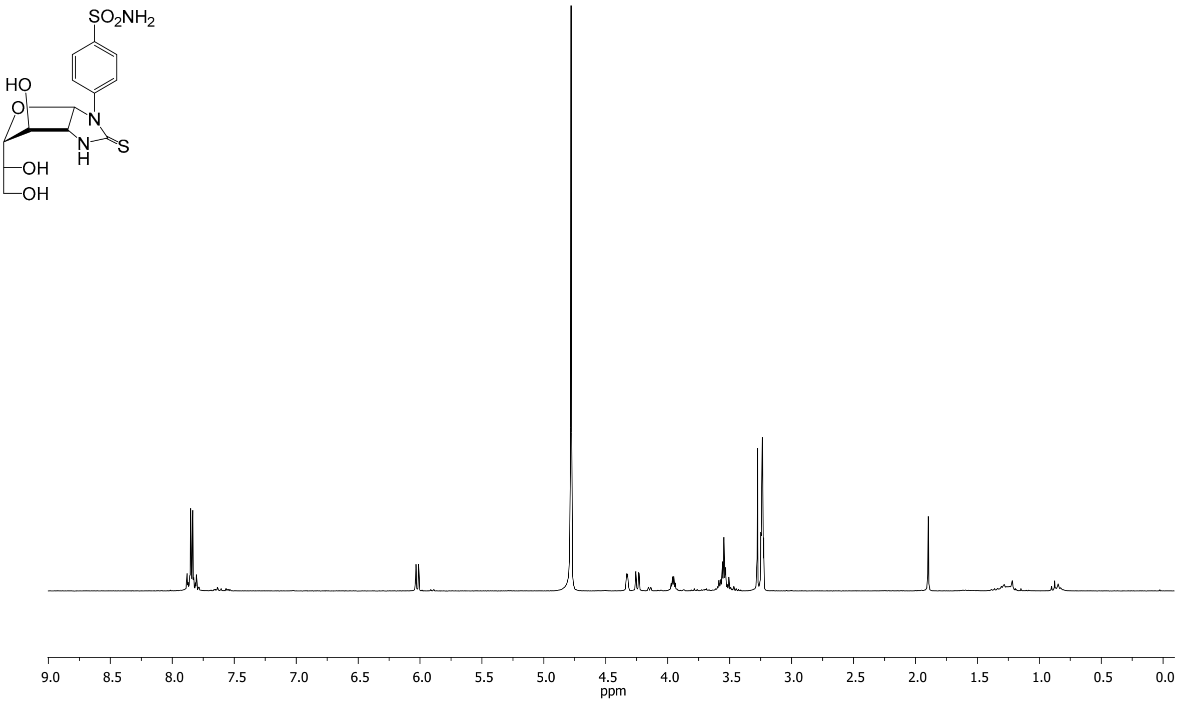

Figure S7. ^{1}H-NMR spectrum of **9a** (300 MHz, CD_3OD)

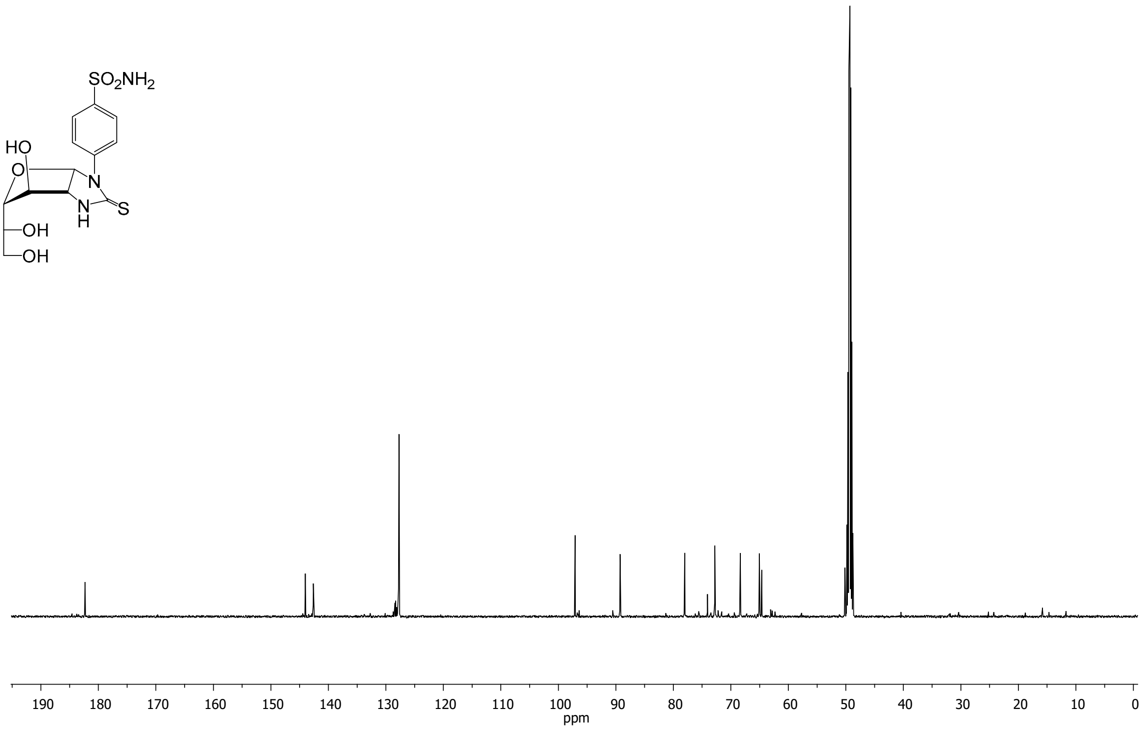

Figure S8. ^{13}C-NMR spectrum of **9a** (125.7 MHz, CD_3OD)

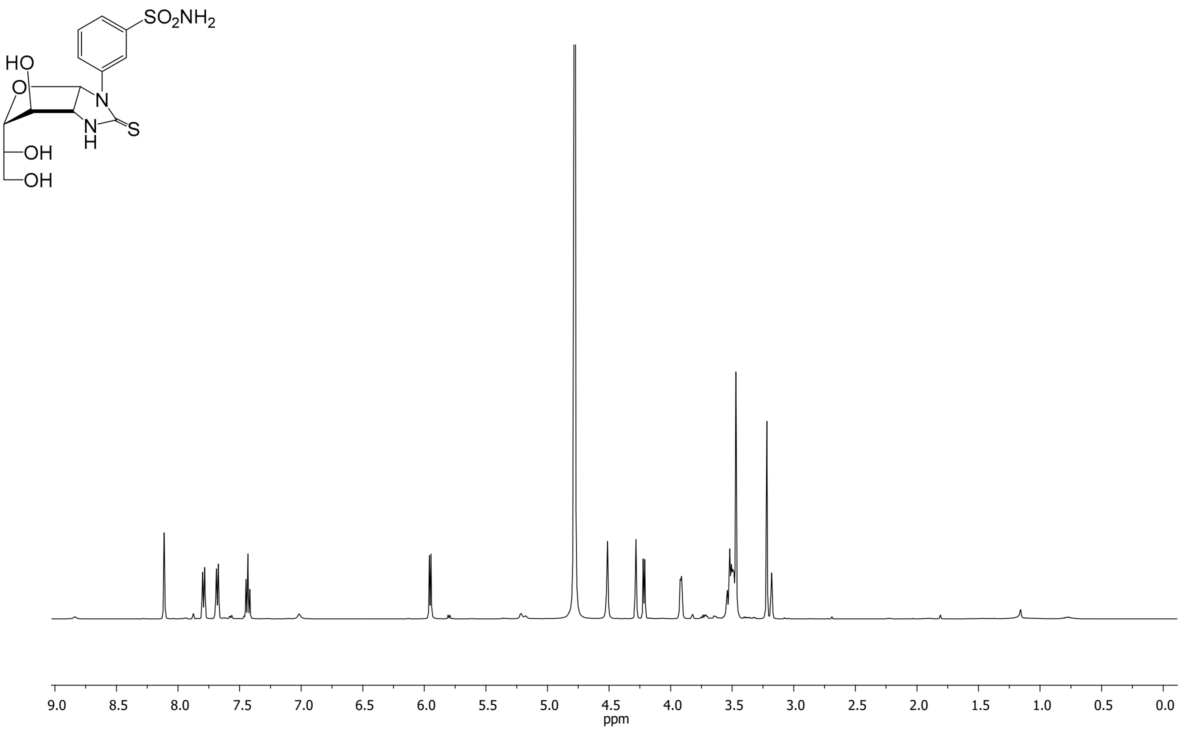

Figure S9. ^{1}H-NMR spectrum of **9b** (300 MHz, CD_3OD)

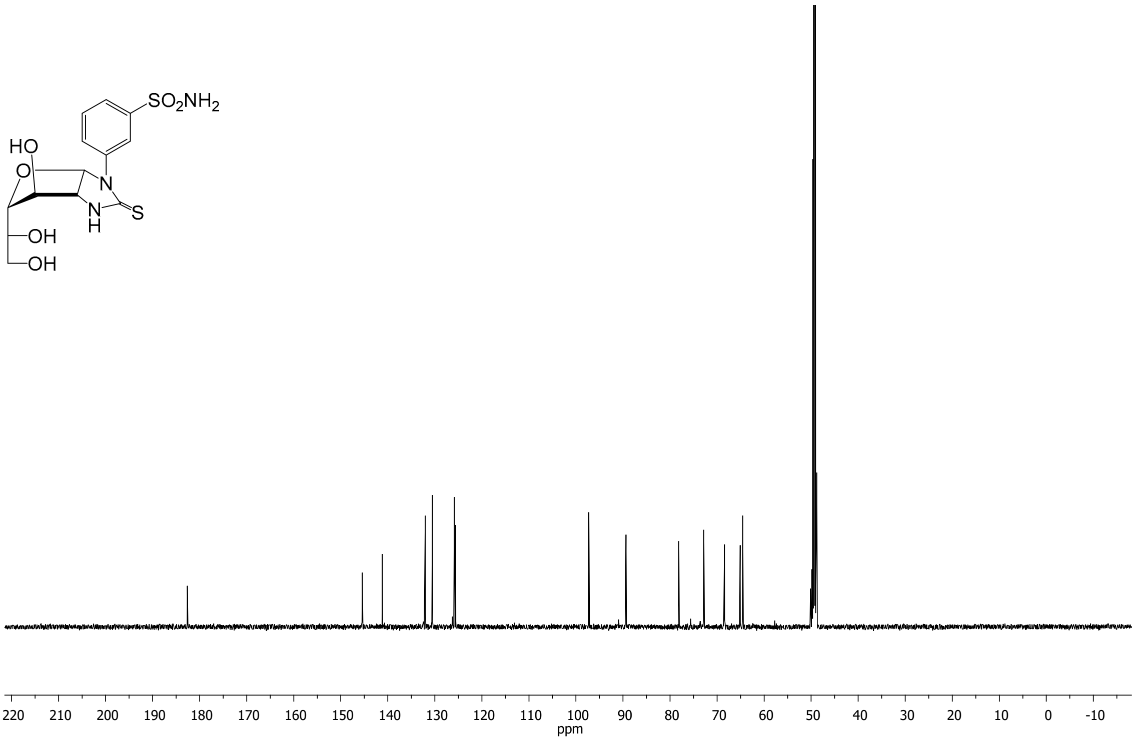

Figure S10. ^{13}C-NMR spectrum of **9b** (125.7 MHz, CD_3OD)

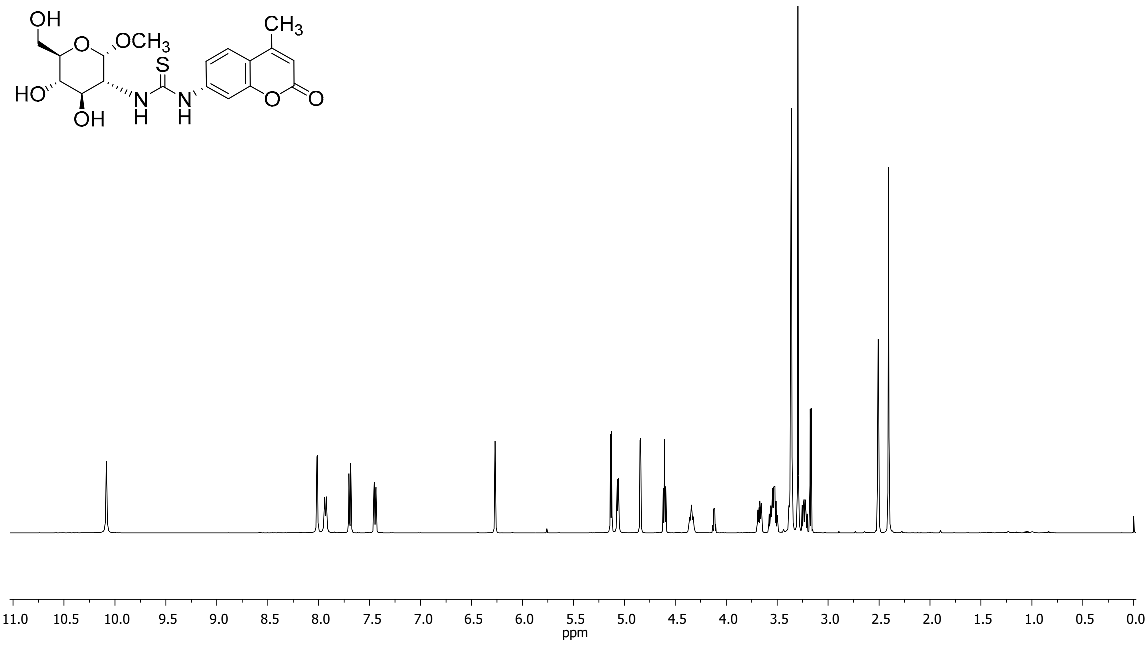

Figure S11. ^{1}H-NMR spectrum of **13** (500 MHz, DMSO-d_6)

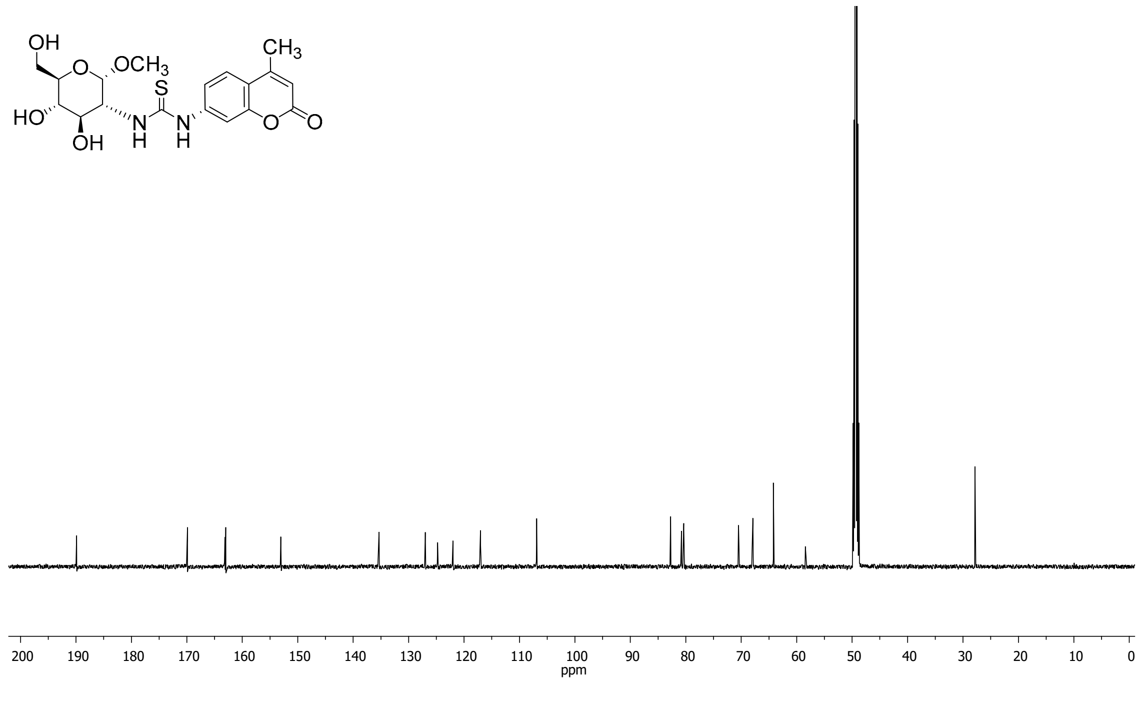

Figure S12. ^{13}C-NMR spectrum of **13** (125.7 MHz, DMSO-d_6)

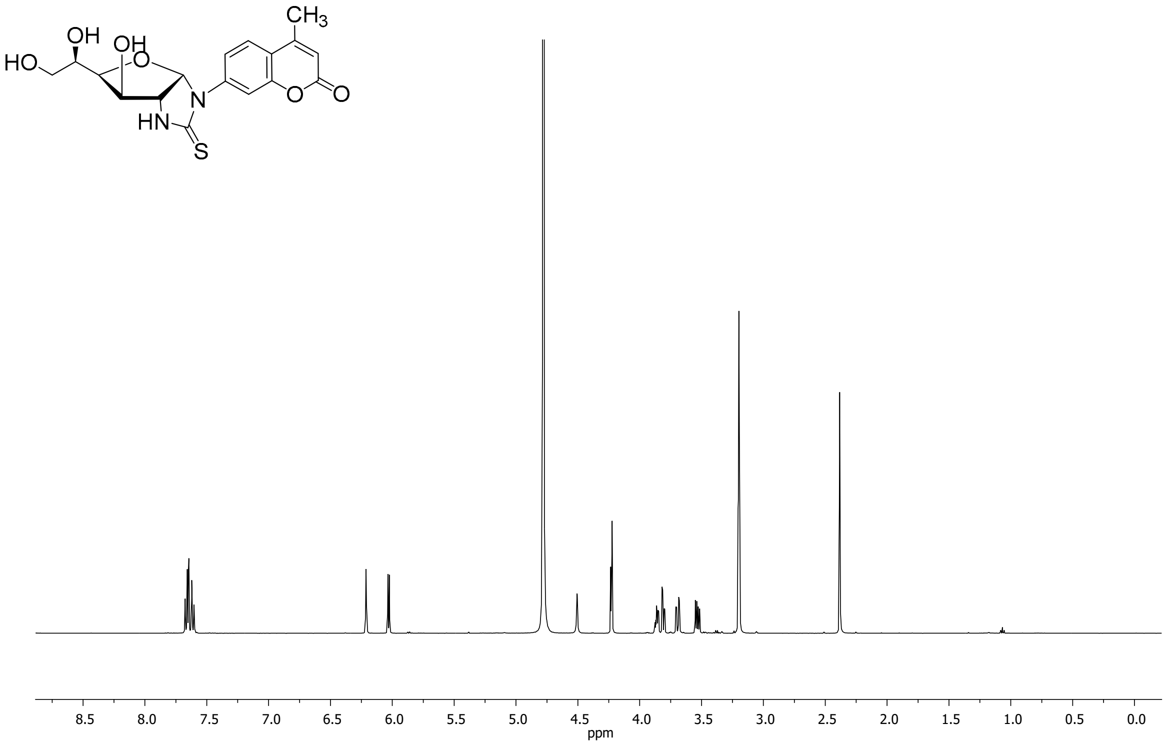

Figure S13. ^{1}H-NMR spectrum of **16** (500 MHz, DMSO-d_6)

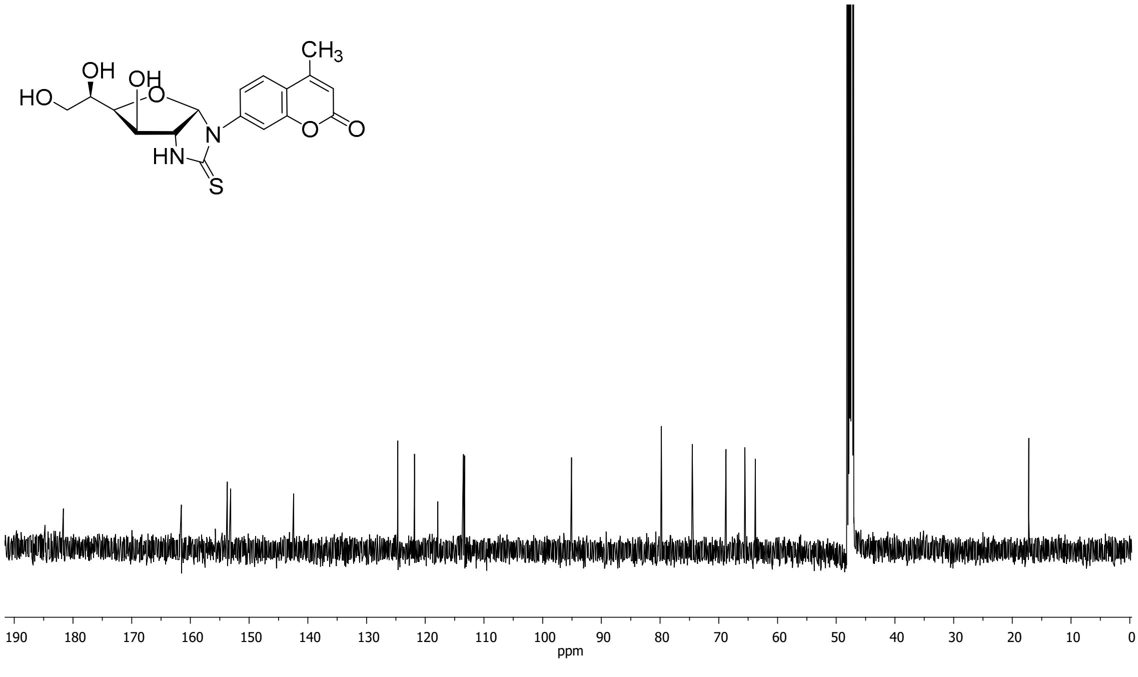

Figure S14. ^{13}C-NMR spectrum of **16** (125.7 MHz, DMSO-d_6)

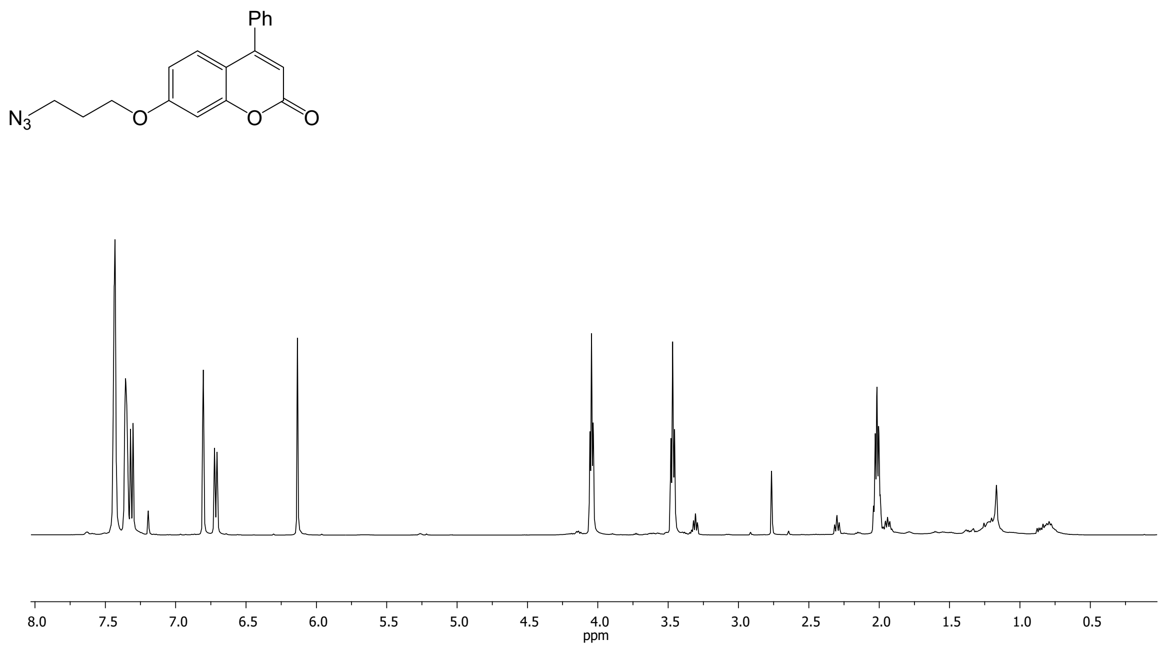

Figure S15. ^{1}H-NMR spectrum of **18d** (500 MHz, $CDCl_3$)

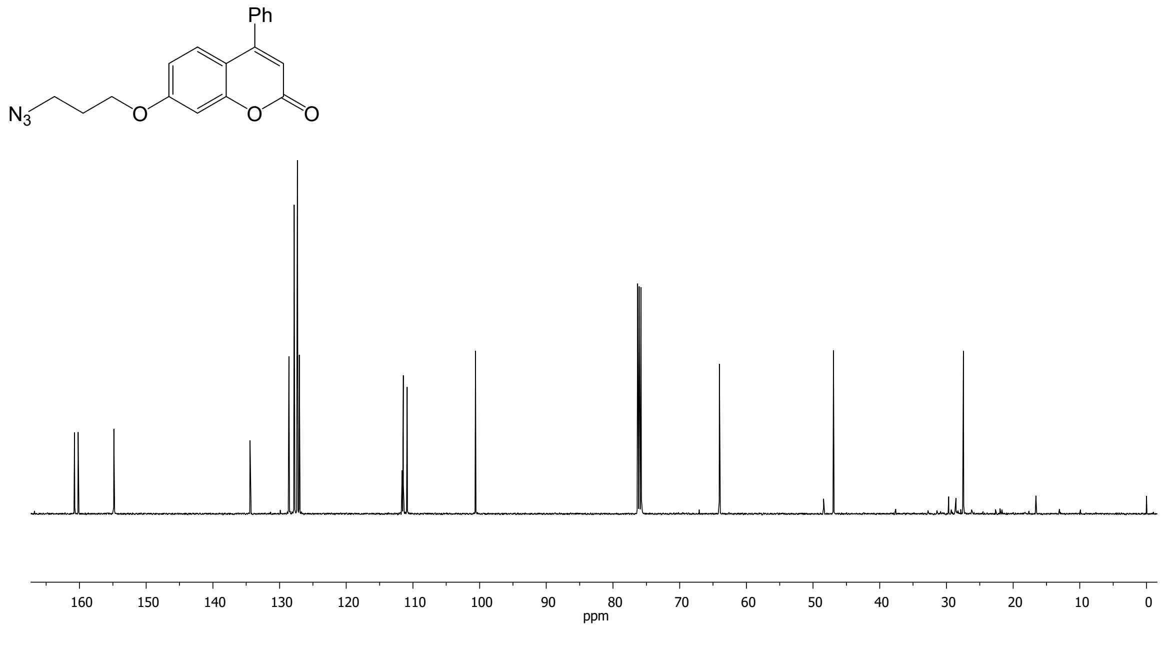

Figure S16. ^{13}C-NMR spectrum of **18d** (125.7 MHz, $CDCl_3$)

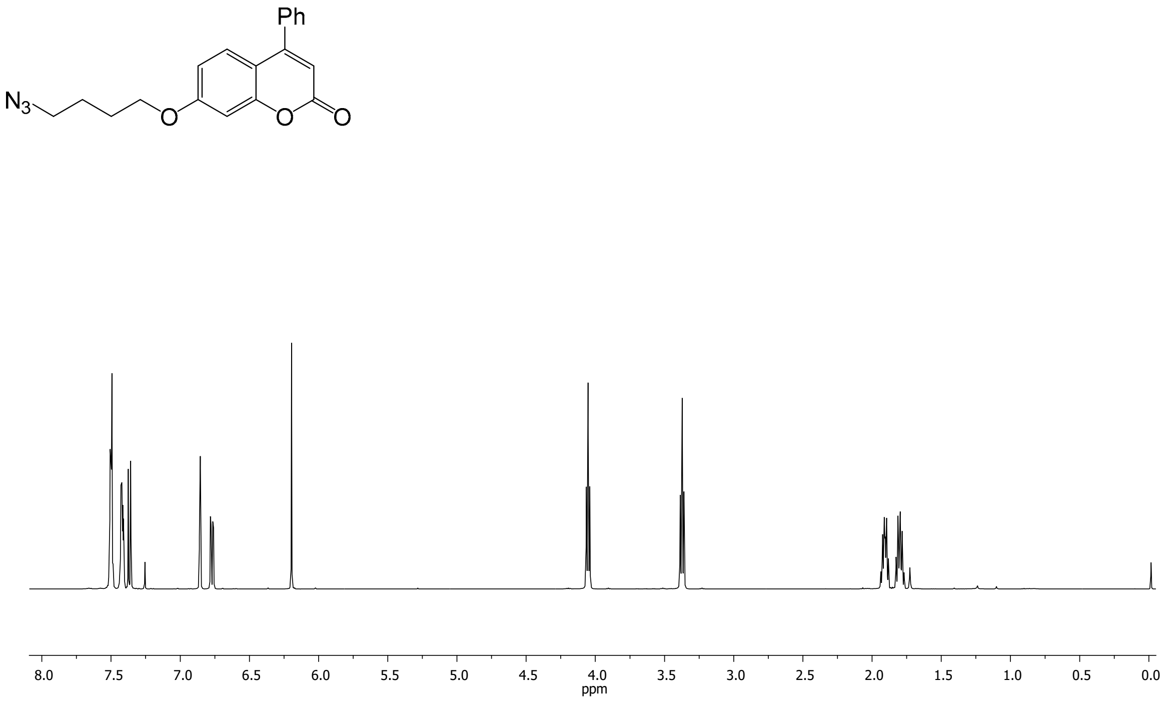

Figure S17. ^{1}H-NMR spectrum of **18e** (500 MHz, $CDCl_3$)

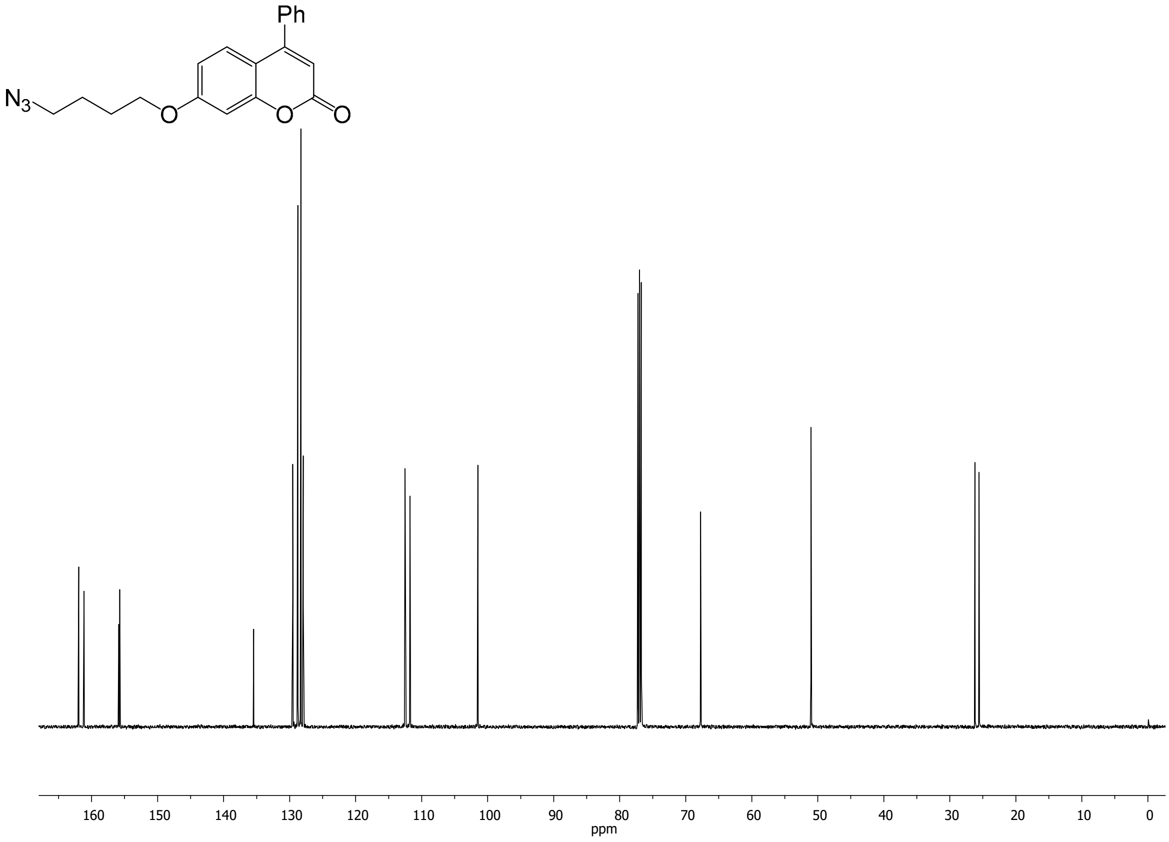

Figure S18. ^{13}C-NMR spectrum of **18e** (127.5 MHz, $CDCl_3$)

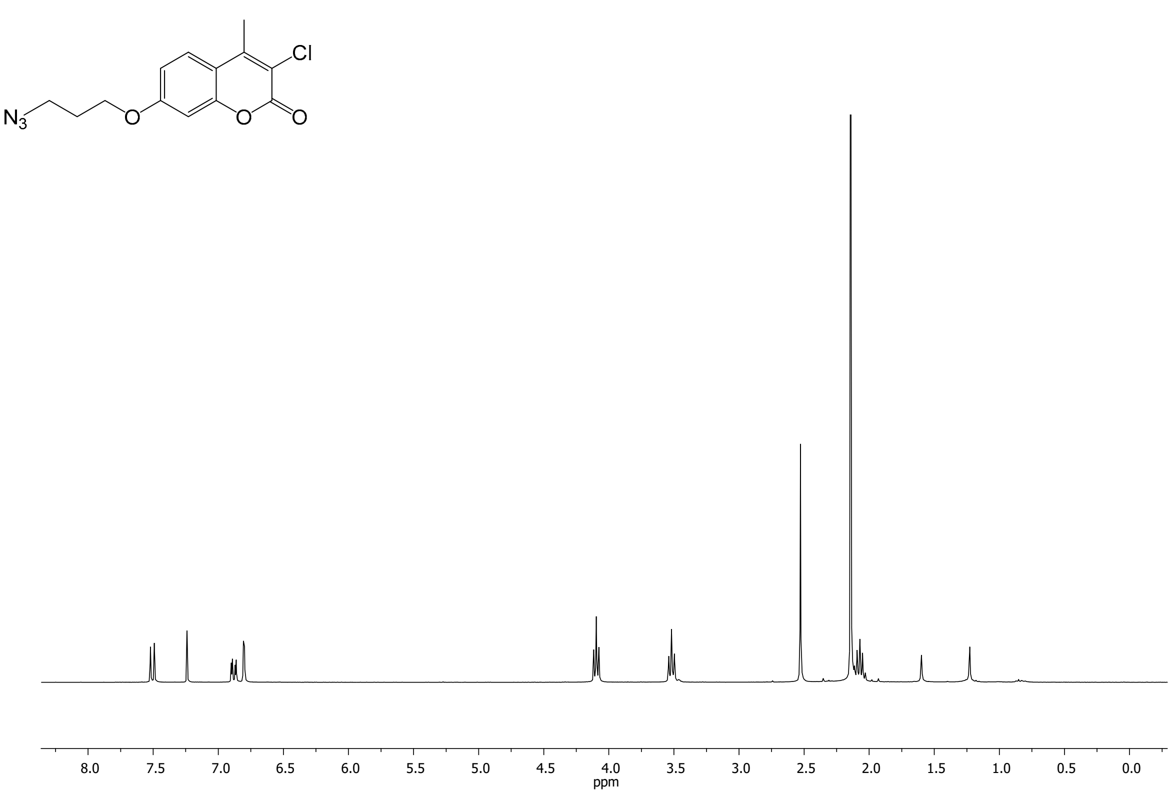

Figure S19. ^{1}H-NMR spectrum of **18g** (300 MHz, $CDCl_3$)

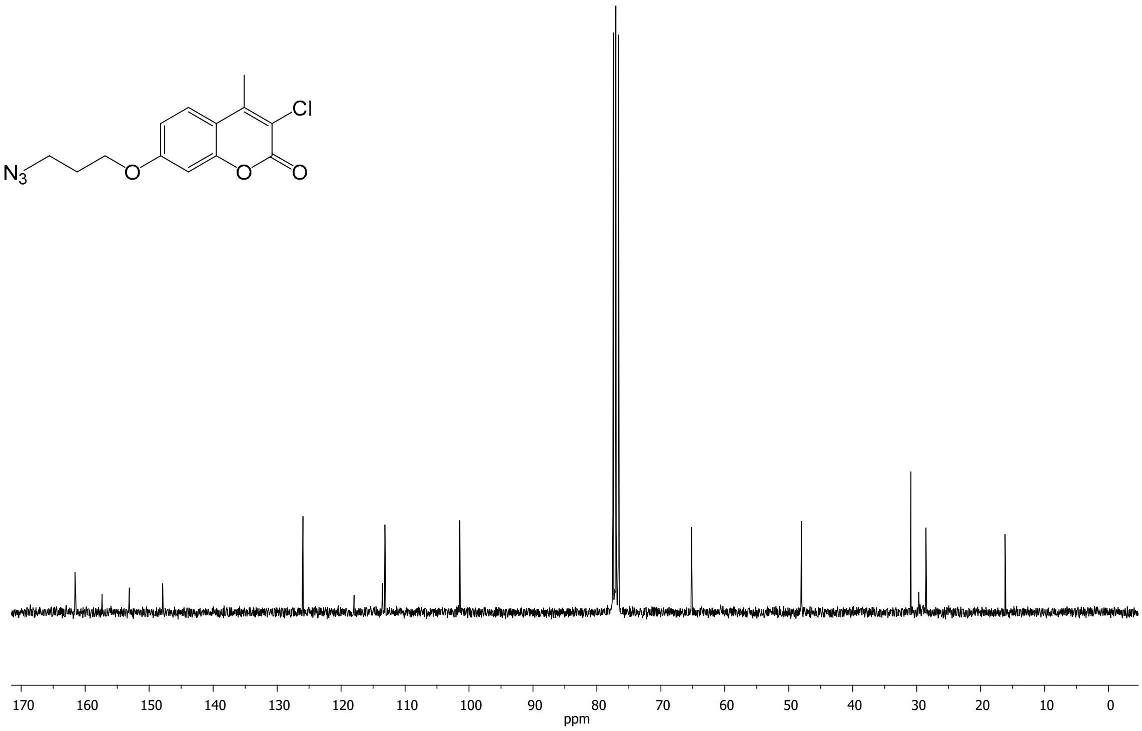

Figure S20. ^{13}C-NMR spectrum of **18g** (125.7 MHz, $CDCl_3$)

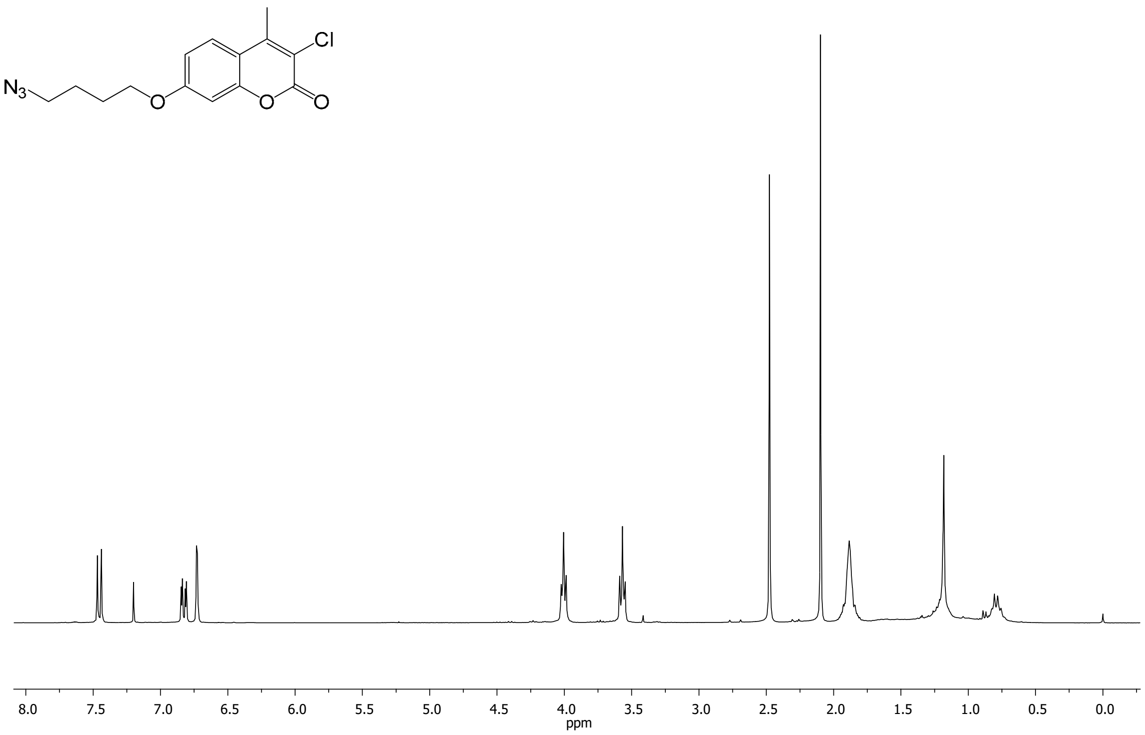

Figure S21. ^{1}H-NMR spectrum of **18h** (500 MHz, $CDCl_3$)

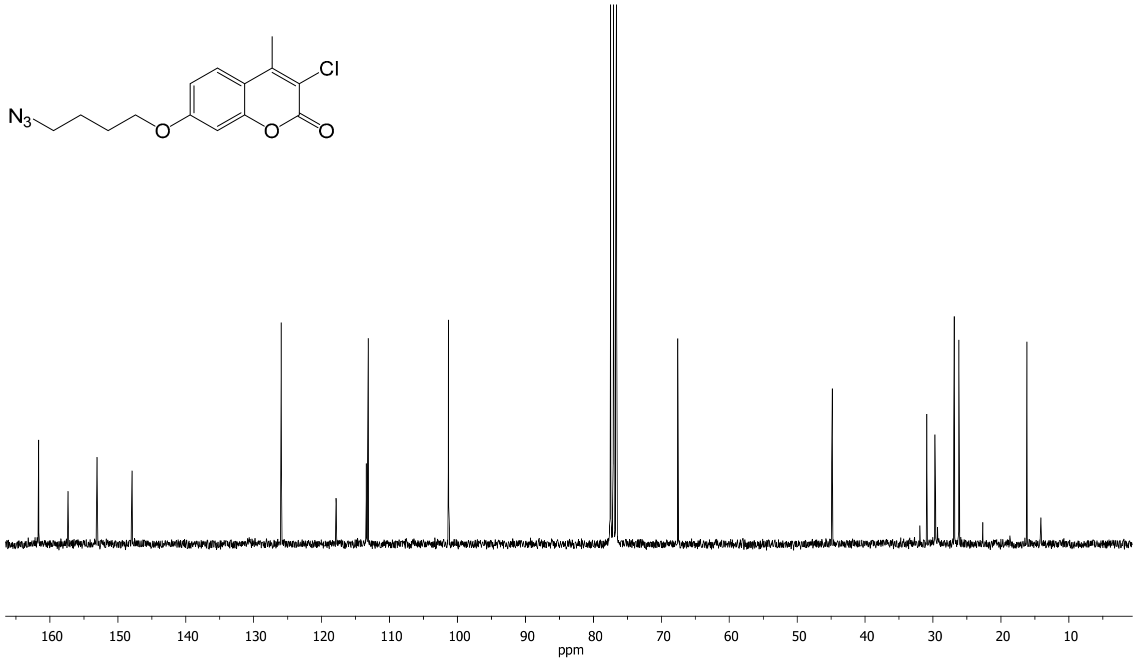

Figure S22. ^{13}C-NMR spectrum of **18h** (125.7 MHz, $CDCl_3$)

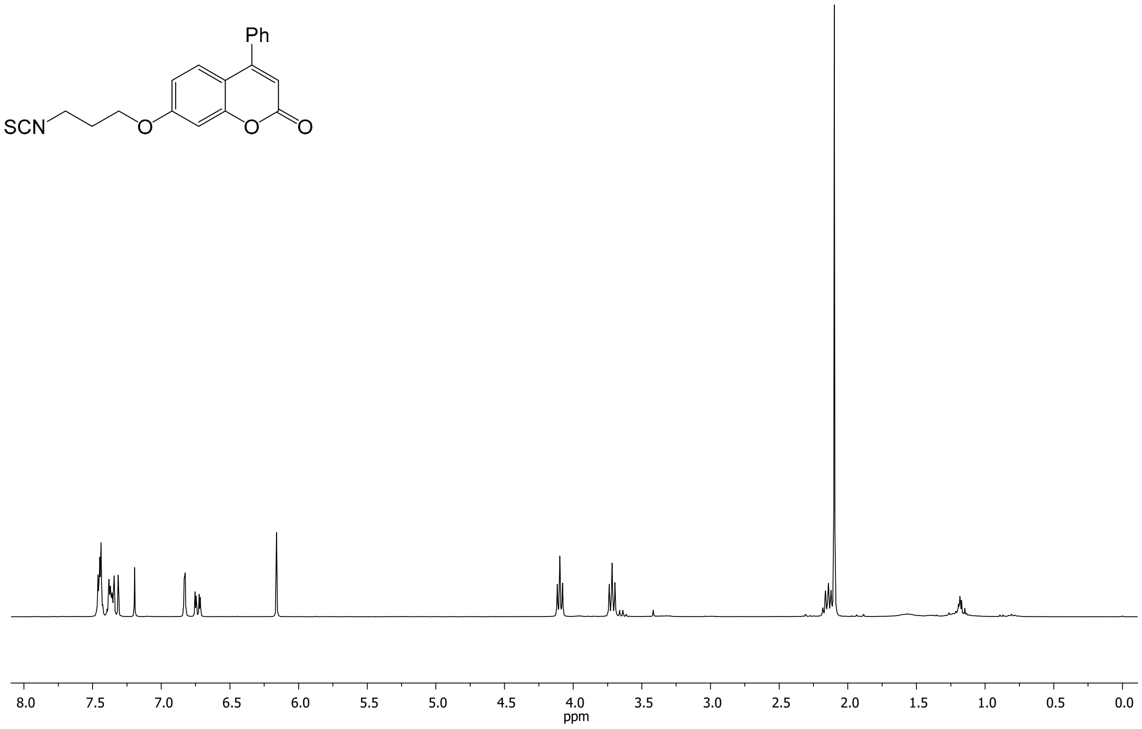

Figure S23. ^{1}H-NMR spectrum of **20d** (300 MHz, $CDCl_3$)

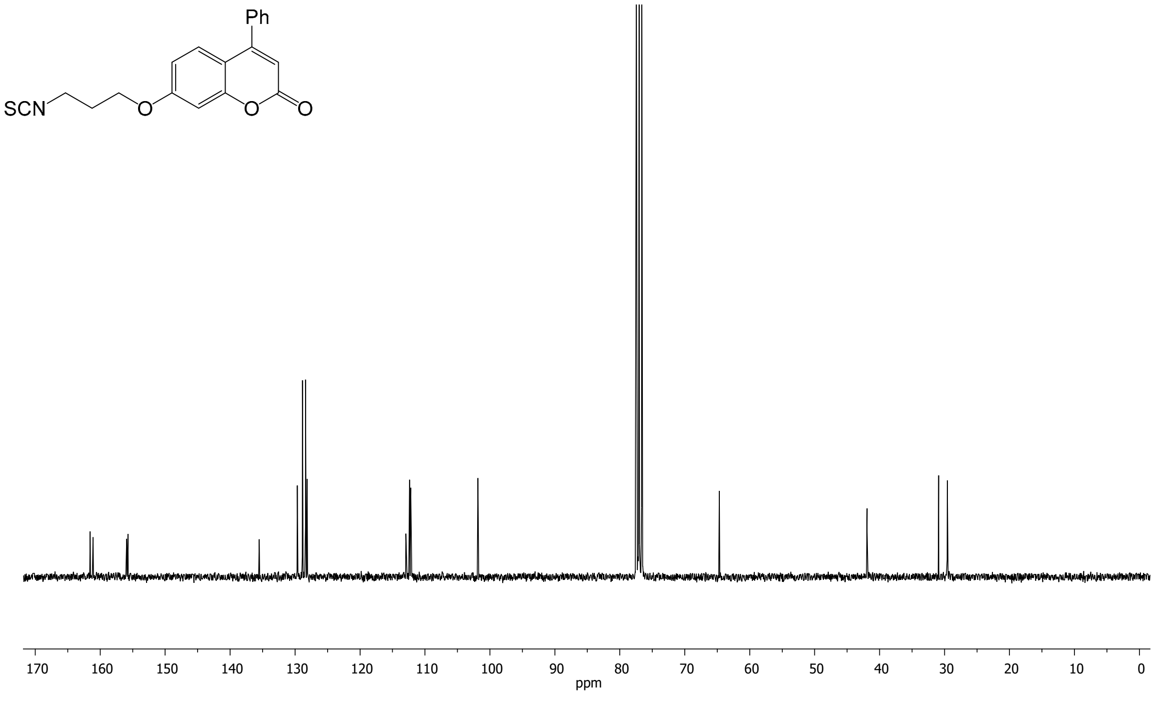

Figure S24. ^{13}C-NMR spectrum of **20d** (125.7 MHz, $CDCl_3$)

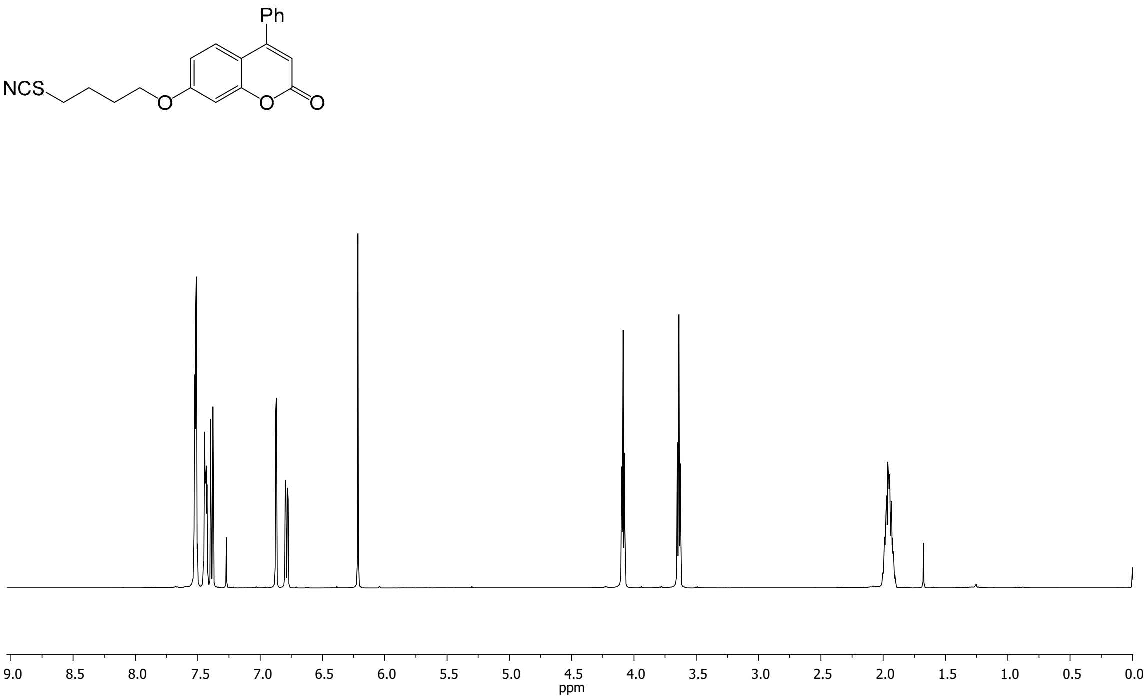

Figure S25. ^{1}H-NMR spectrum of **20e** (500 MHz, $CDCl_3$)

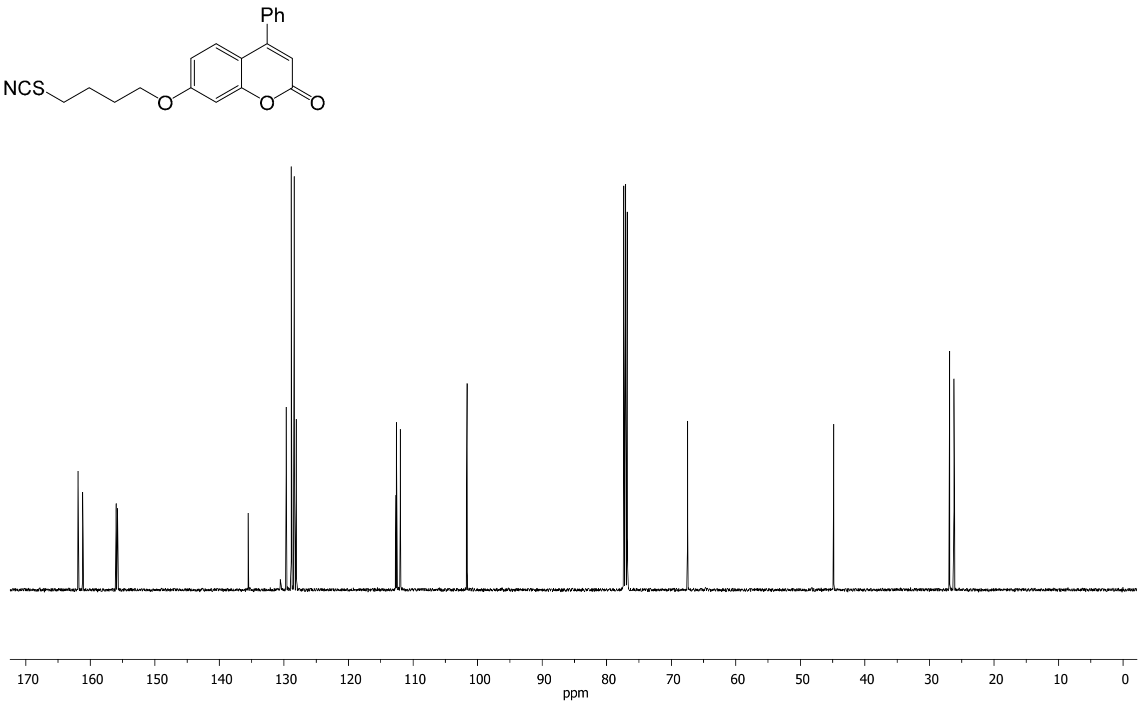

Figure S26. ^{13}C-NMR spectrum of **20e** (125.7 MHz, $CDCl_3$)

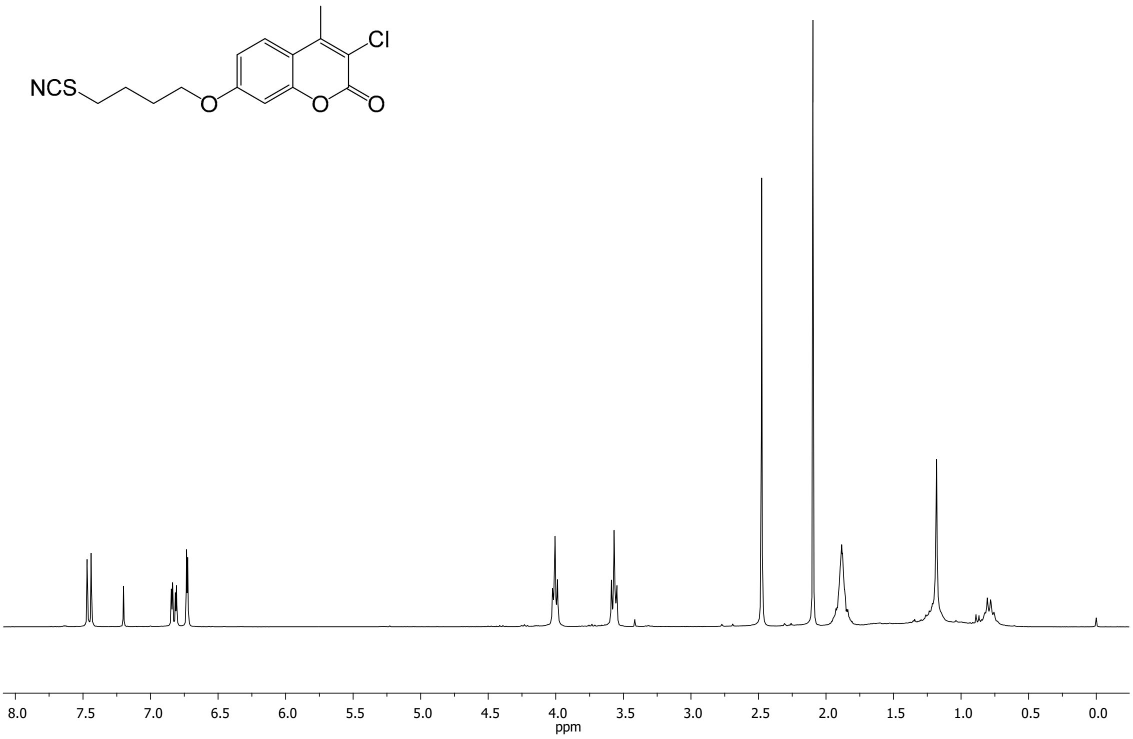

Figure S27. ^{1}H-NMR spectrum of **20h** (300 MHz, $CDCl_3$)

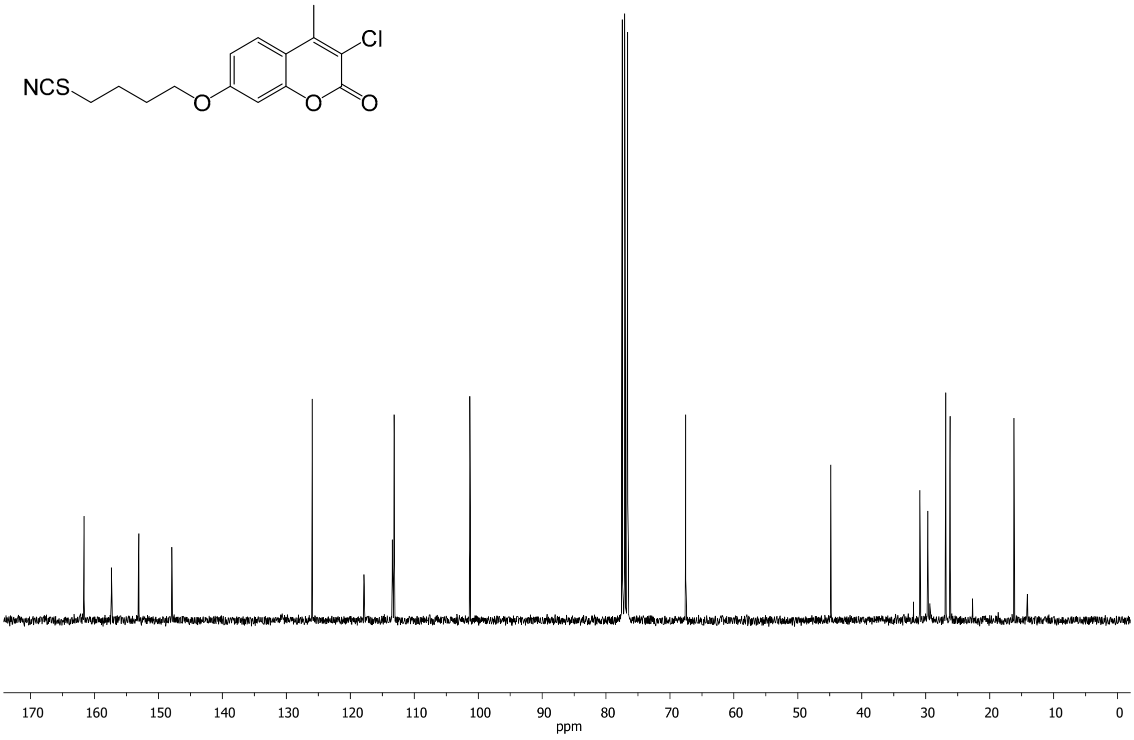

Figure S28. ^{13}C-NMR spectrum of **20h** (125.7 MHz, $CDCl_3$)

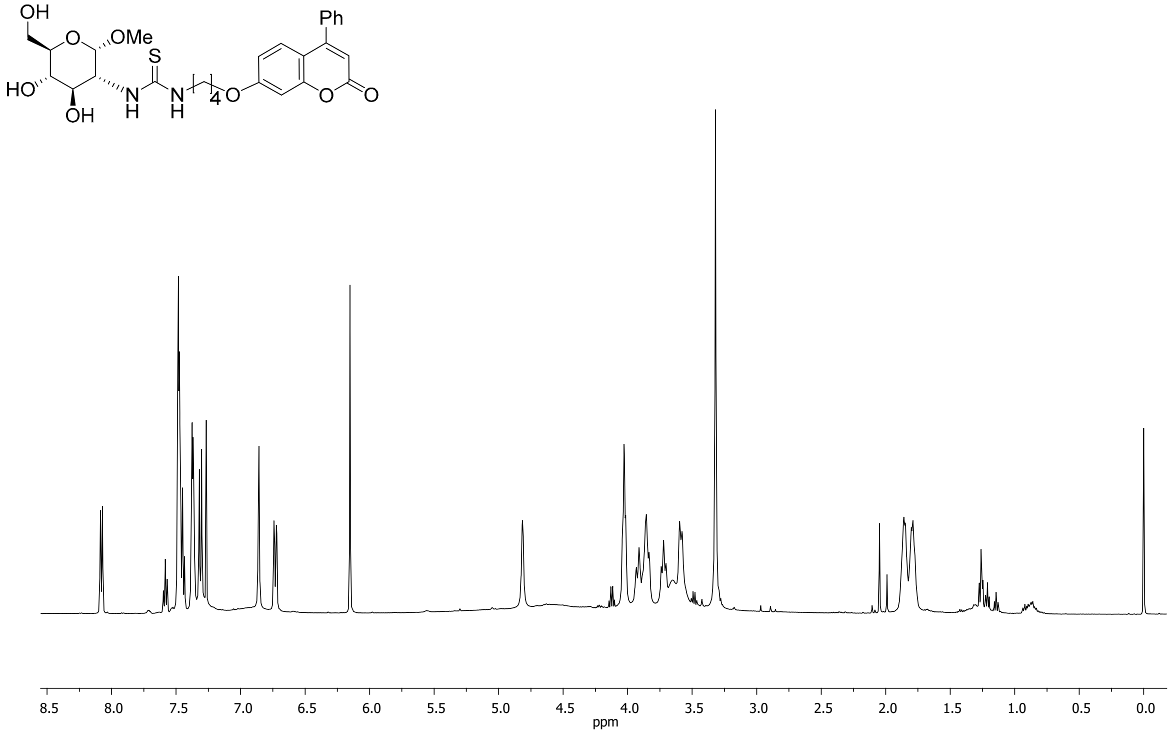

Figure S29. ^{1}H-NMR spectrum of **21e** (300 MHz, DMSO-d_6–$(CD_3)_2CO$)

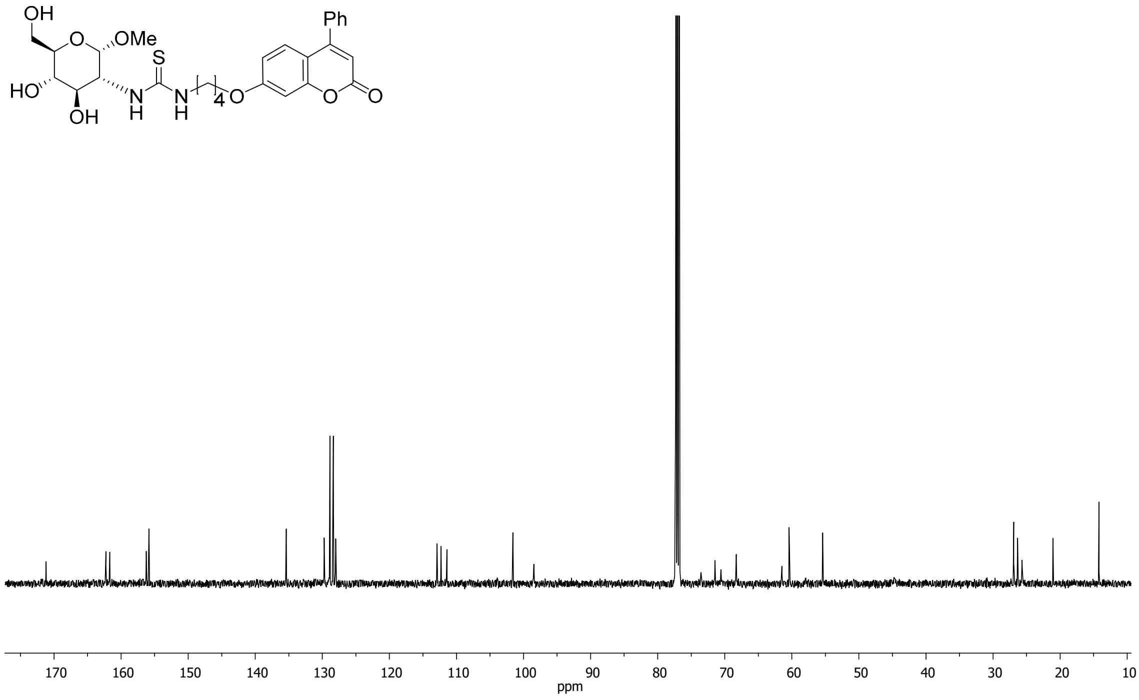

Figure S30. ^{13}C-NMR spectrum of **21e** (75.5 MHz, DMSO-d_6)

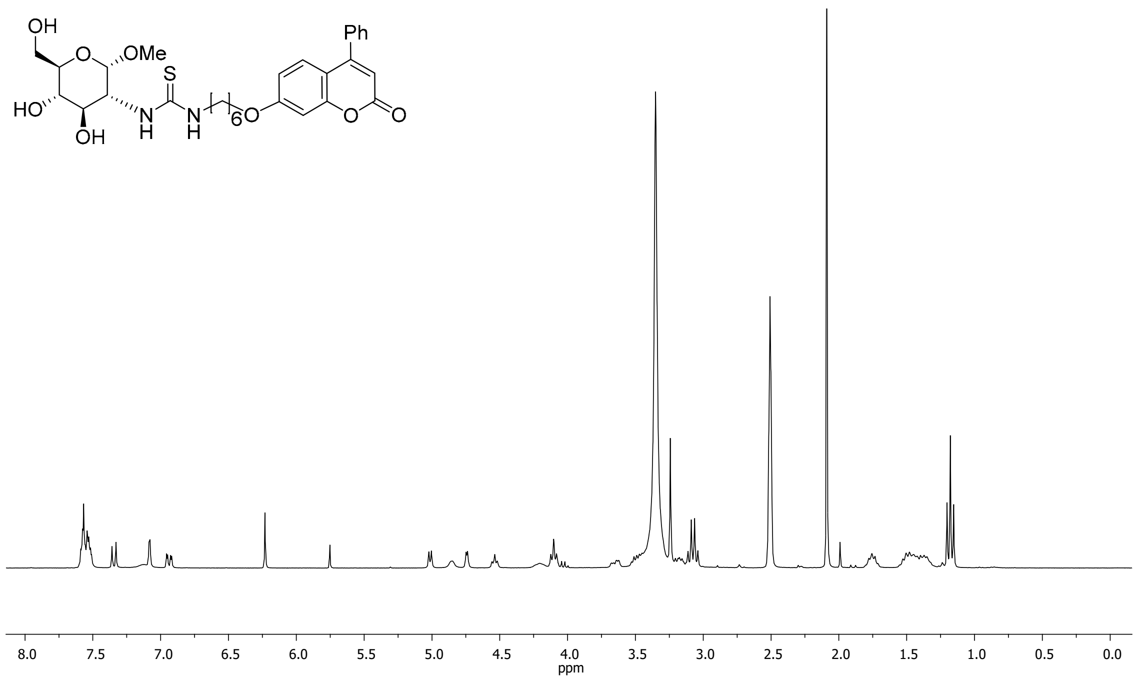

Figure S31. ^{1}H-NMR spectrum of **21f** (300 MHz, DMSO-d_6)

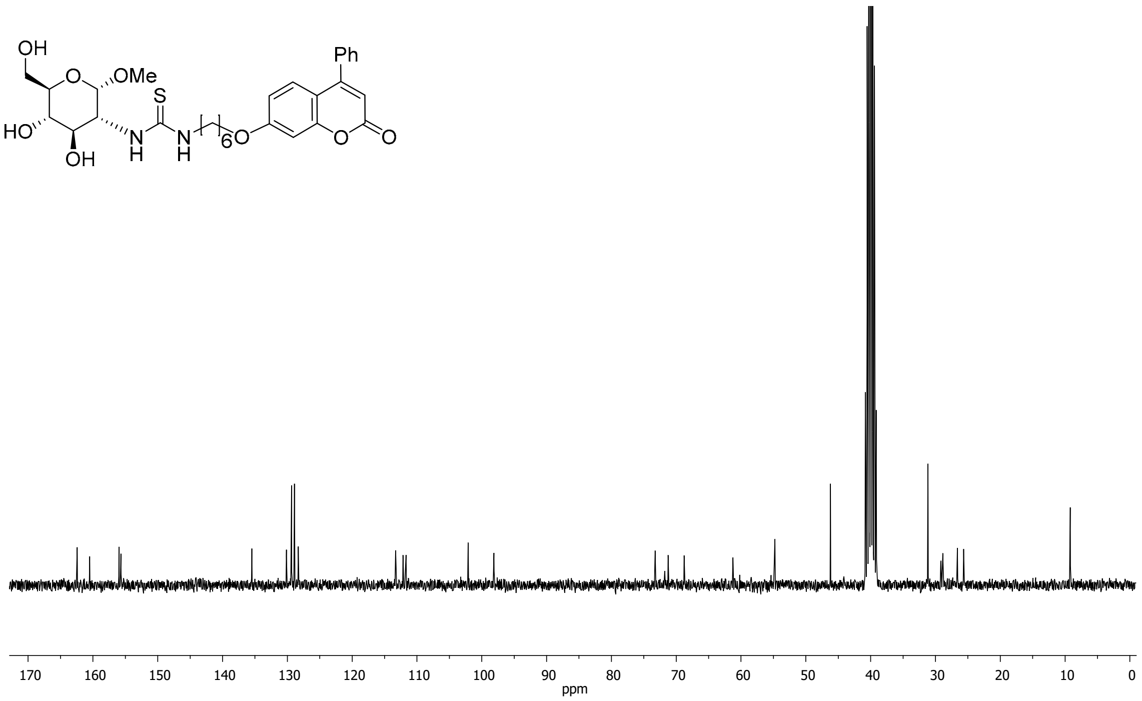

Figure S32. ^{13}C-NMR spectrum of **21f** (75.5 MHz, DMSO-d_6)

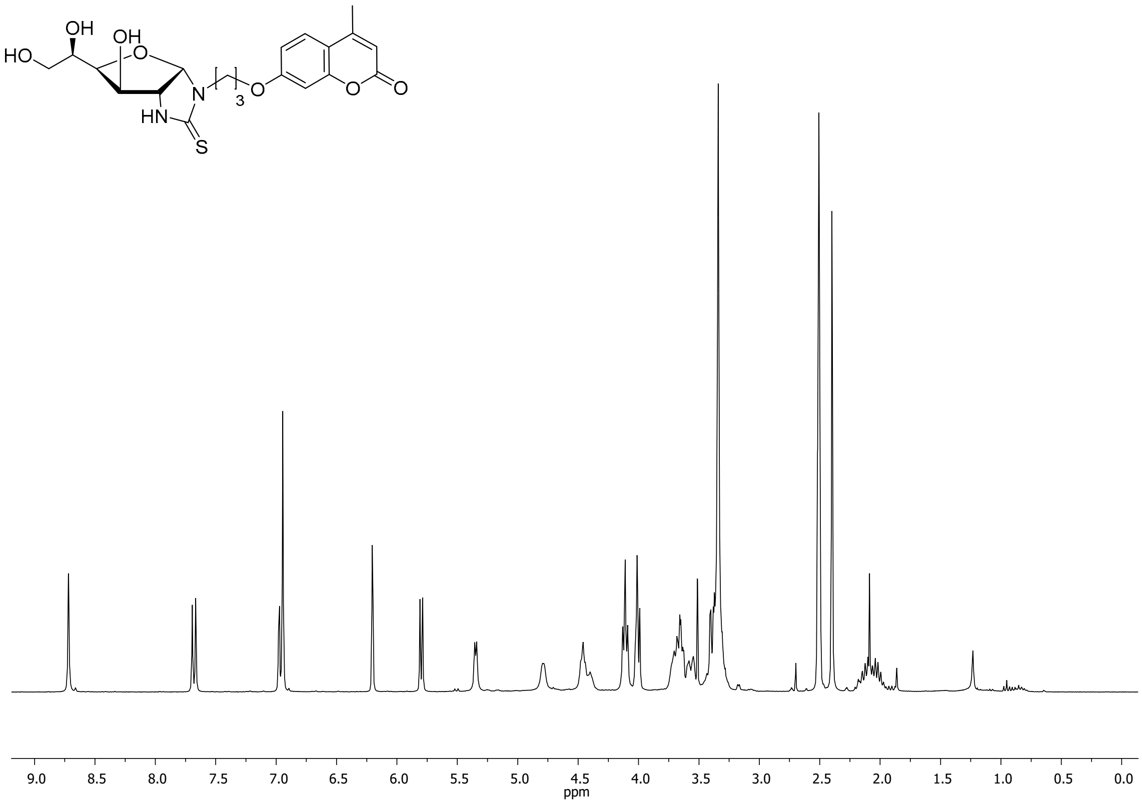

Figure S33. ^{1}H-NMR spectrum of **24a** (300 MHz, DMSO-d_6)

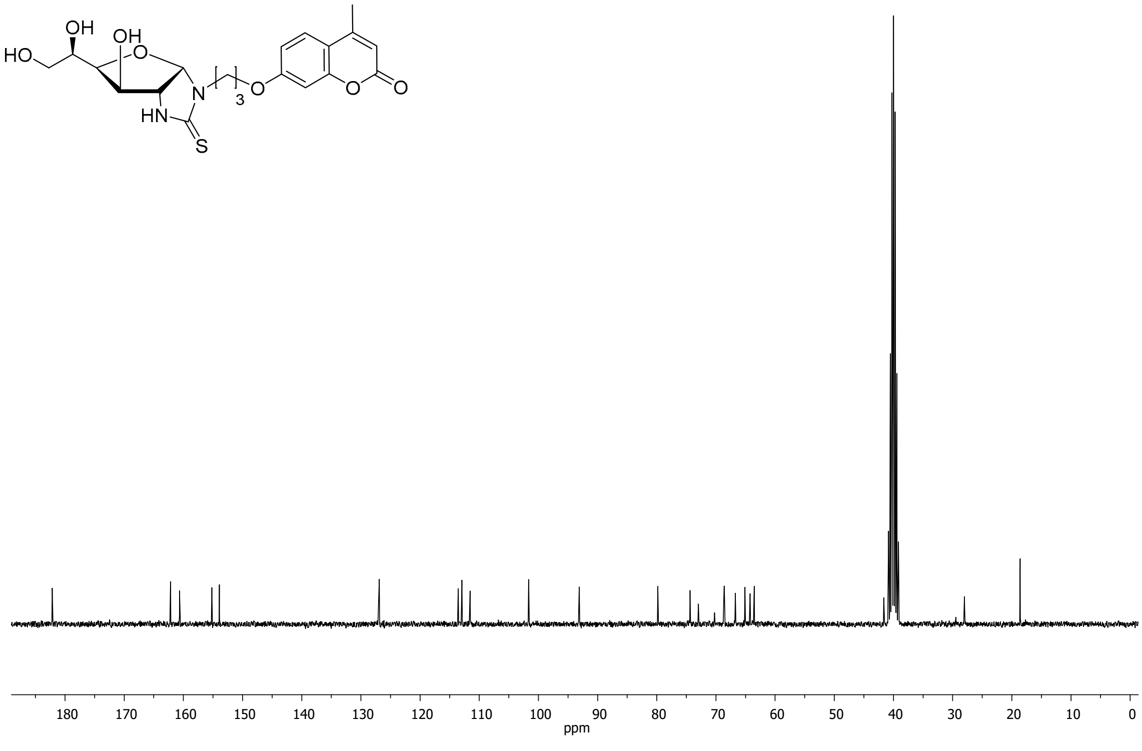

Figure S34. ^{13}C-NMR spectrum of **24a** (75.5 MHz, DMSO-d_6)

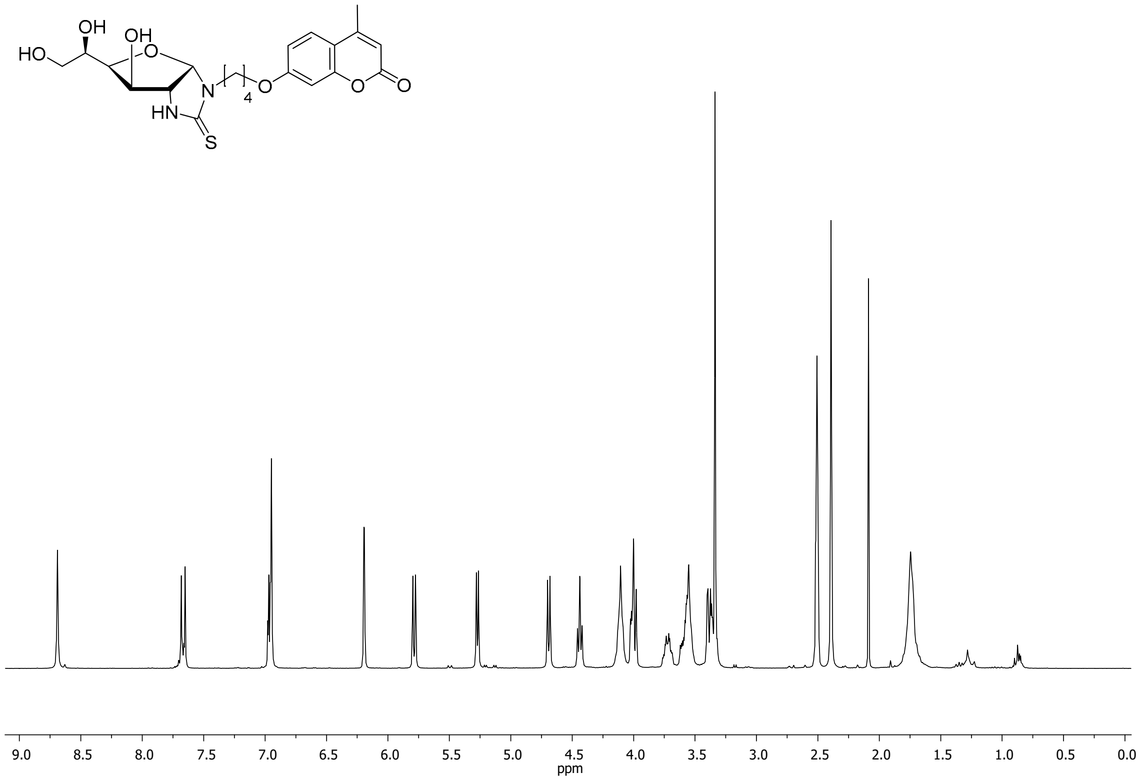

Figure S35. ^{1}H-NMR spectrum of **24b** (300 MHz, DMSO-d_6)

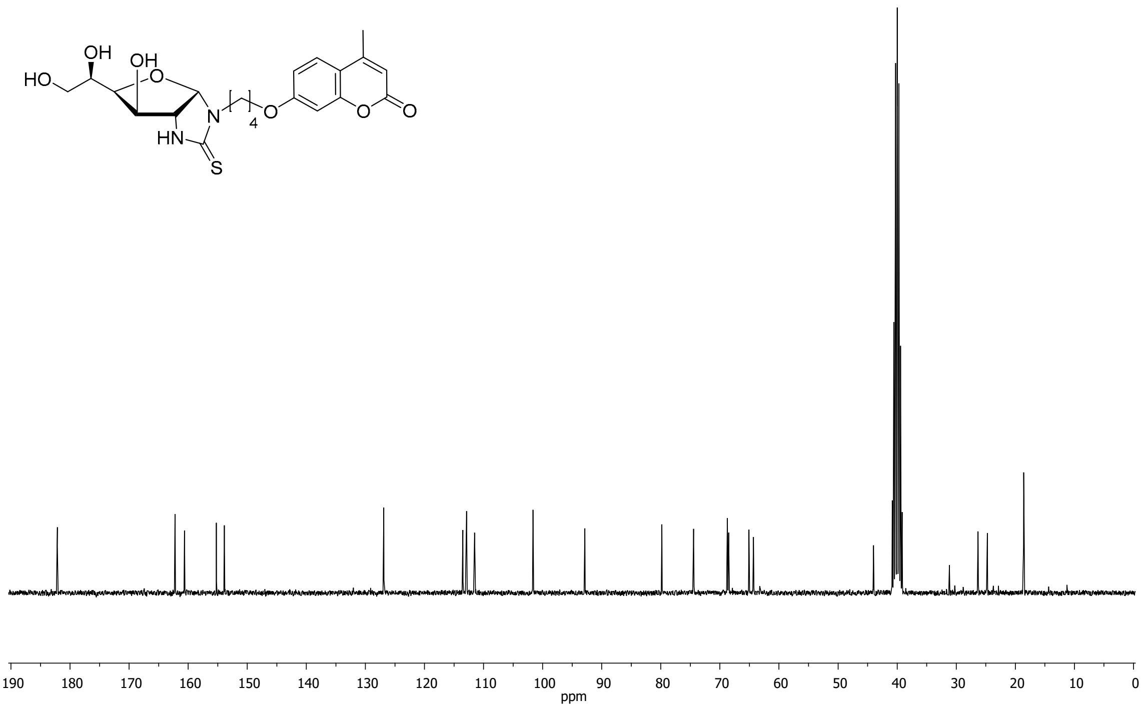

Figure S36. ^{13}C-NMR spectrum of **24b** (75.5 MHz, DMSO-d_6)

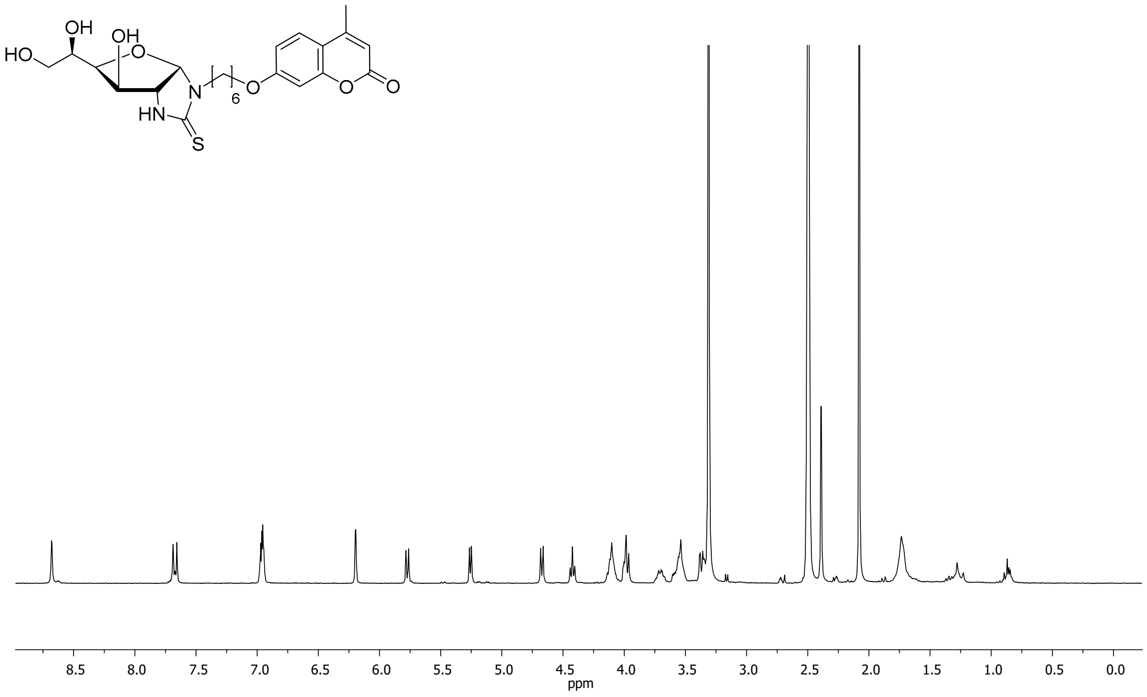

Figure S37. ^{1}H-NMR spectrum of **24c** (300 MHz, DMSO-d_6)

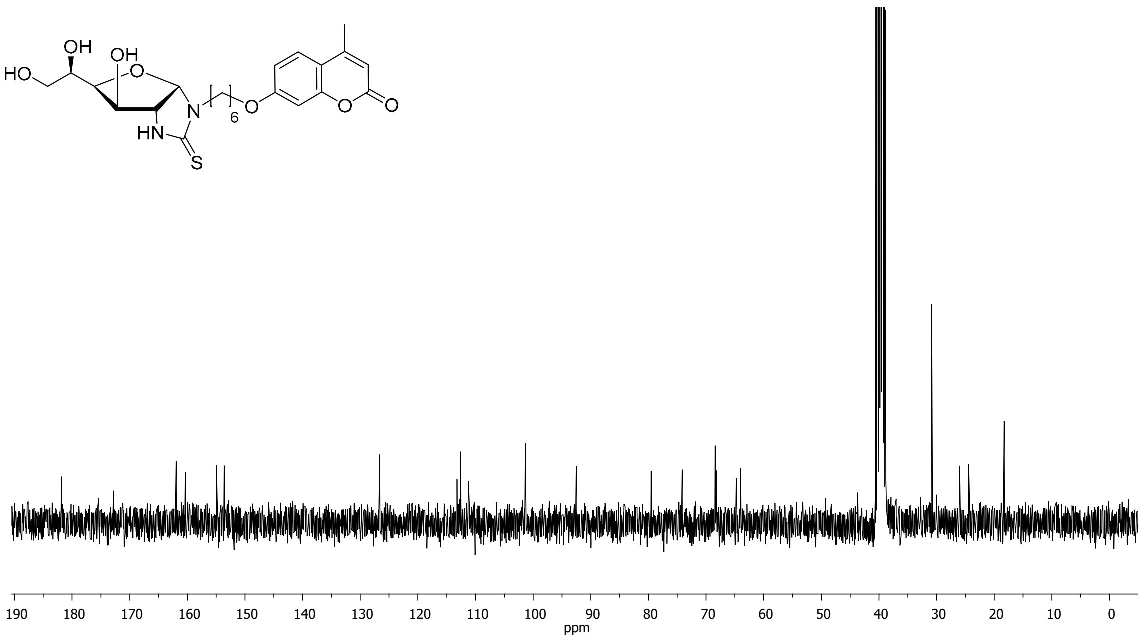

Figure S38. ^{13}C-NMR spectrum of **24c** (75.5 MHz, DMSO-d_6)

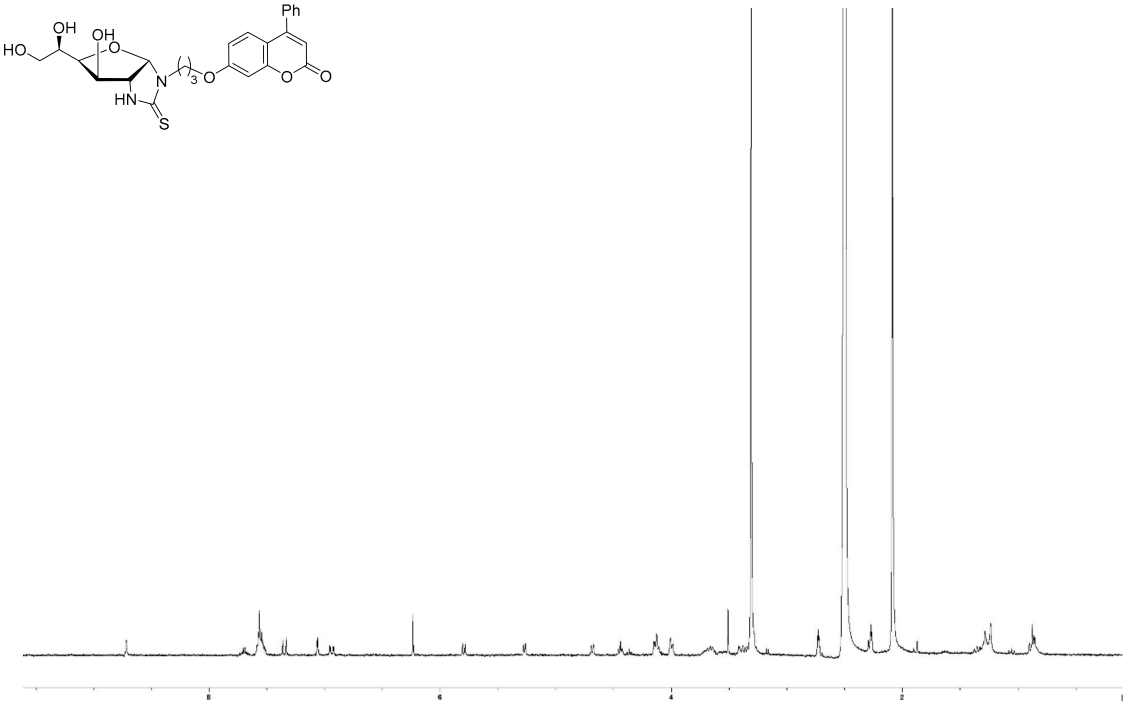

Figure 39. ^{1}H-NMR spectrum of **24d** (300 MHz, DMSO-*d*6)

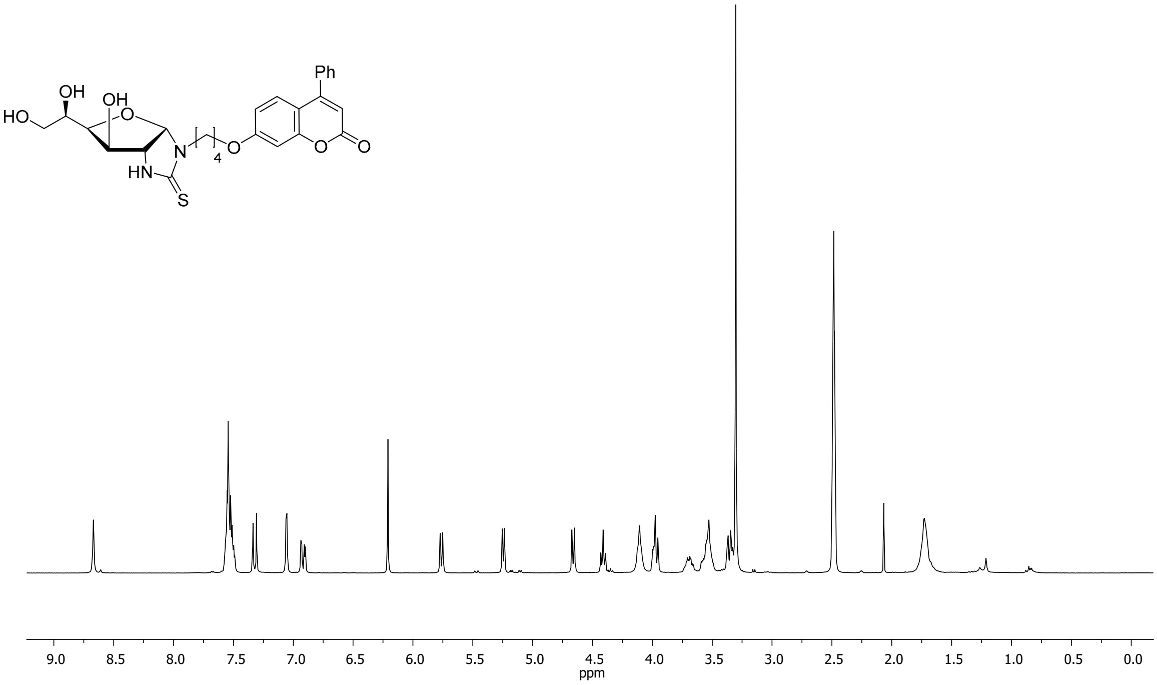

Figure S40. ^{1}H-NMR spectrum of **24e** (300 MHz, DMSO-d_6)

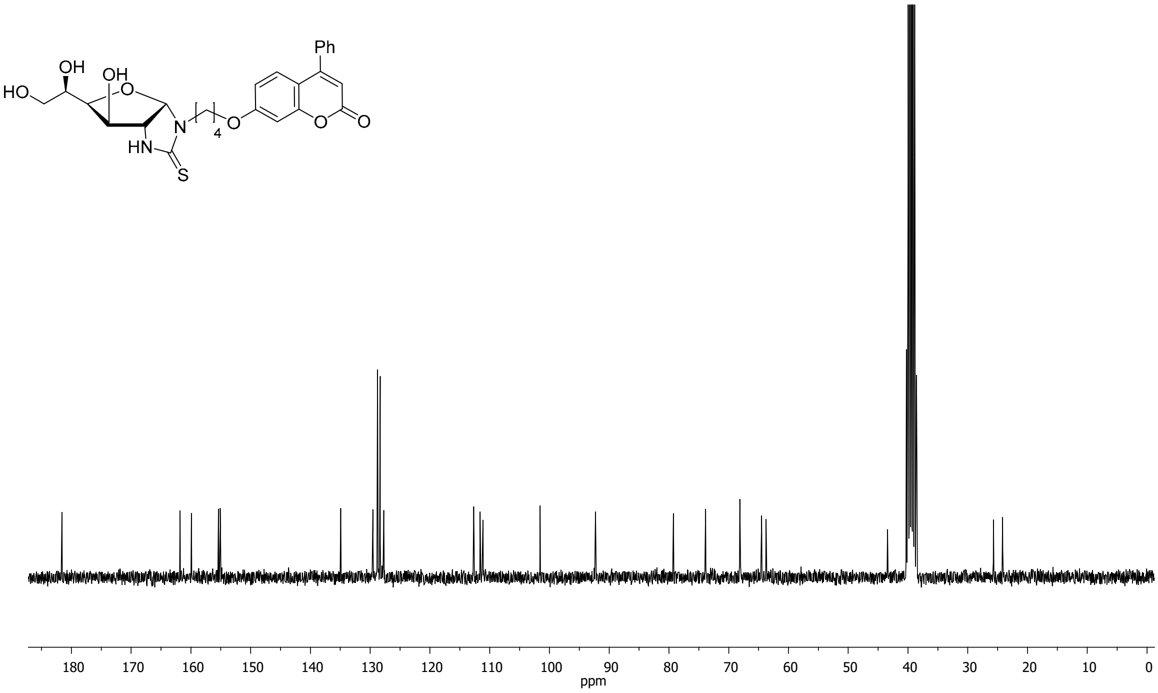

Figure S41. ^{13}C-NMR spectrum of **24e** (75.5 MHz, DMSO-d_6)

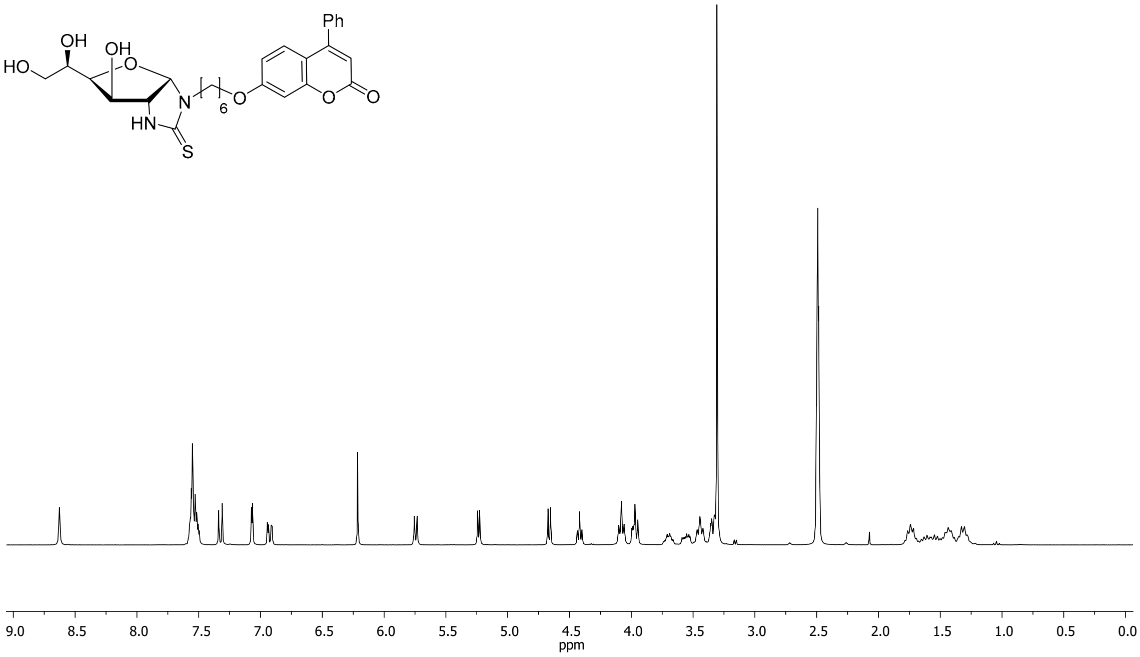

Figure S42. ^{1}H-NMR spectrum of **24f** (300 MHz, DMSO-d_6)

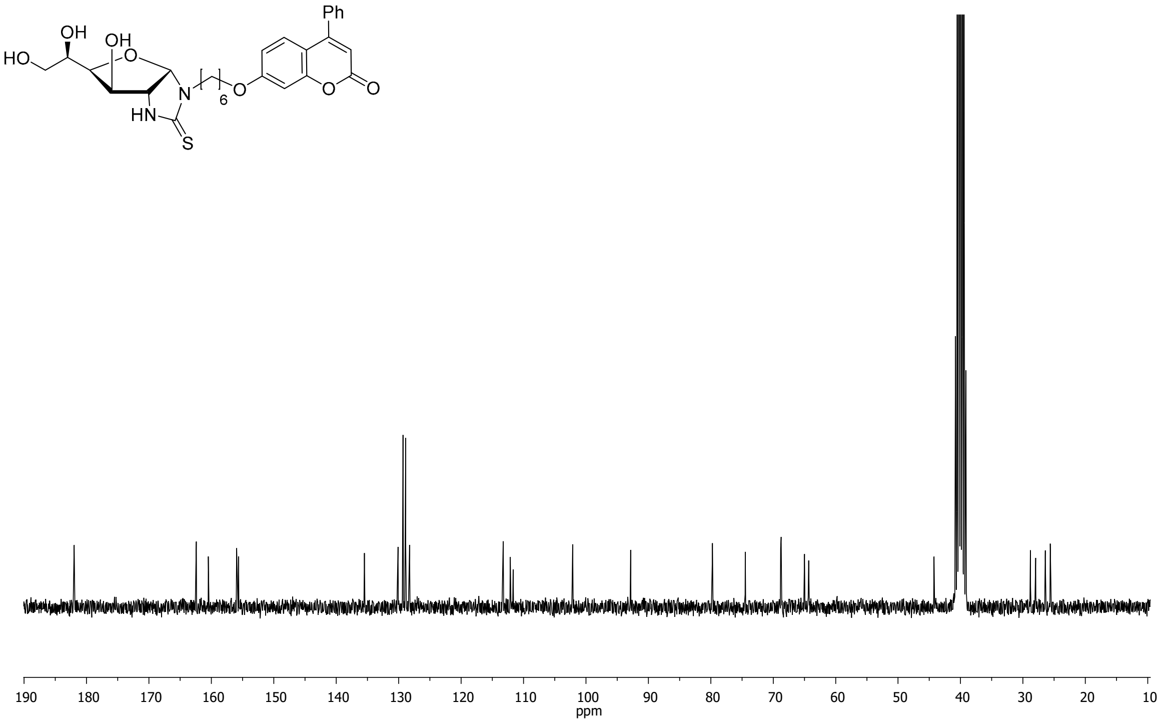

Figure S43. ^{13}C-NMR spectrum of **24f** (75.5 MHz, DMSO-d_6)

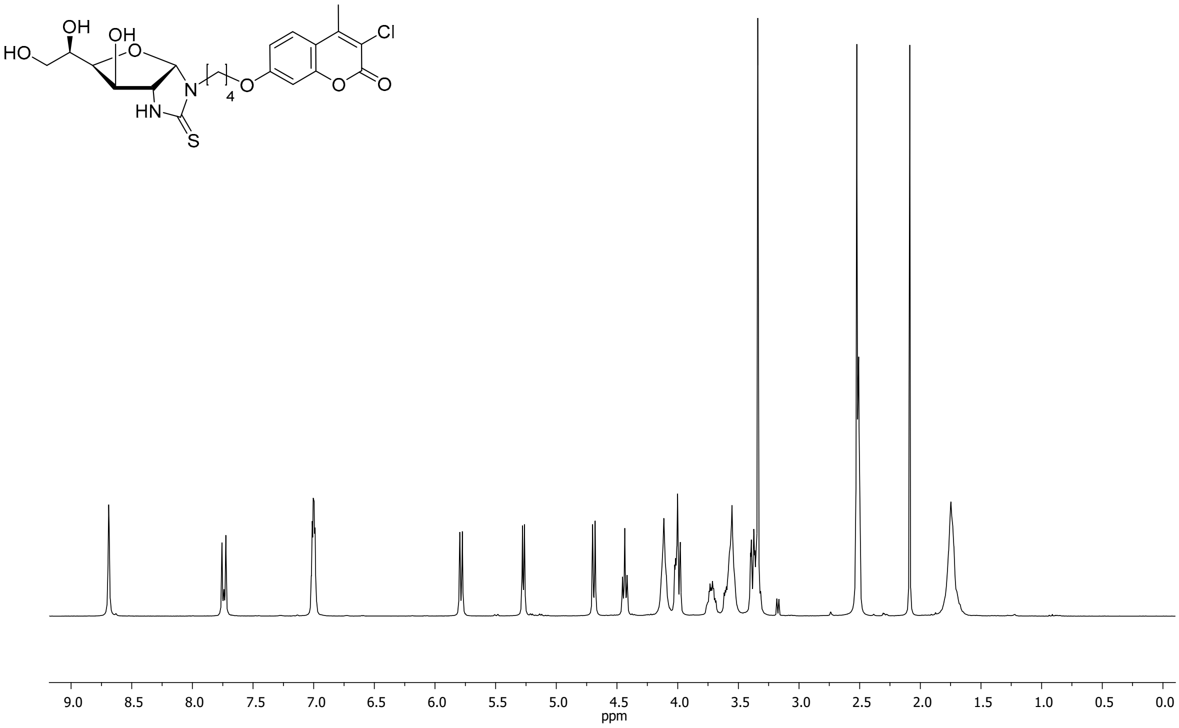

Figure S44. ^{1}H-NMR spectrum of **24h** (300 MHz, DMSO-d_6)

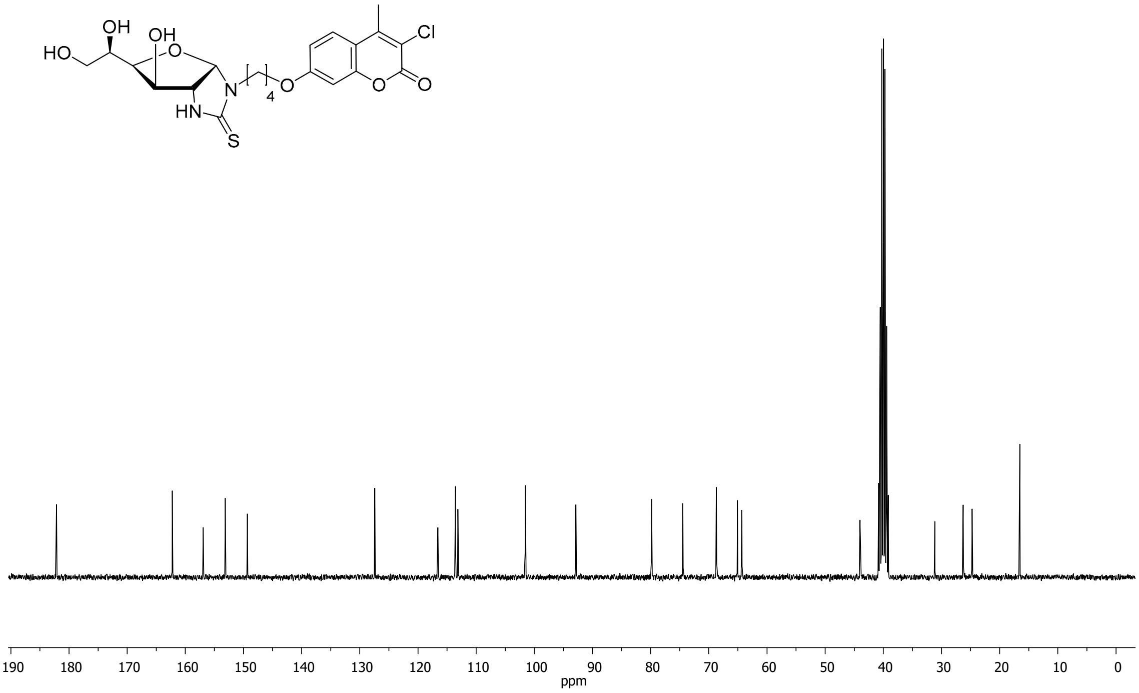

Figure S45. ^{13}C-NMR spectrum of **24h** (75.5 MHz, DMSO-d_6)

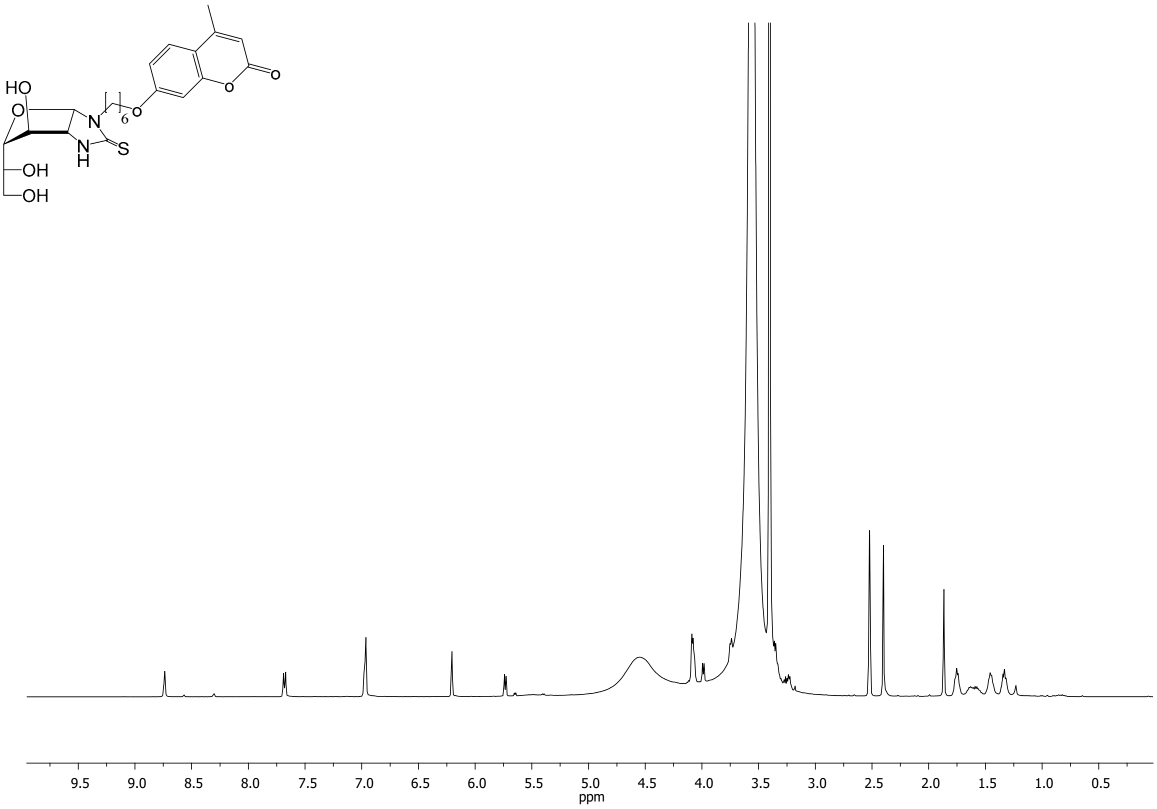

Figure S46. ^{1}H-NMR spectrum of **26** (500 MHz, DMSO-d_6)

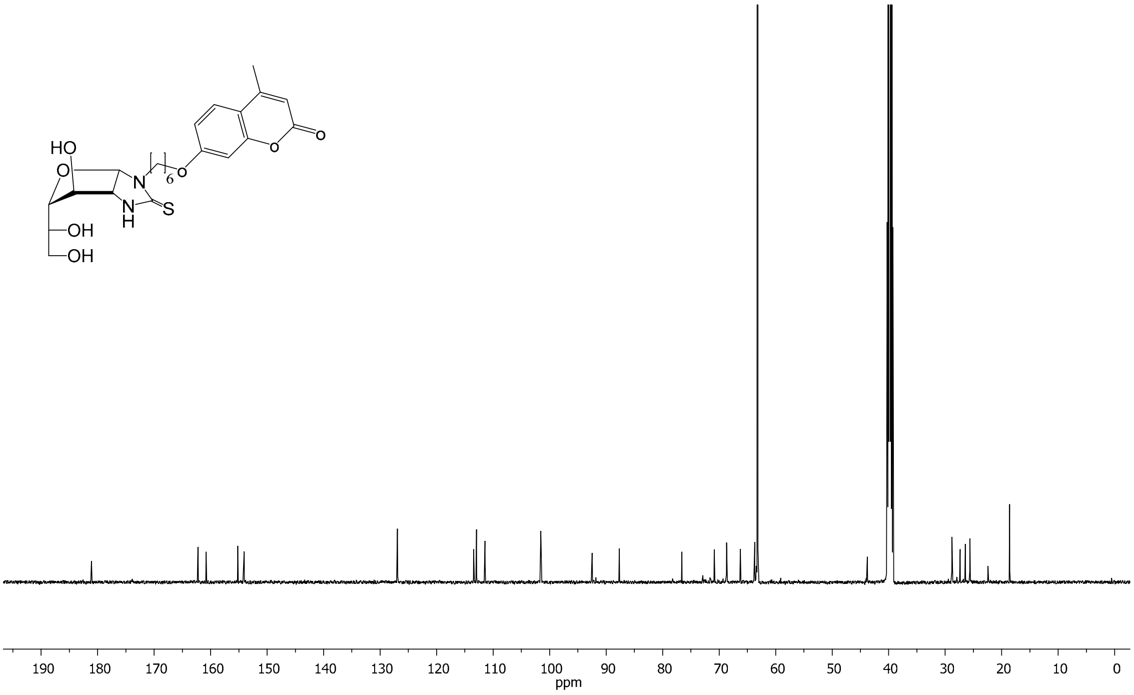

Figure S47. ^{13}C-NMR spectrum of **26** (125.7 MHz, DMSO-d_6)
